# Tracing the spatial imprint of Oldowan technological behaviors: A view from DS (Bed I, Olduvai Gorge, Tanzania)

**DOI:** 10.1371/journal.pone.0254603

**Published:** 2021-07-12

**Authors:** Fernando Diez-Martín, Lucía Cobo-Sánchez, Adrian Baddeley, David Uribelarrea, Audax Mabulla, Enrique Baquedano, Manuel Domínguez-Rodrigo

**Affiliations:** 1 Department of Prehistory and Archeology, University of Valladolid, Valladolid, Spain; 2 Institute of Archaeology, University of Cologne, Köln, Germany; 3 Institute of Evolution in Africa (IDEA), University of Alcalá, Madrid, Spain; 4 School of Electrical Engineering, Computing and Mathematical Sciences, Curtin University, Perth, Australia; 5 Department of Geodynamics, Stratigraphy and Paleontology, Complutense University, Madrid, Spain; 6 Department of Archaeology and Heritage Studies, University of Dar es Salaam, Dar es Salaam, Tanzania; 7 Regional Archaeological Museum of Madrid, Alcalá de Henares, Spain; 8 Area of Prehistory, University of Alcalá, Alcalá de Henares, Spain; Sapienza University of Rome: Universita degli Studi di Roma La Sapienza, ITALY

## Abstract

DS (David’s site) is one of the new archaeological sites documented in the same paleolandscape in which FLK 22 was deposited at about 1.85 Ma in Olduvai Gorge. Fieldwork in DS has unearthed the largest vertically-discrete archaeological horizon in the African Pleistocene, where a multi-cluster anthropogenic accumulation of fossil bones and stone tools has been identified. In this work we present the results of the techno-economic study of the lithic assemblage recovered from DS. We also explore the spatial magnitude of the technological behaviors documented at this spot using powerful spatial statistical tools to unravel correlations between the spatial distributional patterns of lithic categories. At DS, lavas and quartzite were involved in different technological processes. Volcanic materials, probably transported to this spot from a close source, were introduced in large numbers, including unmodified materials, and used in percussion activities and in a wide variety of reduction strategies. A number of volcanic products were subject to outward fluxes to other parts of the paleolandscape. In contrast, quartzite rocks were introduced in smaller numbers and might have been subject to a significantly more intense exploitation. The intra-site spatial analysis has shown that specialized areas cannot be identified, unmodified materials are not randomly distributed, percussion and knapping categories do not spatially overlap, while bipolar specimens show some sort of spatial correlation with percussion activities.

## Introduction

Lithic artifacts are the most conspicuous expression of past human behaviors. Because of their persistence in the archaeological record, stone implements have traditionally constituted a recurrent resource for research in the archaeology of human origins. The specialized literature is replete with publications aiming at disentangling the complex dynamics of ancient technological behaviors [1] that, as Nelson [2] explains, encode a number of behavioral responses to environmental conditions, resource availability, and economic -or even social- strategies. Site-oriented ESA lithic studies are primarily focused on issues such as raw material procurement and selection [3, 4], reduction sequences and tool maintenance strategies [5–9], tool use [10, 11] or, in order to establish referential models for archaeological interpretation, actualistic and experimental studies [12–17]. However, studies integrating the spatial dimension with lithic analyses are not very common.

The technological component of ancient human behaviors inevitably bears a spatial imprint, based on the specific spatial properties of the debris discarded across the space. This signature emerges at semi-local or regional scales, when discard behaviors inform us on the structure of landscape occupation [18–23], when raw material and artifact flow across the landscape are used to infer mobility patterns [24–27] or when synchronic/diachronic inter-assemblage variability explores the modulation of the techno-functional and economic use of the territory [28]. At a site scale [29], the spatial dimension of technology has only been tangentially explored in the archaeology of human origins. Despite being a dynamic field of research, intra-site spatial analyses in ESA contexts have experienced limited methodological development. Procedures currently implemented by micro-spatial studies, in which the integration of data provided by soil micromorphology, archaeo-stratigraphy, lithic conjoining and identification of raw material units in order to identify minimal behavioral entities in archaeological palimpsests [30–36] remain marginal in African Stone Age studies.

Among the reasons that may explain the absence of a high-resolution archaeology methodological approach [31, 37, 38] in ESA contexts, the effect of disturbance processes [39], the lack of time resolution in Lower Pleistocene contexts [30, 40–45] and the claimed palimpsest nature of most archaeological accumulations [30, 46, 47] should be considered as factors that have limited progress in the use of spatial perspectives to further understand early anthropogenic accumulations. On top of these issues, a pessimistic environment, based on the realization that archaeological time and ethnographic time tend to represent non-concomitant variables [30, 48], has prompted the current abandonment of those socio-economic and functional models previously used to build interpretative frameworks for early Pleistocene archaeological sites [24, 39, 49–54]. This abandonment of social and functional interpretations [55] represents a further obstacle for a renovation of intra-site spatial heuristics in the archaeology of human origins.

Taking as reference the seminal work undertaken by Kroll in Koobi Fora [56, 57], most ESA studies aimed at interpreting the spatial component of lithic accumulations have been carried out as part of a much broader framework, involving post-depositional evaluations, vertical distribution inspections, refitting studies, and descriptive analyses of horizontal association. Very commonly, researchers have used intra-site spatial analyses as a proxy to infer site integrity [58–66]. Lithic refitting studies have been used to evaluate vertical integrity as well as to represent horizontal spatial associations [5, 7, 57, 59, 63, 67–69]. Archaeo-stratigraphic procedures, the most useful way to dissect archaeological palimpsests, have been very rarely applied for the identification of archaeo-units and the use of these units as reference criteria for assemblage grouping and lithic analysis [63, 65, 70]. On occasions, with the application of spatial analytical tools, horizontal associations have mainly focused on the identification of distributional randomness and on a variety of density, cluster and distribution analyses. These works have mostly produced preliminary descriptive and thematic maps of density and horizontal class associations [63, 65, 71–75].

In the present study we attempt to go a step further in the goal of unravelling the spatial magnitude of ancient technological behaviors. By exploring in detail the spatial correlations and interdependences between lithic categories [55], our aim is to maximize the spatial component of the technological traces preserved in the archaeological record. We also intend to open a research perspective in which more normative lithic studies are integrated within their spatial dimension. Thus, this procedure can emerge as a complementary analytical routine for lithic studies and for inferences in the archaeology of human origins. Although perspectives of this kind have been already entertained [71], in this paper we use non-parametric approaches and nearest-neighbor perspectives to further explore statistical correlations that may unravel the spatial component of the most conspicuous technological behaviors that emerge from the Bed I archaeological site of David’s Site (Olduvai Gorge). Here, complex multivariate statistics have successfully been applied for the analysis of spatial patterning [71, 76, 77].

The use of the archaeological record unearthed at DS for a study of this kind is all but casual. Archaeological remains from Bed I at Olduvai Gorge are generally preserved in largely undisturbed contexts, and constitute a referential record for studying the behavior of early *Homo* [78, 79]. DS constitutes the largest documented early Pleistocene site in East Africa (554 m^2^). A recent taphonomic and spatial analysis of the faunal assemblage from this site has shown that the assemblage is autochthonous and that it was not significantly altered by postdepositional processes [77]. The results of this study reveal that the assemblage formed as a result of the transport of mostly complete fleshed carcasses to the site by hominins and that carnivore activity was marginal. The taphonomic study concludes that the site may have been used by hominins as a central place with easy access to water sources and herbivores and that the acquisition and transport of large carcasses probably relied on a high degree of group cooperation, since hominins not only obtained and transported food collectively, but it seems they also engaged in the collective consumption of meat resources at specific areas of the site [77].

These findings confirm earlier interpretations about hominin behavior drawn from previous analyses of FLK 22, a site that played an especially relevant role in reconstructions of early human behavior. For many years, FLK 22 represented the only anthropogenic accumulation in Bed I and the most solid proof of a shift toward higher behavioral complexity in early *Homo*. Other Bed I sites have been interpreted as palimpsests or carnivore accumulations [78]. Fortunately, apart from DS, the discovery of two additional anthropogenic sites in the same paleosurface as FLK 22 that are currently under study, PTK (Philip Tobias Korongo) and AGS (Alberto Gómez’s Site), reinforce the impression that Oldowan hominins actively and regularly obtained and shared meat resources in central places [55, 76, 77]. In this framework, these hominin-made assemblages, and DS in particular, given its exceptional preservation and large size, probably represent the most suitable archaeological record to integrate both lithic and spatial data in order to undertake a broader reconstruction of the technological behaviors preserved in Oldowan contexts.

## Materials and methods

### DS (David’s site), Bed I, Olduvai Gorge

**Fig 1:**
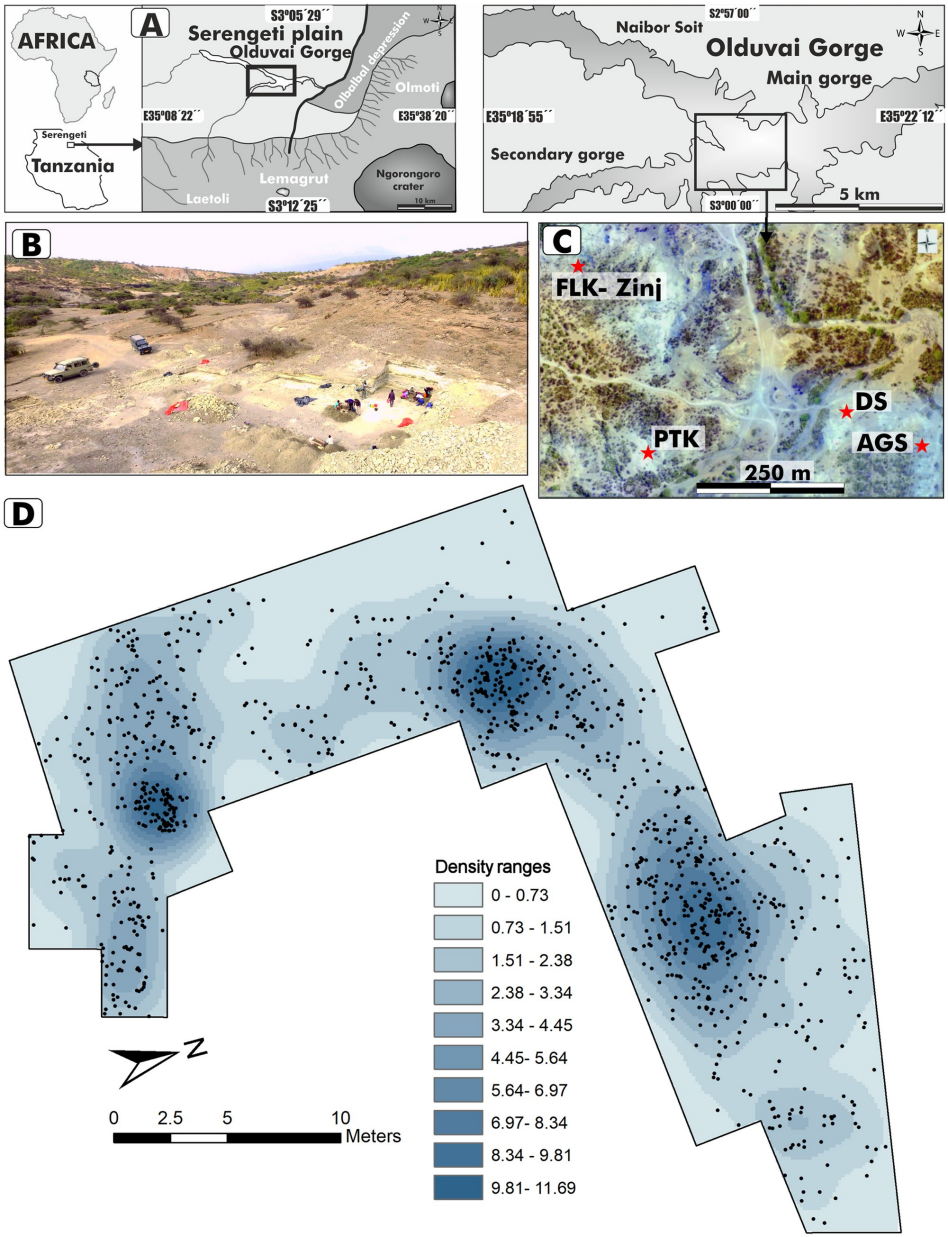
David’s site (DS). Location (A, C) and panoramic view (B). D. Horizontal spatial distribution and density map of lithic specimens (n = 1,229) recovered at DS. Bandwidth was estimated using the likelihood cross-validation function, which assumes an inhomogeneous Poisson process. The panoramic view (B) and the orthophotography (C) were obtained by our team (TOPPP) with an unnamed aerial vehicle. The maps (A) have been specifically drawn by us for this work.

DS is located to the south of the confluence of the main and side branches of Olduvai Gorge (**Fig 1**). The site was discovered by The Olduvai Paleoanthropology and Paleoecology Project (TOPPP) in 2014, exposed underneath the dirt road that led to the well-known HWK site complex [55]. DS shows a significant accumulation of fossil bones and lithics occurring on the same extensive paleosol as the classical Level 22 at FLK, in Bed I [49]. FLK Zinj level is deposited in a significant portion of Olduvai Gorge and it has been exposed in a number of new sites discovered by TOPPP, such as AMK, PTK, AGS, and DS [76, 77, 80, 81]. The FLK Zinj 22 paleosurface containing the archaeological occurrences unearthed in DS is a <20 cm thick green waxy clay and vertical unit. Lateral facies changes are responsible for the sporadic presence of a more siliceous clay type [79, 81]. The FLK Zinj unit is bracketed between Tuff IB, dated to 1.848 ± 0.008 Ma, and the overlaying Tuff IC, that caps the archaeological horizon at 1.848 ± 0.003 Ma [82]. Thus, archaeological material accumulated on the FLK 22 landscape over a short period of time [83]. Regarding DS, up to 2018, an area of 554 m^2^ has been opened and 5,700 archaeological items have been retrieved from the excavated window, representing a mean density of 10 items/m^2^. The bulk of the assemblage is constituted by fossil bones (n = 4,252), while stone tools represent 25% of the unearthed sample (n = 1,448). At DS, as in FLK22, two archaeological levels have been identified: 22A and 22B [79, 84]. While lowermost Level 22B contains a significant archaeological collection that includes a total sample of 1,320 lithic artifacts, Level 22A is poorly represented by a meager collection of 128 lithic specimens (Fig A in S1 File). Due to the small size of its lithic sample, and for the sake of analytical consistency in the spatial analysis, Level 22A has been excluded from our study. This work presents the technological and the intra-spatial association analyses of 1,229 lithic specimens recovered from Level 22B up to the 2017 field season (28 pieces sent to the laboratory for various analyses are excluded from this count). Our studied sample also excludes the 63 specimens recovered during the 2018 field season.

All necessary permits were obtained for the described study, which complied with all relevant regulations (provided by the Tanzania Commission for Science and Technology and Antiquities Department of the United Republic of Tanzania). The archaeological specimens used for this study are publicly deposited in the Aguirre-Mturi Research Station of Olduvai Gorge (Ngorongoro Conservation Area, Arusha district, Tanzania). All relevant data used for this work are included in this paper and in its S1 File.

DS was excavated over the course of five fieldwork seasons. Excavations proceeded quickly during the first two years, because the archaeological deposit lay only a few centimeters below the surface. Archaeological remains were carefully excavated and remains larger than 2 cm were plotted with a total station. The 3x3 m trenches were stereo-photographed prior to recovering the remains in order to obtain a photogrammetric 3D reconstruction of the deposited materials as they were when they were uncovered. Compasses and inclinometers were used to measure azimuth and plunge values respectively of the archaeological items. Sediment buckets were sieved in 5 mm and 3 mm meshes. Since the density of remains increased towards the south [55], where the archaeological deposit was covered by a much thicker sedimentary stratigraphic sequence, pneumatic hammers, picks and shovels had to be used to remove the sterile deposits. At the end of the 2016 field season, most of the paleosurface had been unearthed. In 2017 and 2018, several additional trenches were excavated at the edges of the site with the intention of demarcating its limits. These trenches yielded lower densities of archaeological materials, and the limits of the site could thus be confidently established. The central area of the site is delimited by the deposition and subsequent erosion of Ndutu unconformable sediments, which exposed the underlying clay level to rain, and the use of the area as a road. The southern edge of the excavation is characterized by a change in facies from clayey to silty sediments, which probably indicates a change in the paleolandscape. The almost complete absence of bone remains at the southeastern limit of the site could be due to the presence of more water in this area–reflected in a greater presence of large carbonate nodules–that could have affected the preservation of fossils. The remaining areas are limited by the lava flows at the bottom of Bed I that were already part of the paleolandscape when hominins created the site.

The four fully anthropogenic sites that have been discovered to date in Bed I (FLK Zinj, DS, PTK, and AGS) all stem from the paleosol underlying Tuff 1C, which contains a clay stratum (<20 cm) that can be traced laterally at the junction of the gorge and at both ends of its trajectory in areas that lay close to an ancient lake. The stratigraphic sequence at DS presents some peculiarities with respect to the FLK area. At DS, the Chapati Tuff is reworked and, in some areas, eroded, although in most parts of the site Level 22B overlies the Chapati Tuff with a discrete and sharp contact [84]. Level 22 consists of two sublevels (Level 22A and Level 22B) each containing separate archaeological assemblages. Level 22B is a ~10 cm-thick olive gray (5Y 4/2) clay. In this level, archaeological remains are commonly found in the lowermost ~5 cm. The clay mineral micro-textures and the absence of large clay aggregates show that Level 22B was deposited under very low energy conditions [84]. On top of 22B an intercalated, discontinuous <5 cm silty unit, Level 22 Silt, is found at DS and surroundings [79, 84]. The deposition of this level, however, does not seem to have altered the position of the archaeological remains to a significant extent. Level 22A is a ~10 cm dark olive gray (5Y 3/2) earthy clay, discontinuous, low energy deposit [84] which overlies Level 22 Silt in the northern areas of the site, is commonly absent in the center of the site, but overlies Level 22B in the southern grids of the site. Archaeological remains from this level are also found in the lowermost ~5 cm of the level. The airfall tephra Tuff 1C was deposited conformably over Level 22A and 22 Silt [79, 85] (Fig B in S1 File).

Geoarchaeological studies have repeatedly emphasized the low-energy depositional environment dominating the lower and middle sequence of Bed I in the Zinj paleolandscape [79]. Mineralogical and geochemical lateral variations throughout the FLK Zinj paleolandscape suggest that fresh water entered the system from the surroundings of DS during the deposition of both levels 22A and 22B [84]. This suggests that hominins at DS may have had regular access to fresh water and herbivores. Furthermore, phytolith analyses have shown that, like FLK Zinj and PTK, DS was located in a wooded environment, which may have provided refuge from carnivore predation.

**Fig 2:**
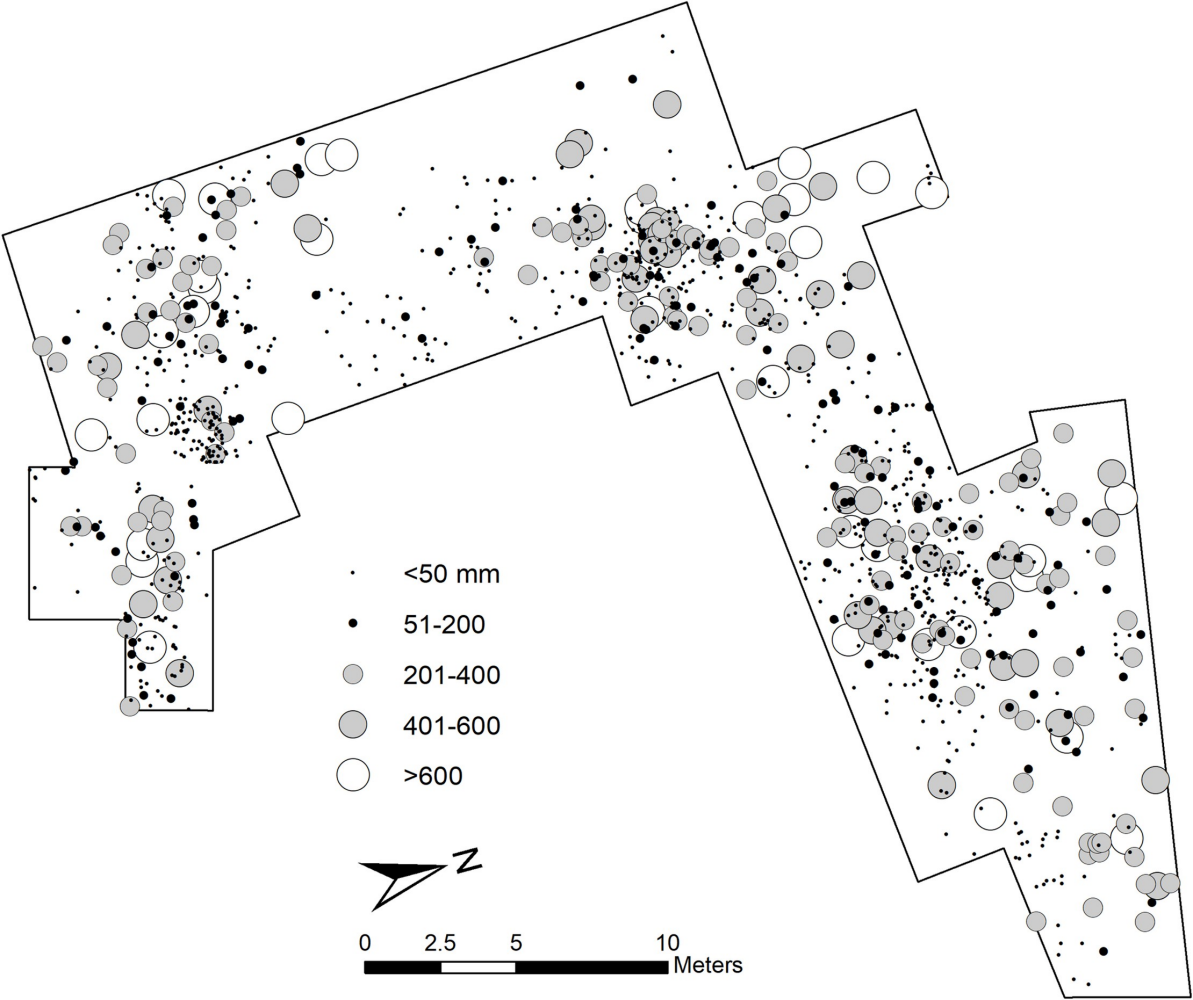
Distribution map of artifact mass (weight) classes at DS.

As explained above, the sedimentary matrix in which the DS assemblage was recovered is composed of clay and silty clay, which points to a very low-energy depositional environment. Partially polished and abraded specimens were very rare (less than 0.3%), as was evidence of water-induced and chemical modifications on bone surfaces (less than 0.5%). Subaerial weathering was almost non-existent (99.9% of specimens fall into stage 0 [86]), which means that the assemblage formed probably in one or two years depending on the amount of dense vegetation present at the site. Importantly, the completely uniform distribution of the orientation of bones including long bone shafts and the overwhelming presence of small bone specimens shows that the DS assemblage lacks any evidence indicative of transportation by water flows. In this regard, previous analyses of the bone distribution and bone size representation [77] have shown that ≤20 mm specimens represent more than half of the bone assemblage. Furthermore, specimens ≤30 mm constitute 80% of the sample. This distributional and absolute predominance of the small fraction in the excavated window is indicative of minor post-depositional effects on the bone sample. Fig 2, showing the spatial distribution of lithic artifacts by mass classes (a variable strongly related to size), agrees with the information provided by the faunal remains. In all instances, DS is characterized by the predominance of small and light specimens (both lithics and fossil bones) scattered throughout the trench, which precludes significant size-sorting of the archaeological aggregate due to natural agents [39, 87, 88]. The exceptional preservation of the site therefore suggests that the spatial properties of the assemblage, as hominins might have left it, may have remained intact to a great extent.

The results of the taphonomic study of the ungulate remains from Level 22B, specifically the analyses of skeletal part abundances, bone breakage patterns and bone surface modifications, also reveal that the assemblage probably formed as a result of the transport of several complete small and medium-sized carcasses to the site by hominins and that carnivore activity was marginal. Machine-learning analyses of bone breakage patterns show that hyenas contributed to some degree to bone breakage at the site, but most fracturing resulted from hammerstone breakage [77]. The documented systematic presence of cut marks on all anatomical portions of the carcasses, including areas that are usually depleted of flesh after carnivore defleshing, shows that hominins were removing complete muscles from the bones rather than flesh scraps, which is indicative of primary access to carcasses. The multivariate statistical analysis of the bovid mortality profiles reveals a predominance of prime adult prey and points to ambush hunting as the most probable carcass acquisition strategy [77].

### Lithic analysis

We have identified the following lithic categories in our study [13, 14, 65, 89, 90] (**Table 1**): 1) Unmodified materials consist of complete/broken cobbles and cobble fragments devoid of anthropogenic interaction; 2) Percussion elements include all the array of lithic specimens showing percussion damage on their surfaces (such as scarring and battering) and, thus, associated with active or passive percussive tasks (complete/broken hammerstones and anvils), including by-products resulting from percussion, such as flakes and fragments. Percussion flakes have been identified as thin positives with battered cortical areas and/or aberrant ventral areas (including convex or curved ventral surfaces, and absence of platform surfaces). Modified Battered Blocks (MBB), quartzite cuboid or spherical specimens purportedly related to percussion actions [17] are also included in this group; 3) Cores. Handheld cores have been classified according to the interaction of three parameters: a) Number of surfaces exploited (unifacial, bifacial, and trifacial/multifacial); b) Striking platforms observed (unipolar, bipolar, multipolar); c) The arrangement of knapping series (one direction, two unorganized directions, opposed longitudinal, opposed lateral, orthogonal, centripetal). We have also recorded the number of negative scars and, when possible, length and width of up to three negative scars. Bipolar cores have been classified according to the variety of technical traits and rotation patterns typically related to anvil percussion [13, 89]; 4) Detached specimens include diagnostic bipolar flakes as well as handheld complete and broken flakes. We have also recorded reduction stages, type of striking platforms (cortical, uni-faceted, bi-faceted, multi-faceted, linear, punctiform, broken) and dorsal patterns; 5) Retouched flakes are specimens transformed by retouch in normative types; 6) We have identified choppers as core-like specimens that, according to the form and location of their cutting edge (rectilinear, acute and/or with signs of secondary trimming), can be potentially interpreted as intentionally shaped tools. To include specimens in this category we have considered the following criteria: a) Presence of rectilinear and acute (>75°) distal or lateral cutting edge; b) The identification of secondary trimming reinforcing cutting edge segments; 7) Waste refers to the by-products of the knapping process, including core rejuvenation flakes, non-diagnostic flake fragments, shatter (blocky and angular fragments), debris (≤25 mm), and core-like fragments.

**Table 1:** Number and percentage of lithic artefacts sorted by lithic category and raw material type.

Lithic categories	Raw material				Total	
	B	P	Q	O	n	%
**Unmodified**	**123**	**3**	**6**	**1**	**133**	**10.82**
Cobbles	111	3	4	1	119	
Broken cobbles	7		2		9	
Cobble frags.	5				5	
**Percussion**	**33**	**10**	**11**		**54**	**4.39**
Hammerstones	26	6	1		32	
Brok. hammers	1				1	
Flakes/ frags.	4	4	4		12	
**Anvils**	2				2	
MBB			6		6	
**Cores**	**108**	**21**	**47**	**1**	**177**	**14.4**
Handheld cores	108	21	33	1	163	
Bipolar cores			14		14	
**Detached**	**55**	**14**	**280**	**4**	**353**	**28.72**
Handheld flakes	42	11	171	3	227	
Broken flakes	13	3	97	1	114	
Bipolar flakes			12		12	
**Ret. flakes**	**6**	**4**	**30**		**40**	**3.25**
**Chopper-cores**	**2**	**1**	**1**		**4**	**0.32**
**Waste**	**29**	**11**	**422**	**6**	**468**	**38.07**
Core flakes	1	2	5		8	
Flake fragments	2	3	104	2	111	
Shatter	5		96		101	
Debris	4	1	172	1	178	
Fragments	17	5	45	3	70	
**Total n**	**356**	**64**	**797**	**12**	**1,229**	
**Total %**	**28.96**	**5.20**	**64.84**	**0.97**		

B = basalt; P = phonolite; Q = quartzite; O = Other, gneiss, chert and hyaline quartz.

### Intra-site lithic spatial analysis

Following the above-mentioned technological categorization of artifacts, we performed a spatial statistical analysis of the lithic assemblage, with the aim of exploring patterns of spatial distribution and correlation between the different lithic categories. The analysis mainly consisted in estimating and comparing the spatially varying intensity functions of different types of lithics using non-parametric methods, and occasionally applying approaches based on nearest-neighbors to find possible correlations between these distributions. The following spatial correlations have been considered: a) general observations: spatial distribution and correlation between raw materials, mass, and lithic categories; b) unmodified material: spatial correlations between unmodified specimens and modified cobbles, test cores and percussion material; c) percussion: spatial correlations between specimens directly included in percussion categories (hammerstones, percussion flakes, anvils and MBB), multifacial/multipolar cores or cores with signs of percussion; d) bipolar knapping: spatial correlations between specimens directly (bipolar cores, bipolar flakes) or indirectly (shatter or undetermined positives) related to bipolar knapping and percussion or freehand knapping (freehand cores and complete freehand flakes); e) freehand knapping: distribution of categories directly linked with freehand knapping, distribution of freehand flakes in different stages of the reduction sequence, and correlations between freehand cores and hammerstones, freehand cores and complete freehand flakes, types of cores (casually vs exhaustively exploited) and flakes in different stages of reduction; f) waste: spatial correlation between specimens included in waste categories (fragments, shatter, debris) and bipolar vs freehand reduction.

### Estimation of the spatially varying intensity of the lithics point pattern

All the following spatial statistical analyses were performed using the “spatstat” library in R software [91]. The variation in the intensity of lithics (number of artifacts per unit area) is very marked throughout the excavation window of DS (**Fig 1**). This suggests that the null-hypothesis of Complete Spatial Randomness (CSR) should be rejected. Complete Spatial Randomness means that the probability density function of an observed point pattern is constant over the study window and that points are independent, i.e. the observed pattern in one region is not influenced by the observations in other regions [91–94]. In order to confirm spatial inhomogeneity using formal tests, we divided the excavation window into five tiles of the same area and carried out a chi-square power divergence test based on quadrat counts [91, 95]. We also used the Hopkins-Skellam test, which calculates the nearest-neighbor distances and compares them with the nearest-neighbor distances of completely random point patterns [96]. The spatially varying intensity can be described as a function of spatial location and can be estimated from the data non-parametrically using kernel estimation with a particular smoothing bandwidth. A large bandwidth yields more smoothing, and a smaller bandwidth results in higher variance. The likelihood cross-validation function, which assumes an inhomogeneous Poisson process, was used to calculate the smoothing bandwidths of the density maps throughout the spatial analysis. For every spatial object that was created for each spatially observed variable, density maps were made using this bandwidth selection method.

A scan test [91, 97] was employed to look for evidence of high intensity inside circles of fixed radius and to test, using a likelihood ratio test statistic, whether the intensity in these areas is statistically different from the intensity outside the circles. The test can also be used to estimate the size of a statistically significant cluster. We performed this analysis to test whether the clusters of quartzite and basalt lithics were significant and to estimate their sizes.

Whenever the spatially varying type probabilities of a multi-type point pattern were of interest, we estimated the relative abundance of each type using cross-validation to select the appropriate bandwidth and drew tolerance contours around the regions where the estimated probability of a given type was significantly different from the average proportion [98]. A Monte Carlo test with 19 simulations was used to assess significance. The procedure is as follows: The point pattern is first randomly relabeled (i.e. the marks attached to the points are randomly permuted) and the type probability or relative risk is computed for the relabeled data. This is repeated n times, yielding n images of the probabilities of the mark types. A Monte Carlo test is computed at each pixel. We carried out 19 simulations, which produces p-values that are multiples of 1/20 = 0.05. We also performed Monte Carlo tests of spatial segregation of the two types of remains in question with 19 simulations [99, 100].

### Correlation and point inter-dependence

Usually correlation and inter-dependency between points is assessed through the use of empirical summary functions, such as the K-function. These are useful because they convey information across a range of spatial scales, but their weakness is that the intensity of the spatial point pattern has to be very accurately estimated. For certain types of inhomogeneity, the K-function and its relatives are not statistically robust, and caution is recommended. We chose to use the inhomogeneous version of the G-function [101], which calculates the nearest-neighbor distances, to determine the type of point pattern of some of the spatial variables. We used Cronie and van Lieshout’s [102] criterion for the intensity estimate and compared it with the G-function of an inhomogeneous Poisson process made with the intensity function of the DS lithic pattern. Importantly, the procedure used to compute an inhomogeneous G-function should be exactly the same for the simulated Poisson pattern as for the data. Testing whether the deviation from the inhomogeneous Poisson line is statistically significant involves creating envelopes with 39 Monte Carlo simulations of inhomogeneous Poisson processes using the intensity function of the DS point pattern. This guarantees that the DS and the inhomogeneous Poisson curves are comparable, and that the corresponding envelopes support a valid Monte Carlo test of the null hypothesis.

Since spatial variation in the DS point pattern is extreme and hard to estimate accurately, it is a challenge to determine the type of correlation between different types of remains using methods derived from these summary functions. Approaches based on nearest neighbor distances are more robust against spatial variation, because they do not involve estimating the intensity of the point pattern.

We thus employed the nearest neighbor equality function, which is a newly developed tool that is analogous to the inhomogeneous cross-type K-function, but uses counts of nearest neighbors of a certain type. The method counts the proportion of neighbors of a certain type against the order of the neighbor, and is based on the nearest-neighbor correlation, a robust method that can be applied to stationary and non-stationary processes. The non-cumulative version of this function is equivalent to computing the nearest-neighbor correlation. Yet, when the cumulative proportion is calculated, the graph shows the fraction amongst the kth nearest neighbors, which have a specified type. This function is very convenient, because it does not operate with the spatial location of the points or their distances, and is therefore not dependent on the intensity of the point pattern. The envelopes are generated by keeping the positions of the points fixed and shuffling only the labels of the points. The graph of this function shows the observed proportion of neighbors of the same type to the type of origin at each neighbor step. When the observed line falls above the expected line, then points of the same type tend to be closer together than points of different types. When the observed line falls outside the grey envelopes, the relationship is statistically significant.

### Evaluation of the spatial uniformity of the overall technological pattern at the three high intensity areas using multinomial logistic regression

A further way to assess the spatial distribution of lithic artefacts at DS is to consider if the three documented high intensity areas share a similar technological pattern, i.e. whether they contain similar proportions of the same types of lithics or, in contrast, represent different technological areas. The latter would entail that hominins carried out different activities related to tool use and manufacture in separate areas of the site. In order to address this question, we followed a method that was proposed by Smith et al. [94] and which has also been successfully applied to the taphonomic variables associated with the spatial point pattern of the faunal assemblage from DS [77]. The method consists in splitting the spatial study window into different subregions and using multinomial logistic regression to identify the variables that present statistically significant spatial variation between the three areas. We used the K-nearest neighbor graph to connect the points with segments to their K nearest neighbors and in order to find an appropriate subdivision of the point pattern through the emptiest areas of the spatial object. Following recommendations by others [93, 94], we used a K value of 3. We cut the spatial object into three similarly sized areas (A, B, and C), each one containing one of the high intensity cluster areas (**Fig C in** S1 **File**). These three areas act as the dependent variable. We carried out two separate multivariate regression models, each one with three of the most important independent categorical variables that were previously explored separately from a technological and spatial perspective, as described above (**Table A in** S1 **File**).

In multinomial logistic regressions one of the categories of the dependent variable is chosen as the reference category (in this case Area A) and coefficients and their p-values, as well as the associated standard errors are estimated for the other categories of the dependent variable. The odds ratios are interpreted in relation to the reference category. The multinomial logistic regression model was carried out using the “multinom” function from the “nnet” R library [103].

## Results

### Techno-economic study

**Table 1** shows the number of lithic implements sorted by lithic category and raw material type, while **Fig 1** shows the spatial distribution and density pattern within the excavation area. The rocks identified at DS are mostly lavas (basalt and phonolite) and Naibor Soit quartzite. Other rocks, such as gneiss, chert and hyaline quartz are very marginally represented. The main source of quartzite is the inselberg of Naibor Soit, located about 3.5 km north of the confluence of the two gorges. In this promontory, quartzite originates with its host rock (gneiss of granitic composition) in pegmatitic veins and dikes. Tabular fragments of this quartzite can today be found in abundance on the slopes of the inselberg. Macroscopically speaking, this rock is polycrystalline quartz with a coarse-grained texture responsible for its irregular, heterogeneous breakage pattern. Regarding igneous rocks, the Engelosin volcano (about 7 km north of the Main Gorge) is the main source of greenish, slightly porphyritic, and very fine-grained phonolites. The Lemagrut volcano, located 2 km from the southern edge of the gorge, is the main source of mostly medium to fine-grained basalts. Although the primary source of volcanic materials (mostly in the form of boulders) is located on the volcano slopes, secondary sources are easily available as rounded cobbles and pebbles transported by fluvial channels into the basin [104–107].

Most of the lithic specimens (n = 1,129, 91.86%) have been preserved in mint fresh condition (R0), with no evident signs of polishing on surfaces, edges and ridges. A small number of specimens (n = 64, 5.2%) show moderate abrasion (R1), while artifacts showing intense roundness (R2) represent a negligible 0.32% (n = 4). According to this data, post-sedimentary processes related to water flux agents or weathering do not seem to have played a significant role in the aggregate formation [108, 109], although the very few specimens showing intense polishing might bear a more complex taphonomic history, in which a few recycling episodes cannot be ruled out. A small number of items (n = 32, 2.6%) show diagenetic alterations related to physico-chemical disturbance. The bulk of these specimens are unmodified cobbles (n = 18, 56.2%).

Overall, when taking into consideration size and mass, the assemblage can be defined as small-sized and light-duty. Mean maximum length is 43.52 mm (range = 9–204, sd = 25.59). **Fig 2** shows the spatial distribution of specimens by mass classes. Most of the assemblage, 855 items (69.56%), weigh ≤50 g (mean = 11.38, sd = 11.27), while only 41 pieces (3.33%) show mass values ranging between 601 and 3895 g. A Kruskal-Wallis rank sum test (p-value = <0.0001) shows that there are significant statistical differences in mass when specimens are classed by raw material type. Volcanic materials, particularly basalt, tend to be heavier than quartzite and other rock types (**Table B in** S1 **File**). In relative numbers (i.e. count of specimens distributed in lithic categories), the predominant rock type in DS is quartzite (65.93%), followed by volcanic rocks (basalt and phonolite). However, a more exact evaluation of raw material contribution to this site comes from mass. If we take in consideration rock types sorted by mass, volcanic materials indisputably outnumber quartzite, as 113.8 kg of lavas were discarded at the spot, contrasting with the 19.7 kg of quartzite**. Fig D in** S1 **File**, showing the percentage contribution of mass to lithic categories and raw materials, unravels a more accurate contribution of volcanic rocks and quartzite to the lithic assemblage. A significant number of basalt specimens (n = 99, 27.8%) can be identified as vesicular cobbles, which include many vesicles or cavities in their inner structure. Other raw materials, such as gneiss (n = 5, 0.4%), chert (n = 4, 0.32%), and rock crystal (n = 3, 0.23%) contribute very marginally to the sample (**Table 1**), altogether barely representing 1 kg of rocks.

#### Unmodified material

**Table 1** lists, sorted by raw material, the groups included in this category. The most abundant class, representing 89.47% of the total sample, is constituted by complete cobbles. Mean size and weight of the unmodified cobbles is shown in **Table C in** S1 **File**. Most cobbles are basalt (61 regular and 46 vesicular). Although a variety of forms have been documented (including amorphous, angular and flat), fine-grained, oval/rounded specimens predominate (49.6%). Rounded cobbles have also been documented on vesicular basalt (32%) and other raw materials (quartzite, phonolite and chert).

#### Percussion processes

Hammerstones are the most frequent tools in this category (**Table 1**). Most of them are fine-grained oval/rounded basalt cobbles, although phonolite and quartzite specimens are also represented. Battering damage is preferentially located on proximal/distal areas, and seldom on mesial/lateral areas. Scarring due to percussion has also been identified (**Fig 3.1, Fig E in** S1 **File**) There are six volcanic percussion flakes, showing aberrant ventral surfaces and cortical dorsal areas with signs of battering. The single quartzite hammerstone recovered is a cobble with a natural polyhedral shape showing intense and connected frosting on some edges. When comparing size and mass between complete unmodified cobbles and hammerstones (**Table C in** S1 **File**), an Anova test shows no significant differences in maximum length between the two samples (p = 0.0918), but points to differences in breadth (p = 0.049) and, particularly mass (both p = 0.00045) and thickness (p = 0.00010). If our analysis is focused on basalt, excluding other raw materials, similar differences emerge in thickness (p = 0.00096) and mass (p = 0.01).

**Fig 3:**
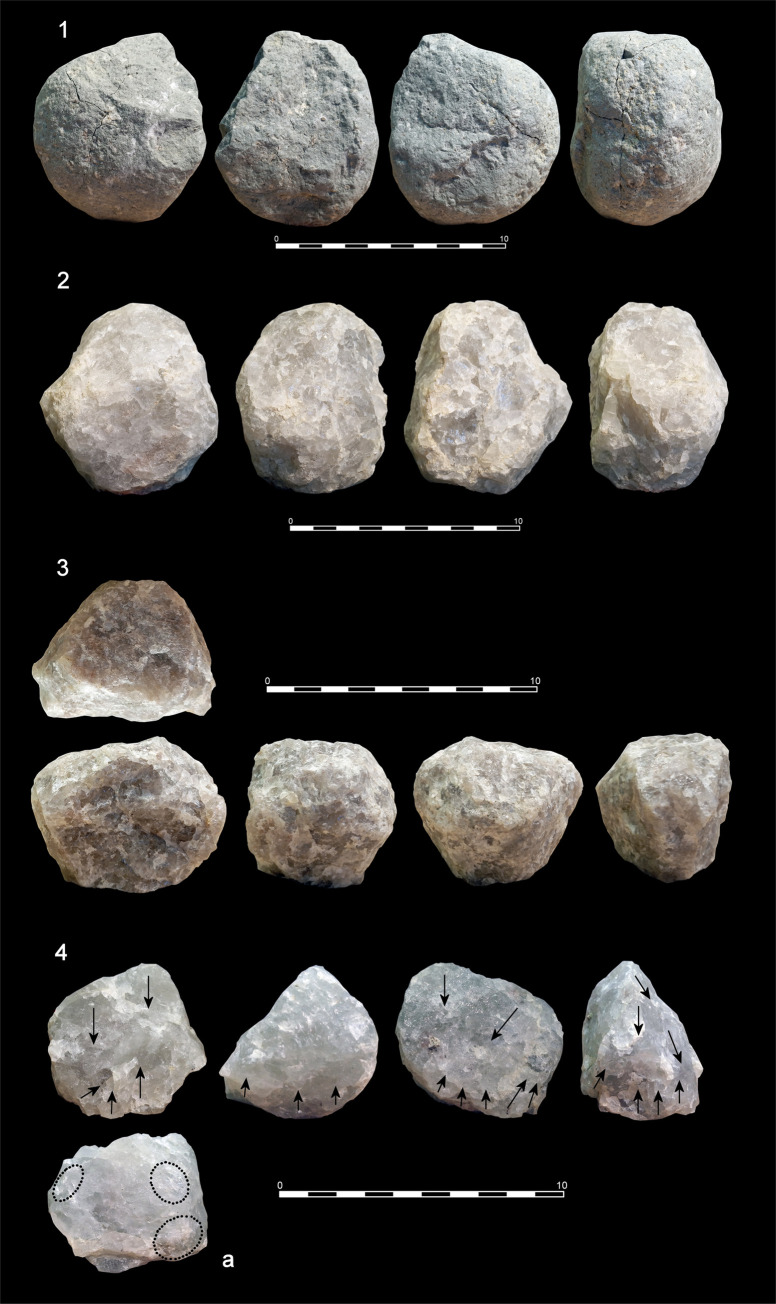
Percussion and bipolar specimens from DS. 1. Basalt hammerstone showing intense pitting and scarring due to percussion actions (74x66x48 mm, 312 g); 2. Quartzite MBB or subspheroid with intense signs of frosting (80x66x59 mm, 454 g); 3. Quartzite MBB or spheroid with intense signs of frosting and a plane of fracture due to percussion (71x58x57 mm, 343 g); 4. Quartzite core showing signs of bipolar load application and intense frosting on its base (4a).

The two identified anvils are a very heavy angular vesicular basalt boulder (3.8 kg) and a flat basalt cobble (735 g) showing intense separated pitting or battering around the central area of flat surfaces. A meager sample (n = 6) of MBB has been identified. These specimens are smaller and lighter than hammerstones (mean 62x54x48 mm, and 251 g), belong to a later stage of shape transformation and are rounded with intense battered surfaces and transformation of the original blank. Therefore, all of them fit well within the classical types of sub-spheroids (n = 4) and spheroids (n = 2) (**Fig 4.2 and 4.3**).

**Fig 4:**
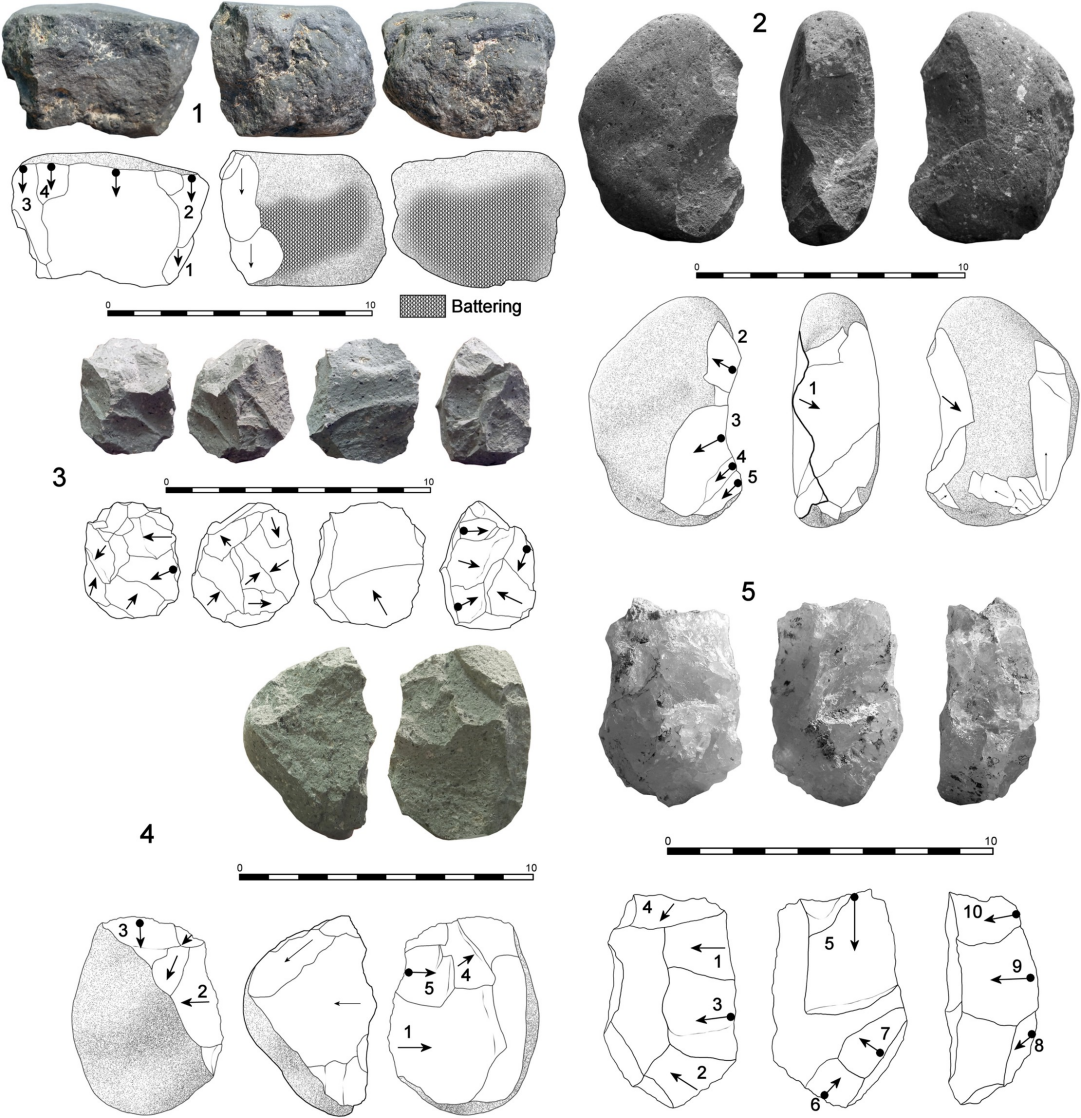
Cores from DS. 1. Unifacial linear (UL) core on basalt with intense battering due to percussion (67x59x48 mm, 322 g); 2. Bifacial linear (BL) core on phonolite, showing proximal scarring due to percussion (86x53x32 mm, 247 g); 3. Phonolite multifacial multipolar (MM) core in a final stage of exploitation (46x38x32 mm, 71g). Negative scars with step termination are abundant; 4. Bifacial alternating core (BA) on phonolite, following an alternating reduction pattern on both surfaces (67x53x42 mm, 180 g); 5. Bifacial orthogonal (BO) core on a quartzite fragment. In fact, the specimen shows series on three different planes, one of which presents a clear orthogonal pattern (65x42x29 mm, 103 g).

#### Cores

The core sample accounts for 14.4% of the assemblage. Meaningfully, a large number of the handheld specimens have been exploited from volcanic rocks (basalt and phonolite, 78.52%), while 20.85% correspond to quartzite and only one core has been exploited on gneiss. All the bipolar cores recovered from DS have been produced on quartzite (**Table 1**). Size and mass comparison between volcanic and quartzite cores show significant statistical differences in the variables maximum length (Wilcoxon rank sum test, p-value = <0.0001), maximum width (p-value = <0.0001), and mass (p-value = <0.0001). In all instances, volcanic cores are larger and heavier than quartzite cores (**Fig F in** S1 **File**). The following reduction patterns have been observed in handheld percussion (**Table 2**): a) Test (T) cores, the most abundant, are specimens showing few and unorganized negative scars; b) Unifacial linear (UL) cores, the second most common group, are constituted by short series of (at least three) adjacent removals, sub-parallel, and perpendicular with respect to the striking platform (**Fig 4.1**); c) Unifacial orthogonal (UO) cores are characterized by removals on a single surface that are arranged orthogonally in two or three short series; d) Bifacial linear (BL) cores, the third most representative group, show bifacial negative scars detached from the same striking platform, usually a natural ridge (**Fig 4.2**). This group displays great diversity, as series tend to be short, core rotation is limited, and bifacial detachments do not follow a structured sequence; e) Bifacial alternating (BA) cores differ from the previous group in that flakes are detached following a clear alternating bifacial pattern (**Figs 4.4** and **5.3**). Here core rotation tends to affect a larger portion of the perimeter; f) Bifacial orthogonal (BO) cores are specimens in which bifacial exploitation, significant blank rotation, and a selection of different striking platforms has allowed orthogonal arrangement of some of the detachment series (**Figs 4.5, 5.1** and **5.4**); g) Bifacial centripetal (BC) cores are represented by two specimens. In both cases bifacial reduction tends to alternation. Thus, no preparation of striking platforms and surfaces have been identified (**Fig 5.2**); h) Multifacial/multipolar (MM) cores are the fourth best represented group, in both volcanic and quartzite rock types. These specimens are characterized by short knapping series detached from multiple striking platforms and surfaces. The result of this reduction pattern is a characteristic polyhedral interaction of negative scars (**Fig 4.3**).

**Fig 5:**
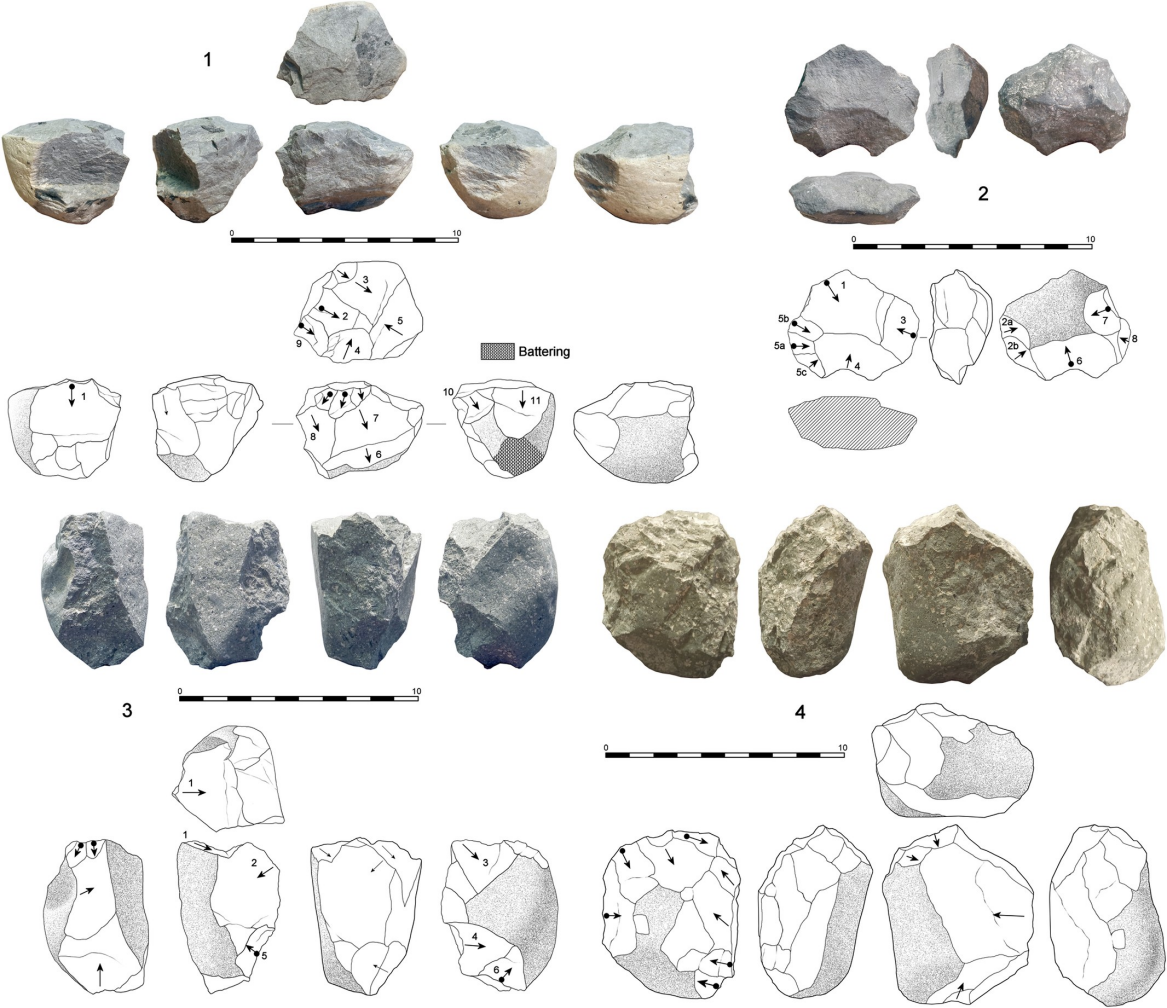
Cores from DS. 1. Bifacial orthogonal (BO) small core on basalt (53x41x39 mm, 123 g). The upper surface shows a series of removals with an orthogonal arrangement, while the thickness plane of the pebble has also been exploited. No hierarchy exists between the surfaces. Negatives with step termination can be observed. Intense signs of percussion have been observed at the globular base; 2. Bifacial centripetal (BC) small core on basalt, with no signs of hierarchization of surfaces. Upper face shows a centripetal arrangement of negatives and the specimen is in its exhaustion stage (56x43x23 mm, 66 g); 3. Bifacial alternating (BA) core on phonolite, showing an alternating sequence of scars detached from the same plane (60x52x42 mm, 176 g); 4. Bifacial orthogonal (BO) core on phonolite. One surface shows a bifacial arrangement of negatives. Step terminations are frequent and areas with percussion damage can also be observed (72x58x41 mm, 228 g).

**Table 2:** Distribution (number and percentage) of cores sorted by technique, reduction model and rock type.

Type	Rock type				Counts by type	
	B	P	Q	G	n	%
Handheld knapping						
T	48	2	5		55	33.74
UL	22	1	2	1	26	15.95
UO	2		2		4	2.45
BL	18	5	1		24	14.72
BA	1	4			5	3.06
BO	5	3	7		15	9.2
BC	1		1		2	1.22
MM	9	5	7		21	12.88
E	2		9		11	6.74
Subtotal	108	20	34	1	163	
%	66.25	12.26	20.85	0.61		
Bipolar knapping						
BP			14			
Total	108	20	48	1	177	
%	61.01	11.29	27.11	0.56		

Reduction model: T = test/unorganized; UL = Unifacial Lineal; UO = Unifacial orthogonal; BL = Bifacial lineal; BA = Bifacial alternate; BO = Bifacial orthogonal; BC = Bifacial centripetal; MM = Multifacial multipolar or polyhedrons; E = Exhausted cores. Rock type: B = Basalt; P = Phonolite; Q = Quartzite; G = Gneiss

**Fig G in** S1 **File** shows that volcanic rocks prevail in all reduction models. They predominate in unorganized/test (mostly regular basalts, n = 26 and vesicular basalts, n = 22), unifacial (UL, UO) and bifacial simple (BL, BA) reduction models. Although volcanic rocks are also more numerous than quartzite specimens in bifacial progressive (BO, BC) and multifacial core types, differences are more attenuated. On the contrary, exhausted cores are predominantly quartzite specimens (82% out of this category). A Chi-squared test (p-value = <0.0001) reveals significant statistical correlation between raw material and core type. More specifically, an MCA shows ample overlapping between exhausted cores (E) and quartzite (p = 0.01052), test cores (T) and basalt (p = 1.107e-15), and unifacial linear specimens (UF) and vesicular basalt (p = 2.549e-05) (**Fig H in** S1 **File**).

When dimensional variables are studied by reduction model in the handheld cores (**Table D in** S1 **File**), statistically significant differences arise in length (Kruskal-Wallis rank sum test, p-value = 0.0036), width (p-value = 0.016), and mass (p-value = 0.0316). To further explore the relationship between metric variables and core types, two cluster analyses have been performed. The first one arranges core type by volume (LxBxT), showing two main groups. The larger specimens correspond always to reduction models dominated by volcanic blanks. Among them, those characterized by a lower core productivity (i.e. T and UL) are the largest. The second cluster, where quartzite is more abundant or dominant, groups exhausted specimens and bifacial centripetal as the smallest cores in the sample (**Fig I in** S1 **File**). Another second cluster analysis uses mass as the discriminatory variable. Results for freehand cores are very similar, showing that volcanic cores are always the heaviest (in this case, dominated by BA and MM specimens) (**Fig J in** S1 **File**).

The study of negative scars on cores has allowed us to investigate both core productivity and negative scar dimensions by reduction model (**Table 3**). The first variable accounts for the number of negative scars counted per core. We have counted 772 negative scars on 148 handheld cores (90.79% of the core sample). When negative scars are sorted by rock type (433 in basalt, 152 in phonolite, and 141 in quartzite), it appears that the most productive raw material is phonolite (7.7 mean negative scars per core). When core productivity is analyzed by reduction model, a clear progressive increase is observed from the simpler models (T, 1.86 and UL, 4.19) to the more complex reduction strategies (MM, 10.11 and BC,12).

**Table 3:** Core productivity and mean negative scar dimensions by reduction model and raw material type.

Type	Core productivity		Negative scars			
	Core sample (n)	Mean neg./core	Mean length (mm)	Mean breadth (mm)	Core sample (n)	N negatives
By reduction model						
T	52	1.86	33.25	37.81	46	60
UL	26	4.19	31.61	33.48	23	39
UO	4	4.5	29.16	38.33	3	6
BL	23	5.56	36.6	38	22	38
BA	5	6.6	28.41	34.58	5	12
BO	4	8.92	29.12	31.67	13	31
BC	2	12	24.4	29.2	2	5
M	18	10.11	31.76	33.76	11	25
By raw material						
Basalt	102	4.23	34.13	37.12	93	151
Phonolite	20	7.7	32.7	33.14	15	35
Quartz	21	6	26.1	28.96	17	30
Total	148				125	216

T = Test/unorganized; UL = Unifacial lineal; UO = Unifacial ortogonal; BL = Bifacial lineal; BA = bifacial alternating; BO = Bifacial orthogonal; BC = Bifacial centripetal; M = Multifacial.

We have also measured mean length and breadth in negative scars by raw material and by core type. In the first case, there are significant differences in both length (Kruskal-Wallis rank sum test, p-value = <0.001) and width (p-value = <0.001), showing that flakes detached from volcanic rocks are larger and narrower, while quartzite specimens tend to be shorter and wider (**Fig K in** S1 **File**). A Manova test, using the Wilks equation, shows that those differences are mostly due to negative scars produced on basalt. While no significant differences can be observed between quartzite and phonolite negative scars (p = 0.171), there are significant differences between basalt and quartz (p = 0.001) and between basalt and phonolite (p = 0.007). In sum, it appears that flakes detached from basalt cores tend to be longer and, thus, more invasive. When specimens are sorted by reduction model, although differences arise again in both length (Kruskal-Wallis, p = 0.004) and width (p = 0.035) variables, a pairwise Manova test shows that the distribution of negative scars tends to be similar among all reduction models. Despite some significant differences, mostly involving specimens detached form bifacial linear cores (BL/BA, BL/BC, BL/BO, BL/UL, and BO/T) (**Table E in** S1 **File**), these differences are not statistically strong.

A group of 27 handheld cores (16.56% of the core sample) show signs of percussion, evidencing that these pieces were involved in both percussion and knapping processes (**Figs 4.1, 4.2, 5.1** and **5.4**). All these specimens are volcanic rocks (regular basalt, n = 20 and phonolite, n = 7). When cores are sorted by reduction model, percussion damage is preferentially present on BS and US specimens (**Fig L in** S1 **File**). Contrary to what might be expected owing to their low reduction intensity or to their morphology, respectively, T and MM cores with signs of percussion are not particularly abundant.

We have identified 14 cores knapped on tabular quartzite slabs and fragments by bipolar technique (7.9% of the core sample). There is a large statistical overlap between this reduction technique and quartzite (p = 8.9453–07) (**Fig H in** S1 **File**). According to our own analytic criteria [13, 84], most of the specimens showing signs of bipolar load application maintain a stable relationship between platform and base during the reduction process, with linear (n = 6) or semicircular/circular reduction patterns (**Fig 3.4**). Although the sample is meager, bipolar specimens tend to be smaller, but not particularly lighter than most of the handheld core groups (**Figs I and J in** S1 **File)**.

#### Detached material

This category includes complete and broken handheld flakes, as well as a small number of quartzite bipolar flakes. Regarding handheld specimens, most of them (n = 288, 79.52%) have been detached from quartzite cores. Broken flakes are more abundant among quartzite (36.19% of the quartzite flake sample) than they are among volcanic rocks (20.28%), which agrees with the crystallographic characteristics and the highly heterogeneous response to fracture observed in Olduvai quartzites [14]. In fact, most of the documented flake snapping is longitudinal (70.47%), a common accident in Naibor Soit materials, followed by distal (14.28%), proximal (8.5%) or lateral snaps (6.6%). In volcanic rocks longitudinal and distal accidents occur in similar numbers. The relatively common presence of distal snapping in flakes is somehow in accordance with the large number of volcanic cores (47%) showing negative scars with step termination. This trait could be indicative of knapping failures or shortcomings, particularly linked with the process of volcanic rock exploitation. The flakes detached at DS are best defined as small (59.47% are ≤35 mm). Despite their low frequency, larger pieces (46–76 mm) are slightly more common in volcanic rocks (**Fig M in** S1 **File**). There is significant length (Kruskal-Wallis rank sum test, p = 0.001), breadth (p = <0.001), and mass (p = <0.001) difference between volcanic and quartzite flakes. The former are larger and heavier than the latter (**Table F in** S1 **File**).

Regarding their position in the reduction sequence [110], a clear majority of flakes are located in the final stages of the reduction sequence, as most of the specimens are devoid of cortex in dorsal areas and butts (Type 6) or retain partial cortex on the upper face (Type 5). However, first generation flakes (Type 1) constitute, especially in the case of volcanic rocks, the third most abundant group (**Fig N in** S1 **File**). With regard to dorsal areas, the most conspicuous dorsal pattern is represented by two unorganized directions, followed by one direction arrangements (linear or pseudo-linear) and orthogonal patterns. It is significant that orthogonal crossings constitute the most representative dorsal pattern in volcanic specimens (**Fig 6.10**). Flakes with dorsal centripetal arrangements have been produced exclusively in quartzite (**Fig 6.7** and **6.8**), even though centripetal reduction patterns have been performed on both quartzite and basalt (**Fig O in** S1 **File**). Striking platforms are preferentially uni-faceted or plain butts, followed at great distance by cortical and linear types. More elaborate platform preparation is negligible in the flake sample (**Fig P in** S1 **File**). When the three technical traits (flake type, dorsal pattern and platform type) are analyzed by raw material type (volcanic versus quartzite), a two-proportions z test shows that six variables exhibit significant differences between the raw material groups. These variables, from the most to the less significant, are TT6, TT1, TT5, CP UFP, DP2) (**Table G in** S1 **File**). Thus, volcanic flakes are best characterized by Type 1 flakes, Type 5 flakes, and cortical striking platforms, while quartzite flakes are best represented by Type 6 flakes, uni-faceted or plain butts, and unorganized two-direction dorsal pattern.

**Fig 6:**
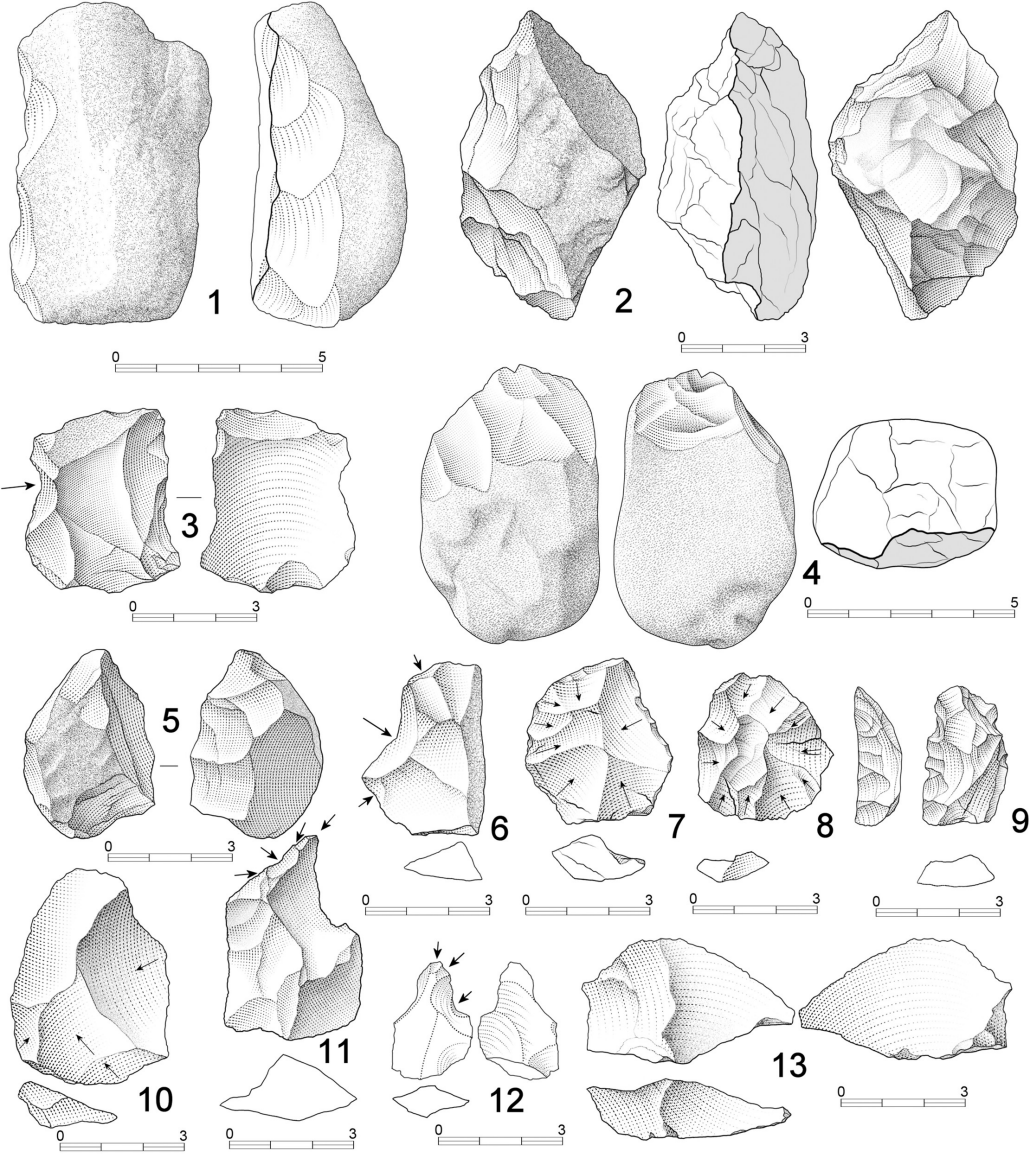
Lithic artefacts from DS. 1. Chopper on basalt showing a lateral, abrupt and continuous series of short removals (74x52x 39 mm); 2. Chopper on basalt showing lateral, bifacial and continuous short removals (71x48x34 mm); 3. Retouched phonolite flake showing a notch on the left side and discontinuous, deep retouch on the right side (43x43x33 mm); 4. Chopper on phonolite, showing bifacial, distal short removals (67x45x38 mm); 5. Quartzite scraper, with continuous and simple retouch on the left side of the ventral surface; 6. Basalt flake with a notch on its left side; 7 and 8. Quartzite flakes showing dorsal centripetal patterns; 9. Abrupt retouch shapes a left-side denticulate on a quartzite positive; 10. Quartzite flake showing a dorsal orthogonal pattern; 11. Borer on basalt; 12. Borer on quartzite; 13. Basalt core rejuvenation flake.

There is an absolute imbalance in the core and flake ratio at DS. Considering the number of negative scars counted in cores and the number of flakes (complete, broken and retouched) actually recovered, 51% of the theoretically expected flakes (assuming that all cores represent knapping processes undertaken in-site) are missing (**Table 4**). This discrepancy is particularly evident when raw material is considered in the equation. The flake recovery rate (FRR) shows that this deficit accounts mostly for volcanic materials. Furthermore, the observed core productivity in phonolite and basalt (**Table 3**) does not accord with the flake to core ratio. The reverse is true for quartzite. To further analyze this puzzle, metric variables of the complete flake sample (n = 227) and the negative scars recorded from the core sample (n = 215) have also been compared. There is a significant correlation between length and breadth in flakes (rho = 10.19, p = 2.2e16) and negatives (rho = 9.52, p = 2.2e16). While both samples show similar length values (Wilcox p = 0.3464, ANOVA p = 0.9032), there are differences in breadth (Wilcox p = 6.35e-10, ANOVA p = 2.07e-07). Thus, negative scars tend to be wider than actual flakes (**Fig Q in** S1 **File, Table H in** S1 **File**). When both samples are compared by raw material (volcanic versus quartzite), a pairwise MANOVA test indicates that there are also significant differences (FQ/NQ p = 0.004 and FV/NV p = 0.002). Thus, in both raw material groups the sample of flakes unearthed at DS is larger and wider than the negative scar sample (**Fig R in** S1 **File**).

**Table 4:** Flake ratios in DS.

A. Flakes	Basalt	Phonolite	Quatzite	Total
Expected (scars/cores)	433	152	141	771
Observed (detached)	61	18	298	377
B. Ratios				
Flake recovery rate	14.08	11.84	211.34	48.89
Flake to core ratio	0.56	0.9	8.76	2.32
Retouch to flake ratio	0.14	0.36	0.17	0.17

A. Number of expected (negative scars on cores) and observed (whole plain flakes, flake fragments and retouched flakes) flakes; B. Flake recovery rate (FRR): percentage of observed flakes with respect to total negative scars counted [111]; Flake to core ratio: Number of flakes (plain, fragmented and retouched flakes) divided by number of cores; Retouch to flake ratio: retouched flakes divided by plain whole flakes).

#### Retouched flakes

Retouched flakes are preferentially made on quartzite (**Table 1**). When metric and mass values in plain and retouched flakes are compared as a whole, there are significant differences in length (Kruskal-Wallis rank sum test, p = 0.0057), breadth (p = 0.035), thickness (p = <0.001) and weight (p = <0.001). In general, retouched flakes are larger and heavier than plain flakes. Most of these differences are maintained when both sets of detached products are sorted by raw material (**Table F in** S1 **File**). Technical traits observed on retouched flakes tend to replicate those recorded for plain flakes. Thus, most specimens in this category are non-cortical (Type 6) or retain some cortex on dorsal areas (Type 5). However, unlike the case of plain flakes, first generation retouched flakes tend to show non-cortical butts (Type 4). Dorsal patterns include one direction or linear (59%), two unorganized directions (23%), opposed (12%) and orthogonal (6%) arrangements. The bulk of butts are uni-faceted (90%), although cortical, broken and removed types are also represented. **Fig S in** S1 **File** shows the percentage distribution of retouch types. A high percentage of specimens included in this category (47.5%) is made up of casually transformed pieces. These pieces tend to show unpatterned, mostly unifacial and discontinuous retouch. The most abundant normative types include notches (simple and double) (**Fig 6.3** and **6.6**) and denticulates (**Fig 6.9**). A few scrapers (where retouch tends to be unifacial, marginal, continuous and simple -35/55°-) (**Fig 6.5**), borers (**Fig 6.11** and **6.12**), and a backed specimen have also been found.

#### Chopper-cores

Four specimens have been included in the chopper-core category (Table 1). They are preferentially volcanic (**Fig 6.1, 6.2** and **6.4**). Remarkably, they are much smaller than flake cores. Mean length is 75 mm (range 67–88), breadth 52 mm (45–63), thickness 39 mm (34–45), and mass 188 g (130–273). Volcanic blanks are oval (n = 2) or hemispheric (n = 1) cobbles. The quartzite specimen was knapped on a thin and flat slab. Unifacially (n = 2) and bifacially (n = 2) worked areas show cutting edges that range between 46 and 50 mm in length.

#### Waste

This category consists of all the by-products and residues produced in the course of knapping tasks undertaken on-site. We have identified, in this order of relevance, debris (flakes and chips ≤25 mm), flake fragments, shatter (blocky/angular undetermined fragments) and core fragments with negative scars whose patterning cannot be clearly identified (**Table 1**). The most striking trait of the waste category is the absolute predominance of quartzite (90.17%) over volcanic rocks (8.54%). This raw material imbalance replicates those seen in cores and flakes, and might be intimately linked to them. Due to anisotropic response to fracture, Naibor Soit quartzite tends to produce a high frequency of residues [14]. Quartzite fragments, shatter, and debris may account for an intensified reduction of quartzite blanks to a point of exhaustion. However, considering the large amount of volcanic rocks involved in both percussive and knapping processes, volcanic residues (in line with volcanic flakes) are again under-represented.

### Intra-site lithic spatial analysis

#### Clustered pattern

The chi-square test based on quadrat counts and the Hopkins-Skellam test both yielded very significant results (p-values < 2.2e-16) confirming that the point pattern is inhomogeneous, and therefore not completely random. The rest of the analysis therefore chooses methods that account for inhomogeneity.

**Fig 7:**
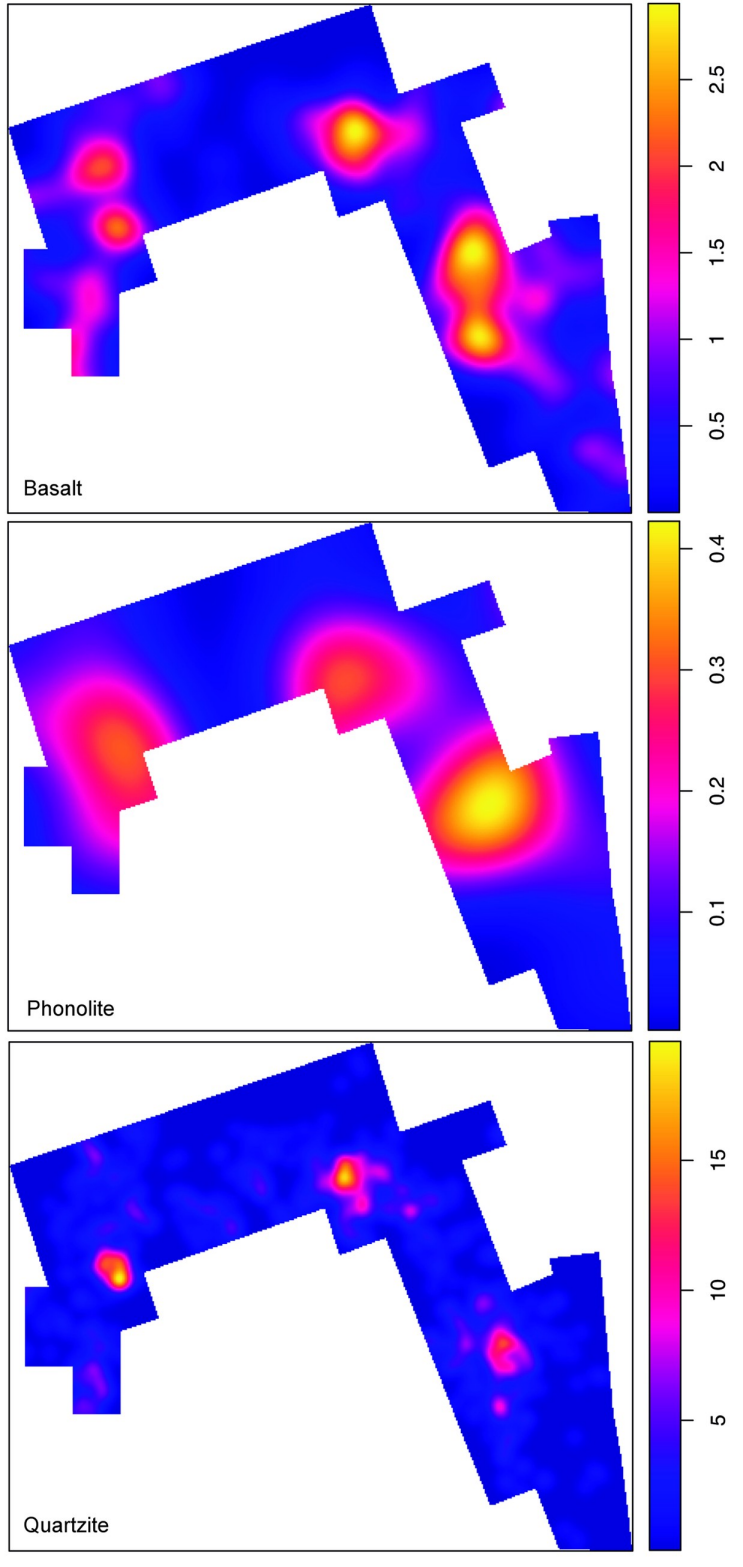
Lithic density maps by main raw materials. Note that the distribution of lithics by the predominant raw material groups accommodate to the clustered pattern in three main high-density areas.

**Fig 8:**
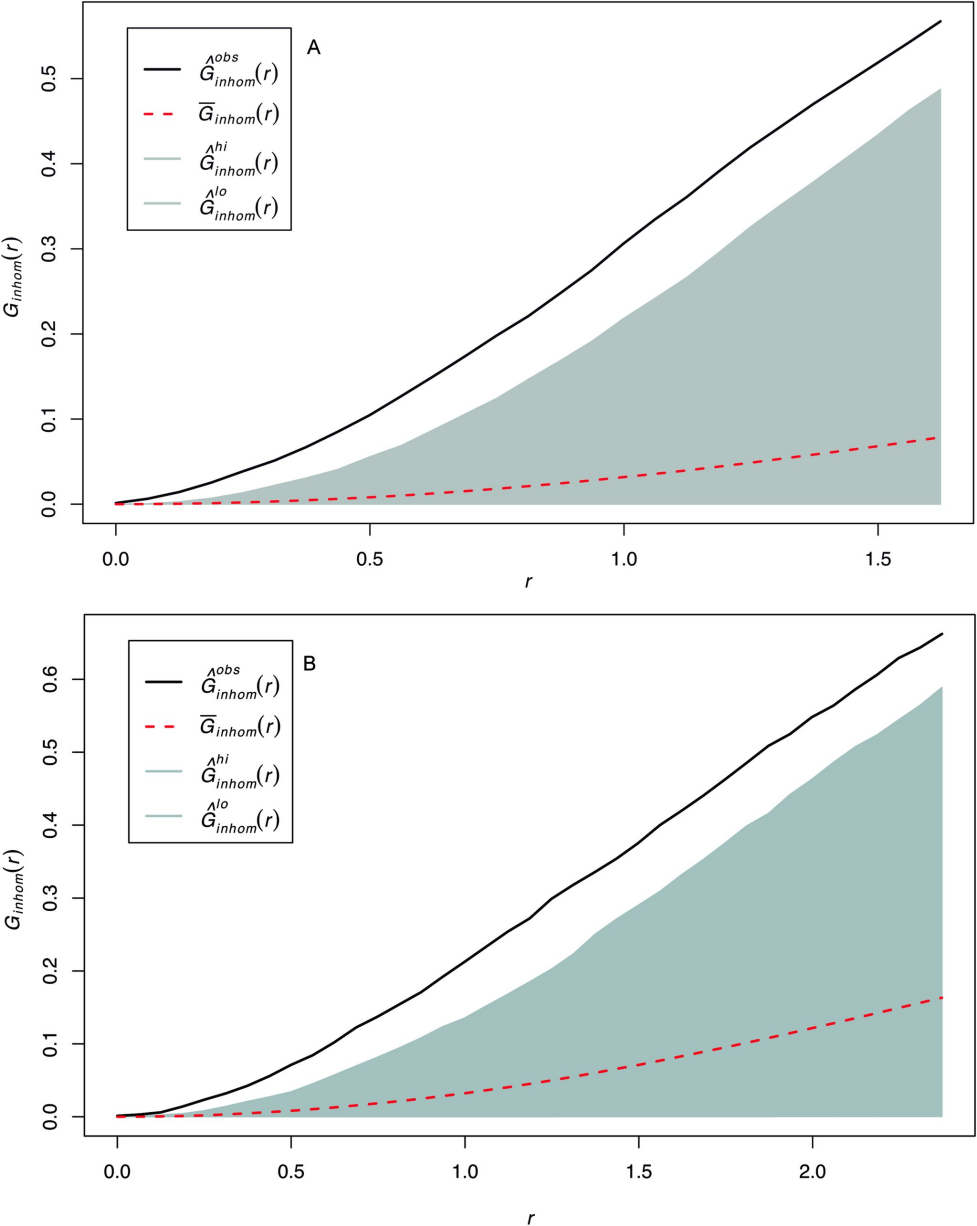
Inhomogeneous G near-neighbor plots. A. quartzite point pattern. B. basalt point pattern. Key: The dotted red line shows the theoretical inhomogeneous Poisson Process and the gray band the confidence envelope. The black lines show the point process of the targeted point processes. Both point processes fall above the Poisson line, which indicates a clustering trend.

The distribution of lithic specimens within the opened window shows the existence of three high-density clusters. Quartzite is the most abundant rock type in the majority of the excavated window, as its mean intensity almost triples the intensity of volcanic rocks. The distribution of lithic implements by the predominant raw material groups (quartzite, basalt and phonolite) accommodate to this clustered pattern (**Fig 7**). The inhomogeneous version of the G-function shows that both the quartzite and the basalt spatial point patterns are clustered, because the observed line appears above the expected theoretical curve depicting an inhomogeneous Poisson process calculated with the intensity function of the spatial pattern in question. The fact that the curve falls outside the envelopes created through Monte Carlo simulations, makes this observation statistically significant (**Fig 8**). In fact, in both cases, the highest density accumulations overlap in the same three cluster areas identified. However, quartzite density peaks are much more intense, as can be observed in the hot spot maps of **Fig T in** S1 **File**, where clusters of quartzite lithics show a much higher probability density per square meter than basalt. Quartzite high intensity areas are statistically significant at r = 1.7 m, while basalt clusters are larger and more diffuse (r = 5m).

**Fig 9:**
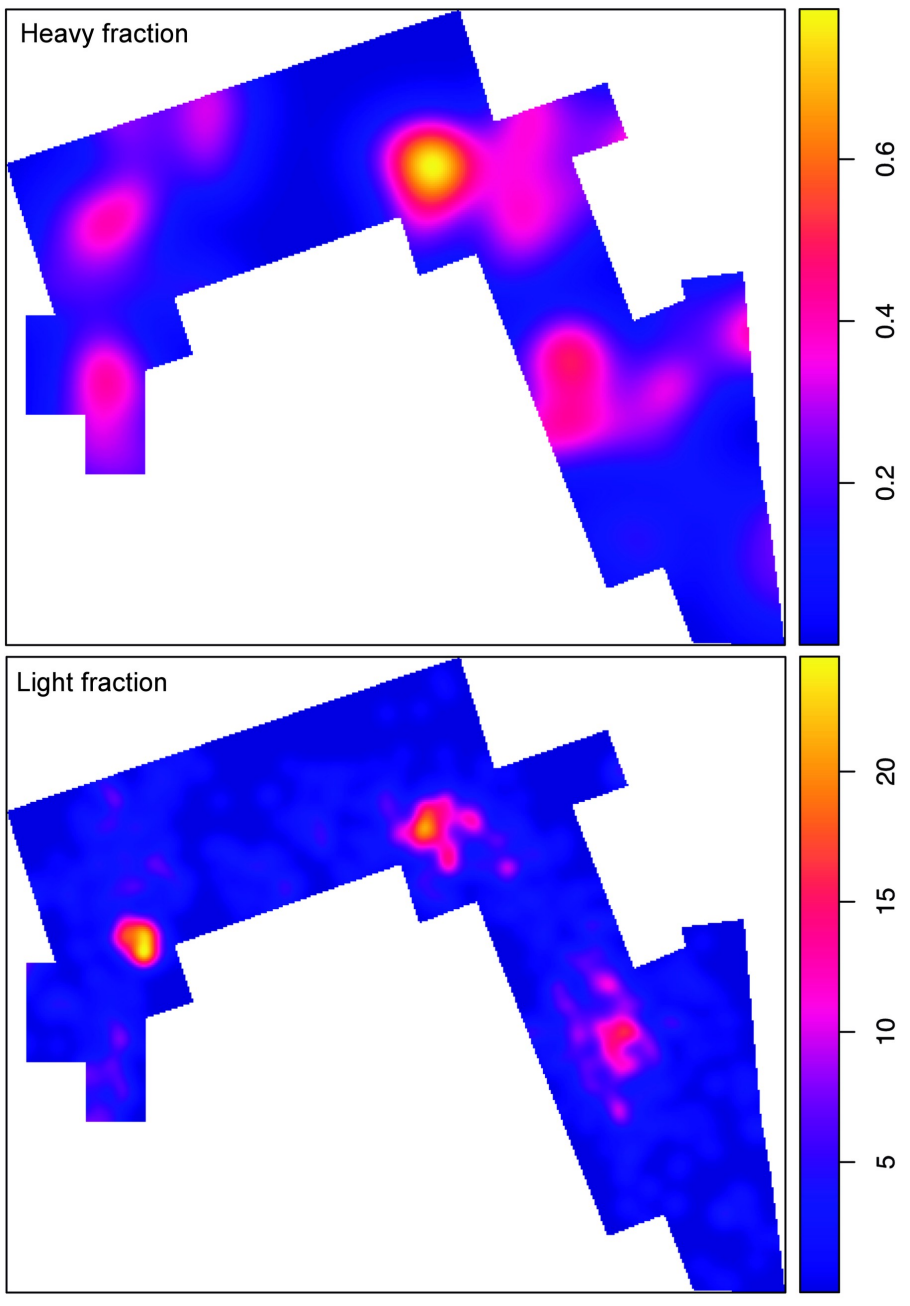
Density maps of heavy-duty and light-duty categories. Heavy fraction: unmodified cobbles, percussion elements, cores. Light fraction: detached materials and waste. Heavy lithics range from 401 to 3894 g (mean = 760 g). Light categories range from 1 to 400 g (mean = 57g).

In agreement with the technological analysis, raw material and mass also show a significant spatial correlation**. Fig U in** S1 **File** results from subtracting the density map of the lighter lithics from the density map of the quartzite lithics and quantifies the differences between both spatial distributions spatially. The legend shows that such differences are small, thus indicating that the distributions of light and quartzite lithics are almost equivalent, and most of the point pattern is eliminated when the maps are subtracted from each other. Similarly, **Fig V in** S1 **File** shows the areas where heavier lithics do not match the distribution of basalt lithics. The distributions of lighter lithics and detached specimens, as well as of heavier materials with nodular pieces also bear resemblances. Even though materials appear relatively mixed, the two groups of lithic materials show different spatial tendencies. Lighter items (represented by those categories dominated by quartzite, such as detached materials and waste) clearly accumulate in the three high density clusters, whereas the heaviest materials (all the categories dominated by volcanic rocks, this is to say, unmodified cobbles, percussion elements and cores) predominate towards the window edges, which are almost devoid of light items (**Fig 9**). This is also reflected in **Figs W-Z in** S1 **File**, which show the spatially varying type probability of lithics according to their raw material, weight and lithic category respectively. The areas demarcated by the white lines are the regions where the estimated probability of a given type is significantly higher than the average proportion.

#### Unmodified materials are not randomly distributed

**Fig 10:**
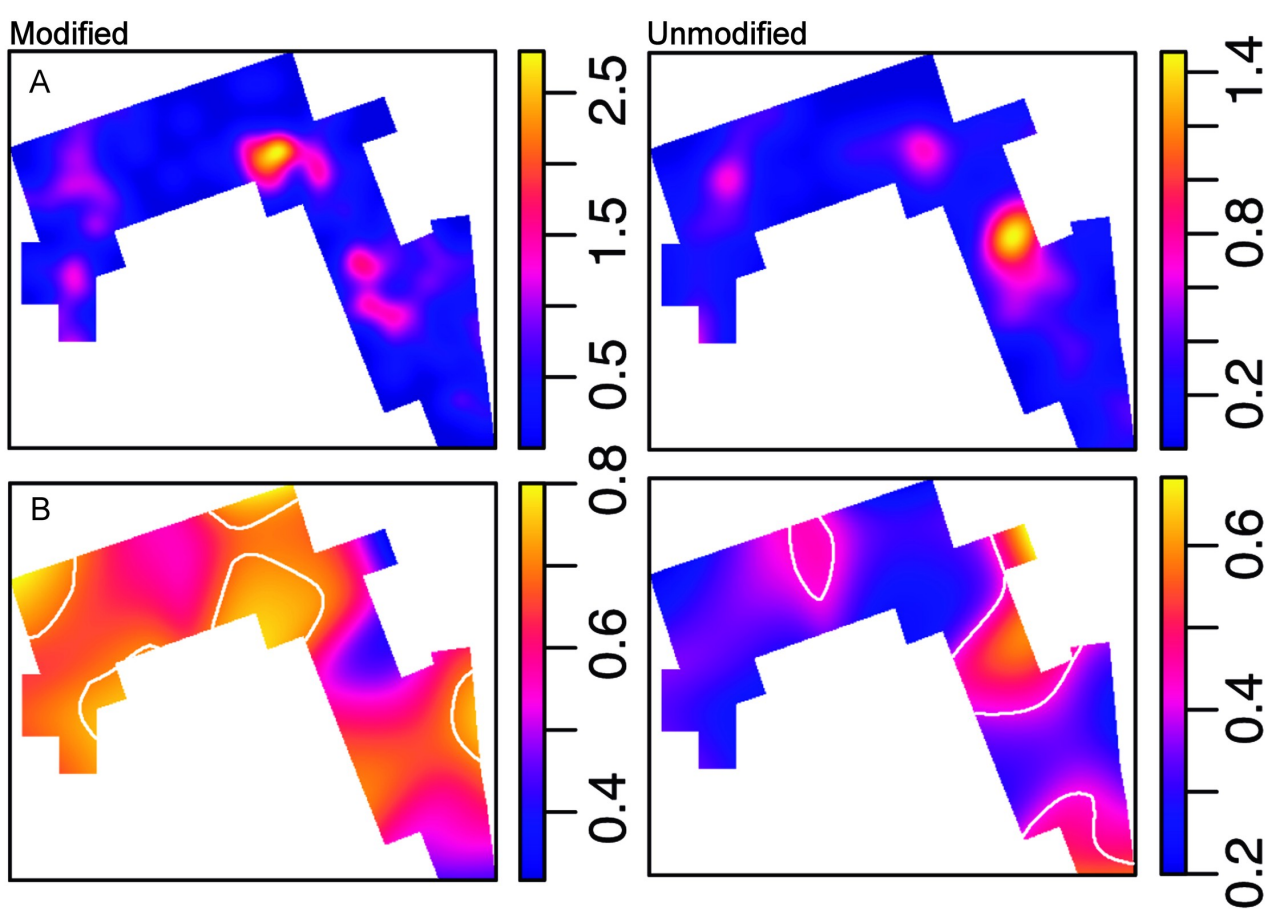
Distribution of modified and unmodified materials. A. Density maps. B. Spatial probability (relative-risk) maps. Tolerance contours (white) show areas of significant deviation from the average proportion between modified and unmodified materials.

A significant outcome of this study is constituted by the inhomogeneous distribution of light-duty (detached pieces and waste) versus heavy-duty categories. A closer look at the spatial distribution of the three heavy-duty lithic categories (unmodified material, percussion elements/hammerstones, and cores) shows that they do not overlap and that they follow different spatial patterns. In order to further investigate this pattern, we lumped the modified materials in a group and established a comparison between the unmodified and the modified materials (**Fig 10A**). **Fig 10B** shows that there are patches where modified materials predominate and other patches where unmodified materials prevail. In these areas, the probability of the type in question is significantly higher than the average proportion. This suggests that the spatial patterns are segregated, and that the spatial distribution of unmodified materials differs from that of hammerstones and cores (**Fig 10**). This observation is confirmed by both a Monte Carlo test of spatial segregation, which yields significant results (T = 0.05, p-value = 0.05), and by the nearest neighbor equality function, which shows that the cumulative proportion of neighbors of the same type is higher than expected if the two spatial distributions were randomly mixed (**Fig AA in** S1 **File**). In sum, the unmodified material highest intensity focal point does not match spatially any other modified lithic category.

#### Percussion and knapping activities are segregated

**Fig 11:**
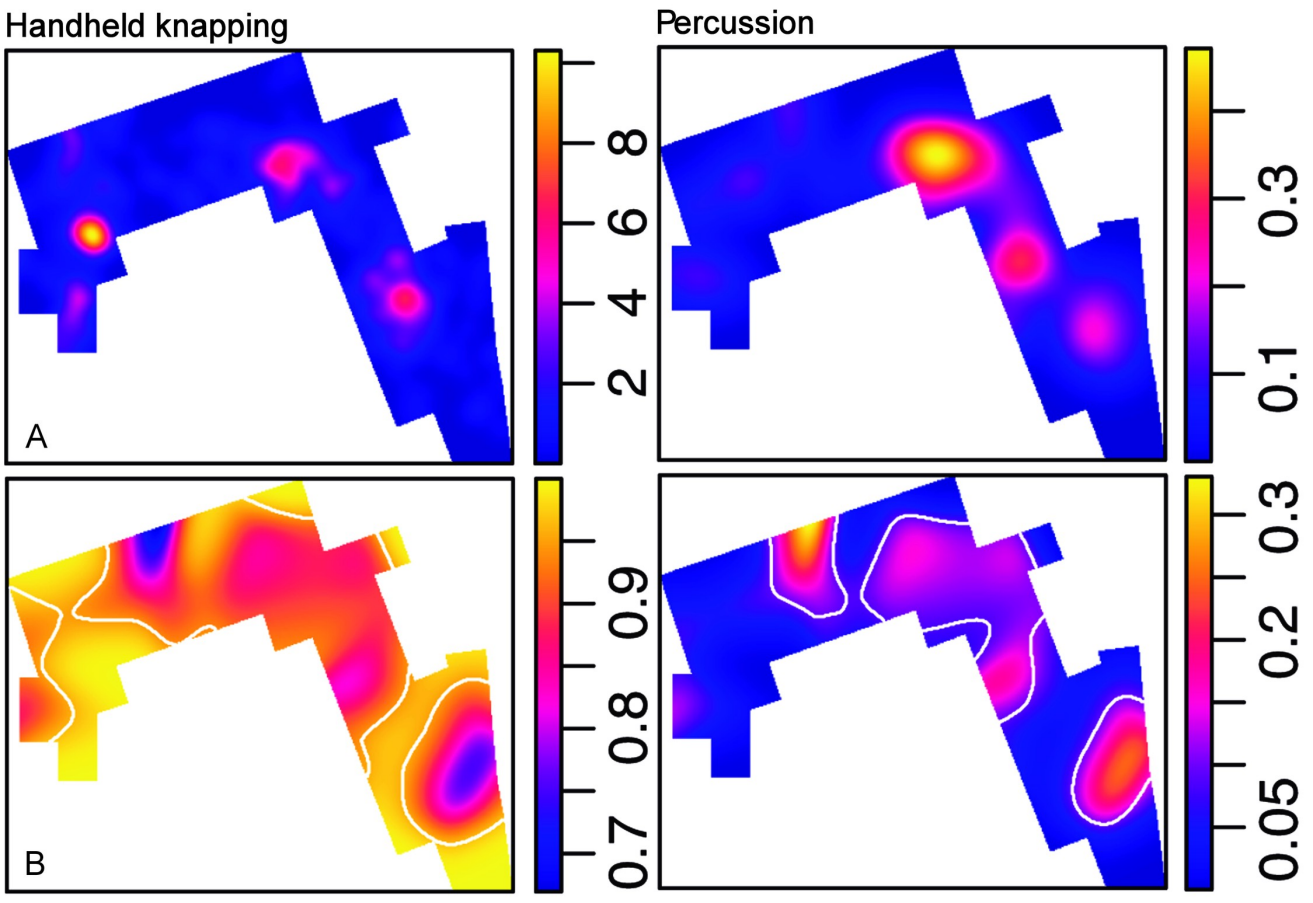
Handheld knapping and percussion. A. Density maps of hand-held knapping and percussion-related materials. B. Spatial probability (relative-risk) maps of both types of materials. Tolerance contours (white) show areas of significant deviation from the average proportion between hand-held knapping and percussion activities.

**Fig 12:**
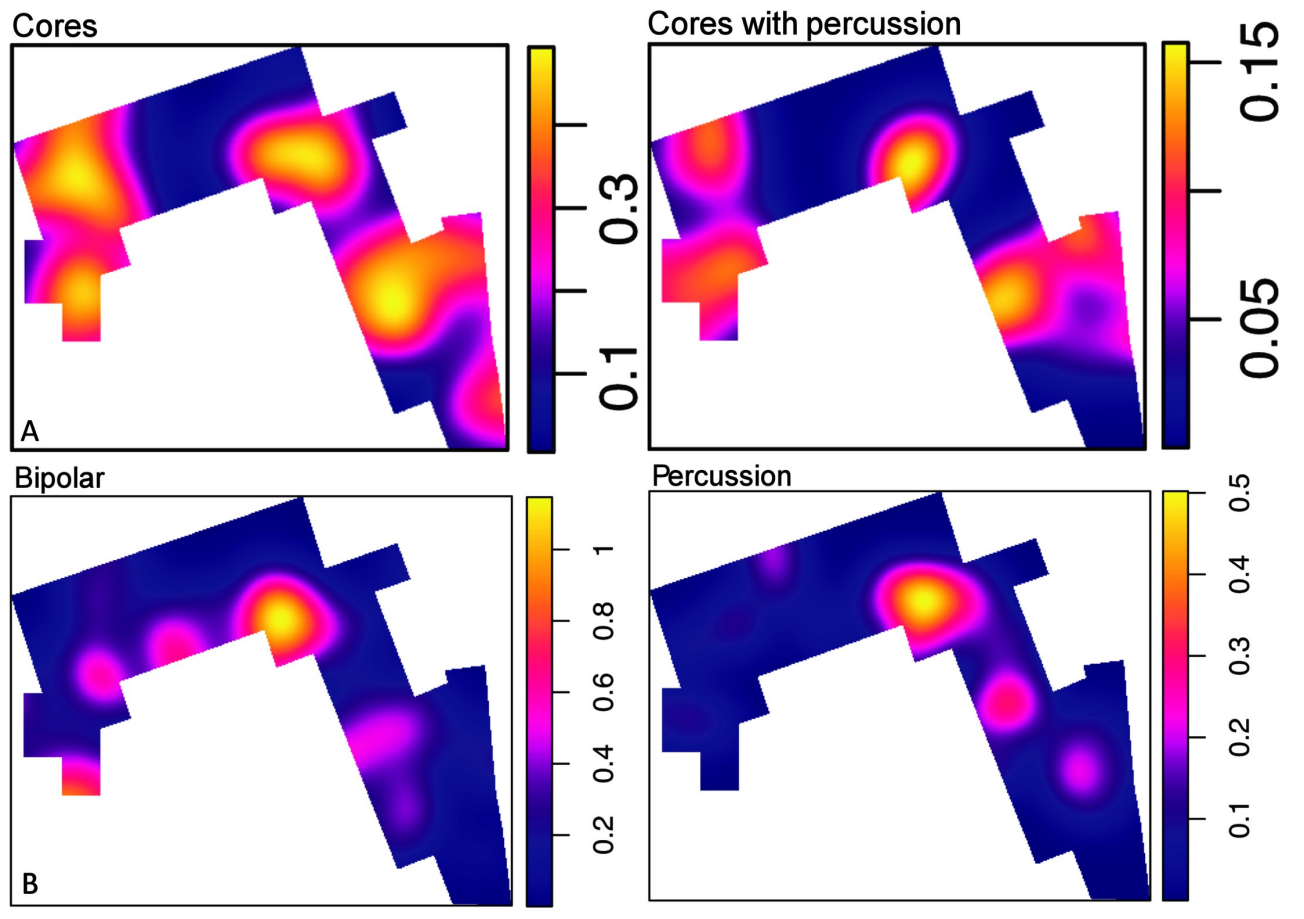
Knapping technique and percussion. A. Density maps of cores and cores with percussion damage. B. Bipolar specimens (cores and identified flakes) and percussion elements (including MBB).

As percussion and handheld knapping activities represent two consistent behavioral signals at DS, their respective spatial patterns have been compared. Percussion elements include all the specimens listed in this category, while in handheld knapping we have considered handheld cores, flakes, broken flakes and retouched flakes. The distribution of both groups within the spatial window and the density maps show that handheld knapping preferentially occurs in the three identified clusters, while percussion is more abundant on the right-hand side of the window and, particularly, in the central cluster (**Fig 11A**). A segregation test shows a significant result (T = 0.37, p = 0.05), which means that both patterns cannot be described with intensity functions proportional to each other. **Fig 11B** highlights the areas where the probability of a type is significantly higher than the average proportion of the two types. The nearest neighbor equality function also shows that points related to both patterns are probably not correlated. **Fig AB(A) in** S1 **File** indicates that, although points of the same type tend to be closer together than would be expected if the point patterns were randomly mixed, this relationship is not statistically significant, because the curve falls inside the significance zone. Interestingly, when handheld cores with percussion damage are compared with the rest of handheld cores (**Fig 12A**), no significant segregation is found (Monte Carlo test of spatial segregation: T = 0.21, p-value = 0.85). This suggests that these cores are probably related to both behaviors, percussion and handheld knapping, and no clear spatial relationship can be statistically determined between the two spatial distributions (**Fig AB(B) in** S1 **File**).

#### Spatial correlation between bipolar knapping and percussion activities

**Fig 13:**
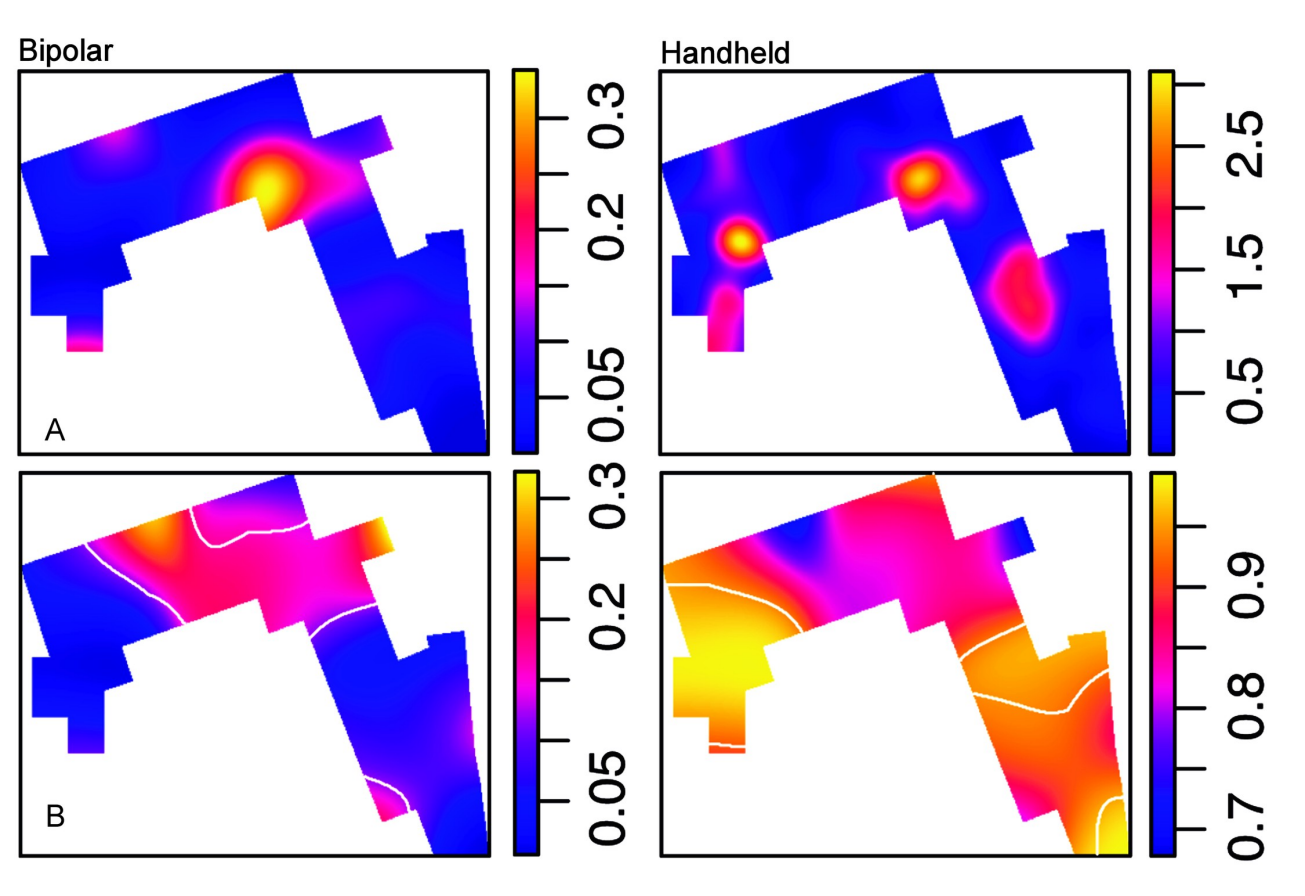
Bipolar and handheld knapping. A. Density maps of bipolar and handheld percussion groups. B. Maps of the relative probability distribution of both groups. Tolerance contours (white) show areas of significant deviation from the average proportion.

The spatial distribution of bipolar material has been compared to both percussion and handheld knapping spatial patterns. For the bipolar/percussion analysis, we have grouped within the bipolar class specimens classified as bipolar cores, bipolar flakes and shatter (blocky and angular fragments that are produced in high numbers in the course of bipolar knapping). All the percussion elements (including MBB) have been taken into consideration for this comparison. Density maps of bipolar and percussion specimens show that both classes overlap in the same high-density spot (**Fig 12B**). **Fig AC in** S1 **File** represents the proportion of percussion neighbor points to a bipolar point of origin, showing that both classes are spatially mixed. A segregation test (T = 0.12, p = 0.65) confirms that both point patterns consistently overlap, sharing a similar structure of spatial inhomogeneity. In other words, the density functions of the point patterns in both classes (bipolar and percussion) are proportional. A similar comparison has been established between bipolar material and handheld material. However, as shatter can be related to both techniques, on this occasion shatter has been removed from the analysis, while bipolar cores and flakes have been compared to freehand cores and flakes. The results show different density and relative probability patterns for each class (**Fig 13**). In sum, the intra-spatial analysis determines that bipolar material and percussion elements show a similar spatial distribution, and that bipolar material and freehand material follow different patterns. This bipolar/percussion spatial overlap may account for a behavioral relationship between both groups.

**Fig 14:**
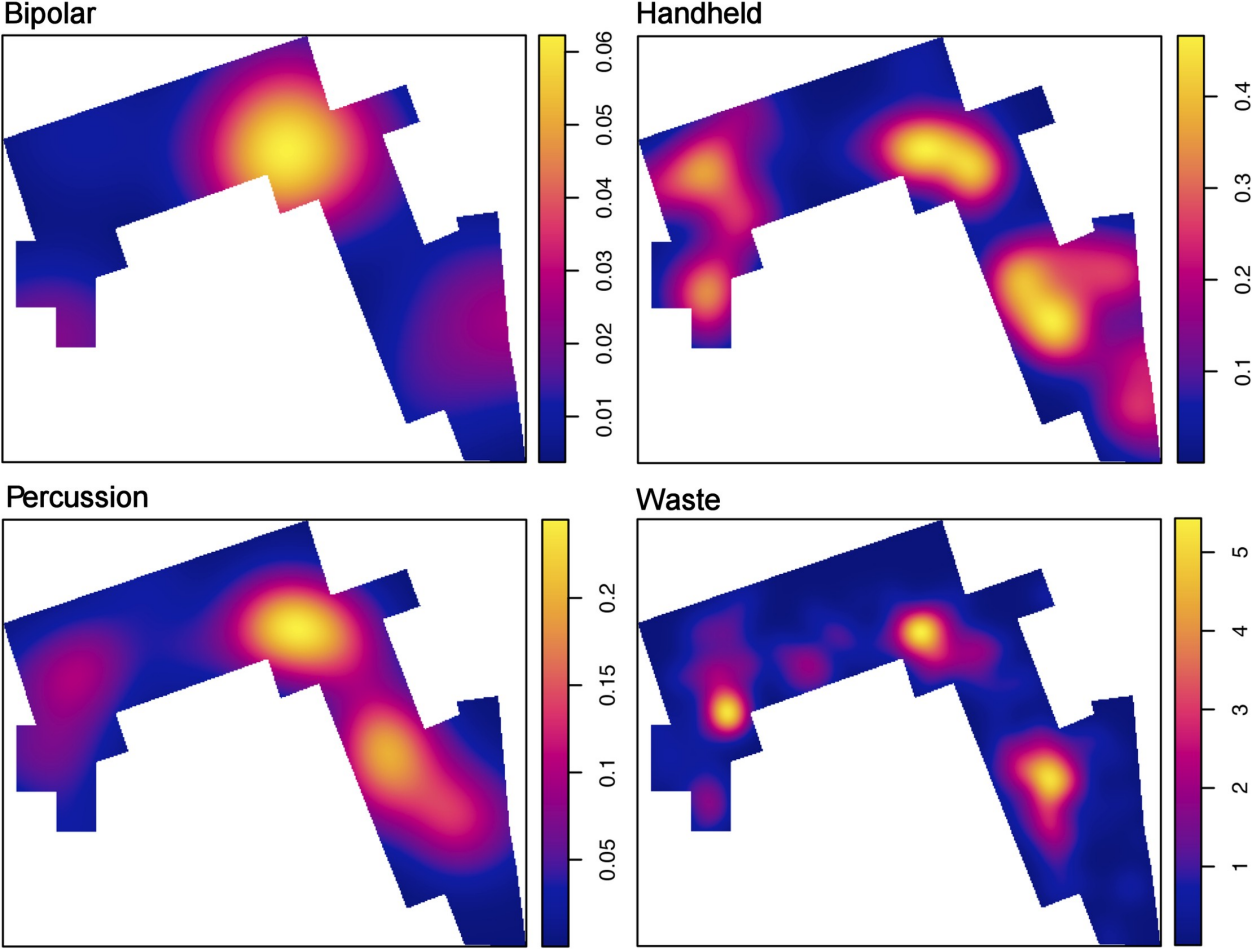
Density maps of bipolar, handheld, percussion and waste materials.

**Fig 15:**
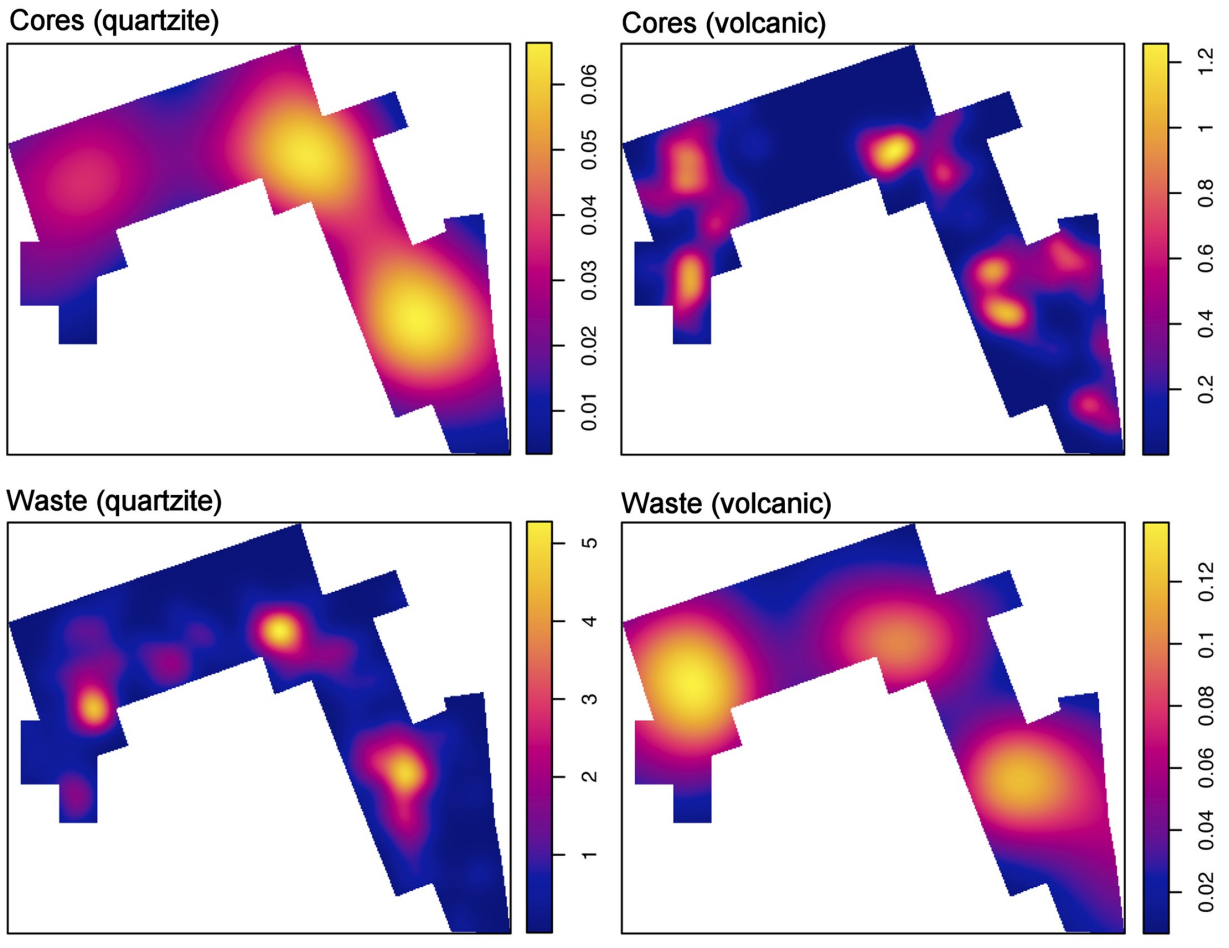
Density maps of freehand cores and by-products (waste) by raw material type.

When including by-products (waste), the density maps show that the intensity of the debris is more consistent with the intensity of handheld material than with that of percussion and bipolar material (**Fig 14**). This could indicate that most residues stem from handheld knapping activities. In fact, when bipolar and percussion materials are left out and the spatial distributions of waste and freehand cores are examined considering raw material type, the spatial relation between cores and waste is even clearer (**Fig 15**). The relative risk maps show that the probability of encountering the two types of materials are similar to the average proportion in all areas of the window (since no area appears delineated by a white line that marks a statistically significant difference) (**Fig AD in** S1 **File**). Finally, the nearest neighbor equality function also shows that the spatial distributions of waste and freehand cores are randomly mixed, i.e. cannot be distinguished statistically (**Fig AE in** S1 **File**). Segregation tests also yielded non-significant p-values for both quartzite and volcanic cores and waste remains.

#### Spatial uniformity of the overall technological pattern between the three high intensive areas

All the p-values of the factor levels in both multinomial regression models were >0.05 and therefore non-significant (**Table I in** S1 **File**), which indicates that the three areas display a fairly similar technological pattern. The proportions between the different raw material types, weight categories, bipolar and percussion materials, modified and unmodified materials, detached and nodular materials, and knapping and percussion materials are statistically indistinguishable between the three areas associated with the three lithics clusters. This implies that all three high intensity areas formed as the result of the same tool use and manufacture activities by hominins and that none of the areas stand out particularly regarding any of the analyzed variables. These results suggesting spatial uniformity could appear to stand in contrast with the observations of the previous analyses, which indicated that some of these variables were characterized by certain spatial variation. For example, percussion and handheld knapping activities presented different intensity functions and segregated spatial point patterns, and light detached materials showed a different spatial distribution from unmodified cobbles, which are predominantly scattered towards the limits of the site. However, the two results are compatible: the observed spatial variation of these variables is not linked to differences between the three high intensity areas, but pertains mostly to differences between the lithic content of the highest intensity areas and the peripheral scatter areas.

**Fig 16:**
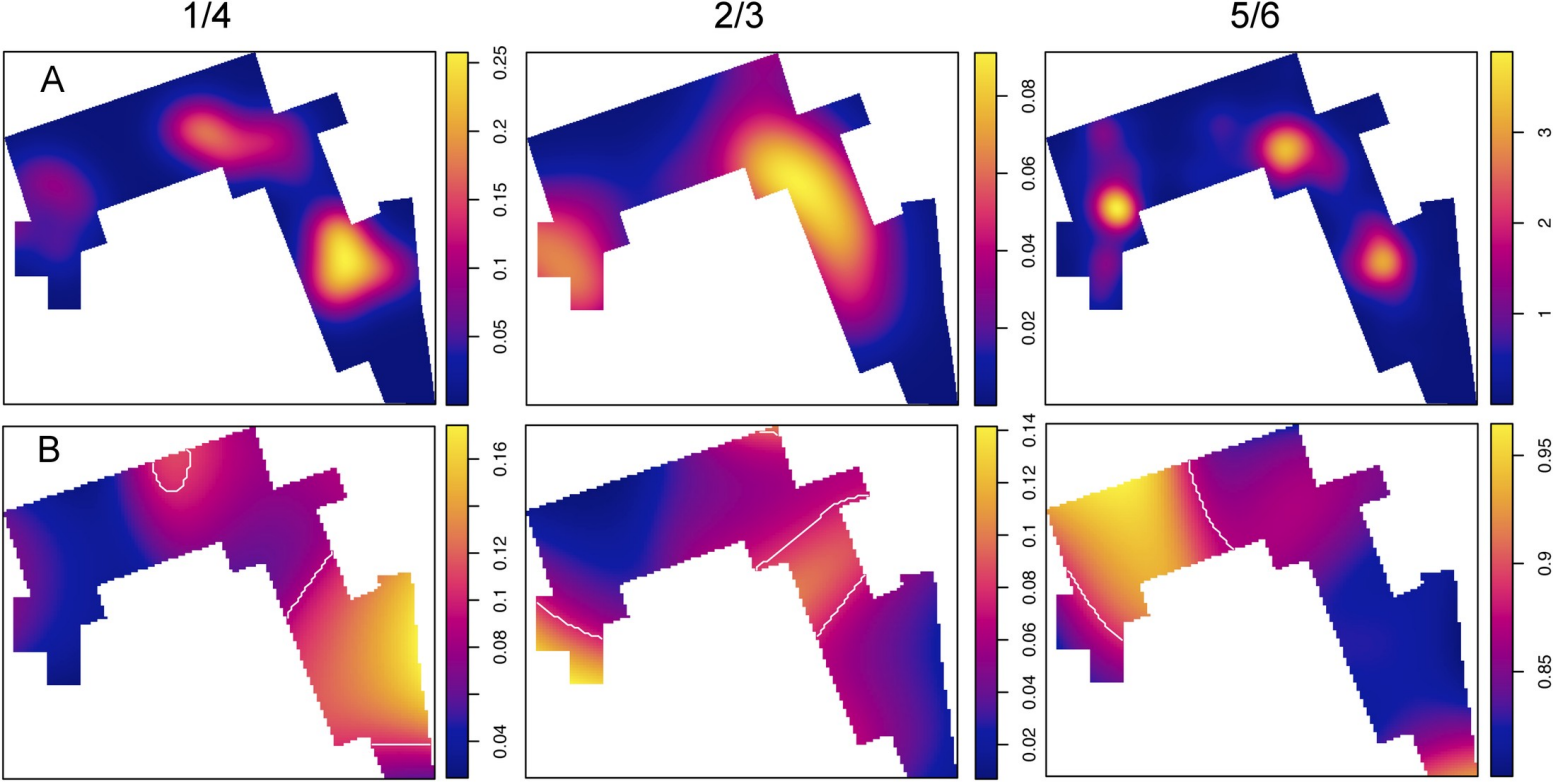
Flake types. A. Density maps of the spatial distribution of flakes according to Toth’s types [110]. B. Spatial probability distribution of flake types Tolerance contours (white) show areas of significant deviation from the average proportion.

There is, however, one interesting exception to this pattern. We examined the distribution of flakes according to Toth’s types [110] and according to the available cutting edge. The results show that the probability of finding flake Types 1 and 4 in Area C is higher than would be expected from the average proportion between the types. Types 5 and 6, in contrast, occur more often than expected in Area A (**Fig 16**). The spatial distribution of flakes according to the available cutting edge is less clear, although flakes with a longer cutting edge (>50 mm) also seem to appear more predominantly than flakes with shorter edges in Area A, whereas the opposite is true between areas A and B-C (**Fig AF in** S1 **File**). The different types of cores, however, do not show the corresponding spatial distribution. Test and unifacial cores predominate around Area A, and more progressive cores, like orthogonal bifacial and multipolar cores concentrate in Areas C and B. The fact that most flakes are quartzite flakes, and most cores are made from volcanic rocks could explain why they show different spatial distributions. However, volcanic flakes of Types 1 and 4 are also more predominant in Area C, and not in Area A, where most volcanic unifacial cores are found (**Fig AG in** S1 **File**).

## Discussion

Due to its large opened area, the exceptional preservation of its faunal and lithic record [49], and the extensive number of archaeological studies based on this site [24, 56, 68, 78, 112–117], FLK 22 constitutes the most influential Early Pleistocene aggregate for research in the archaeology of human origins, representing a referential source for interpretations on a variety of aspects related to early human behavior [78]. The significant accumulation of ungulate carcasses and lithic implements in this particular spot of the lacustrine-alluvial plain points to a node of human activity located in a favored setting (close to a permanent source of fresh water and to abundant tree cover [117]), that can be broadly understood within the framework of a central-place foraging model [118]. Along with the attested processing of faunal resources, the technological behaviors represented in FLK 22 are characterized by the preferential use of volcanic rocks for percussion activities, the predominance of simple exploitation strategies on quartzite slabs (particularly unifacial and bifacial linear methods) to produce small usable flakes, and the extremely high proportion of quartzite waste [119]. A recent assessment of the social network that could underlie the discard patterns preserved at FLK Zinj has suggested that the identified cluster could be the result of the economic activities carried out by a group of 18–28 individuals during a period of 2–4 months [118].

The technological behaviors observed at DS share some relative traits with those recorded in FLK 22 by other authors [119], particularly regarding the preferential involvement of volcanic rocks in percussion tasks (a common pattern in many Bed I and Bed II sites [13, 63, 65, 90], and the overrepresentation of quartzite detached specimens and by-products (including flakes, flake fragments, shatter and debris). In both cases, this absolute overabundance of quartzite detached objects and debris (Table 1) masks the actual predominant role played by volcanic materials in both assemblages. While at FLK 22 the amount of volcanic rocks discarded at the site (measured in kg) clearly exceeds quartzite materials [25, 119], at DS this pattern is even much more significant. Out of the 134.6 kg or rocks introduced here, 84.6% of these supplies is represented by volcanic materials (104.14 kg of basalts and 9.7 kg of phonolites), while quartzite only represents 14.7% (19.75 kg). This observation underlines the preferential and absolute involvement of volcanic raw materials in the formation of the lithic assemblage at DS. Furthermore, at DS volcanic materials show a much better quality and a more intense use than at FLK 22. While unmodified amorphous lavas are dominant in the latter (probably weathered fragments of the local basal lava outcrop, as hypothesized [120], volcanic rocks at DS, including unmodified specimens, are mostly characterized by fine-grained and rounded cobbles, actively involved in both percussion and a wide variety of exploitation processes. Thus, one of the most remarkable technological traits of DS, which marks a substantial difference with the FLK 22 lithic patch, is the preeminent and active role of volcanic cobbles in the percussion (18.15 kg of volcanic hammerstones vs 2.5 kg of quartzites) and, mostly, knapping sequences (54.15 kg of volcanic cores vs 8 kg of quartzite cores) undertaken at the spot. This might be explained by the presence of good-quality volcanic cobble sources available nearby. Furthermore, this specific trait of the local landscape, the purported local availability of good volcanic cobbles, might have influenced the set of technological and economic behaviors displayed at DS. This scenario of an efficient exploitation of local resources (i.e. rocks and game) by hominins at the spot is in accordance with the results of the zooarchaeological analysis, which showed that the majority of represented bovid carcasses belong to local fauna, in particular *Kobus sigmoidalis* (waterbuck) [77].

Regarding rock supplies, a number of relevant conclusions can be drawn from our current knowledge of the local setting. DS was part of a lake margin ecosystem during the *Zinj* paleolandscape. Towards the North and Northwest of the spot, a shallow central lake was present, while towards the South the landscape was represented by a field of basaltic lavas, formed by lava tongues and elongated to rounded lava mounds or tumuli. Perfect preservation of pahoehoe crusts on the basalt shows that they spilled out in rapid succession, almost certainly from a vent nearby rather than from a summit crater of one of the large volcanoes [85]. Coast line morphology in this area is sinuous due to the irregular geometry of the lava field [79]. In more detail, the clay paleosurface of Level 22 is deposited forming an onlap on the irregular, and tilting towards the North, basalt surface. Although this lava outcrop could be the source of some amorphous unmodified specimens retrieved from DS, it is significant that the bulk of volcanic specimens, along with the majority of hammerstones and cores, is represented by rounded cobbles. It seems reasonable, thus, to suggest the local presence of a drainage system, channels or processes of hydric flux, able to transport rounded specimens to the vicinity of DS.

Immature drainage systems developed on basaltic lava fields show a drainage system controlled by the geometry and topography of the flow field itself, with the drainage system developing between flow lobes and in lows of the lava flow field [121]. In present times, also in East Africa (i.e. Afar region in Ethiopia), lava fields control fluvial drainage system development [122] and the creation of isolated mini-highs of basalt within the drainage system. DS is located in a slightly high position, very close (few m) to one of these mini-highs, formed by the interfluve of two rivers (140 m to the West and 150 m to the East). In this position, even the deepest basal areas of these channels that are in contact with the basalt (1–1.6 m) exhibit low energy (formed by pumice, silt and fine-grained sand conglomerates) due to a very low gradient. Occasionally, large basalt irregular blocks are identified, probably transported from the margins or detached from the bottom. Rounded cobbles have not been documented in the local stratigraphic record. Thus, transport and deposition processes of volcanic cobbles during DS times should be found upstream, where topography is more abrupt.

The basalt paleosurface has been measured with a sub-centimetric DGPS in order to calculate gradient variations in the vicinity of DS. Although topography is locally irregular (due to the presence of lava mounds and tumuli higher than 1 m), the basalt gradient towards the South reaches 0.04 m m^-1^. This is a very high value for a fluvial system and is more in agreement with mountain rivers in rock [123, 124]. The gradient of bedrock channels is almost certainly well in excess of those encountered in alluvial channels, which is consistent with the typical coexistence of gravel bed and bedrock reaches [125]. On the other hand, a bedrock river is rarely entirely free from sediment cover [126], and usually encompasses the range of mixed alluvial‐bedrock channel bed conditions [127]. Although bedrock systems are sediment sparse, they are typically extremely efficient [125]. The combination of a high gradient and a rocky riverbed, suggest a favorable scenario for a bedload transport (sand, gravel and cobbles) upstream of DS. Thus, exceptional events of higher runoff could have deposited lava cobbles to the southern area of DS via the drainage system developed on the lava field. In sum, the presence of good quality volcanic rocks in the vicinity of DS might have constituted a powerful attraction for hominins at a local scale and might explain in part the specific selection of this spot within the Zinj landscape.

Although raw material availability might have influenced a number of behaviors linked to tool production and tool flow around the lacustrine landscape, it certainly facilitated other economic activities. An important part of the activities that took place at the site are related to carcass processing and butchering activities. The taphonomic study at DS showed that hominins processed a minimum of 27 mostly complete small and medium-sized ungulate carcasses in a period of less than one or two years. Although carcass butchering is similarly documented spatially in all three high density areas, marrow extraction using hammerstone percussion is observed predominantly in one of these areas (Area B) [77]. This is evident in the relative spatial distribution of percussion marks on bones and to a certain extent also in the distribution of percussion tools.

The impact of the anthropogenic node of DS in the Zinj landscape can be supported by the core/flake ratio documented at this site. Underrepresentation of volcanic flakes and overrepresentation of quartzite flakes are recurrent patterns in Olduvai Bed I and Bed II lithic assemblages [9, 24, 25, 49, 116, 128–131]. A number of authors argue that this is evidence that hominins treated the raw materials differently and that quartzite and volcanic rocks were part of different economic/functional/regional strategies [25, 113]. Regarding the Zinj landscape, deficit of basalt flakes has also been documented in both FLK 22 [119, 131] and DS. However, this imbalance is notably more significant at DS, due to the fact that a more intense and complex role of volcanic knapping has been documented here. Taphonomic studies [77] confidently allow us to rule out natural causes related to water action to explain volcanic flake deficiency at DS. Thus, the most consistent explanations for the observed imbalance between the core sample and the flake sample (i.e. deficit of volcanic flakes and deficit of quartzite cores) must have been behavioral [132] and might be related to core/flake landscape flow (i.e. artifact input and output) [25, 113]. At DS the following facts are true for volcanic lithics: a) flakes are dramatically underrepresented in the studied sample (low FRR and FCR); b) flakes are statistically larger and wider than negative scars observed in volcanic cores; c) flakes are statistically characterized by a significant retention of cortex on dorsal areas and butts; and d) first generation flakes are mostly represented by volcanic rocks. This concurrence of facts would imply that an undetermined number of second and, mostly, third generation volcanic flakes might have been taken off-site by the DS knappers. This curation behavior agrees with experimental studies suggesting that cutting edges produced on volcanic flakes are more durable than chert and quartzite edges [132–134]. Thus, the longer use-life of volcanic edges would favor long-term cutting and processing activities [25] and, thus, would explain the preferential involvement of volcanic flakes in off-site behaviors [130, 132].

Regarding quartzite specimens: a) flakes are overrepresented in the studied sample (very high FRR and moderate FCR); b) flakes are larger and wider than negative scars observed in quartzite cores; and c) flakes are characterized by non-cortical butts and dorsal areas (however, a proper observation of cortical areas in quartzite specimens tends to be underrepresented). In this case, although an opposite landscape flow pattern (flake input) could also be entertained [131], some evidence (predominance of exhausted cores in quartzite, smaller size of quartzite cores, lack of quartzite specimens in the earlier phases of exploitation, and absolute predominance of quartzite waste) may account for a significantly more intensive exploitation of quartzite blanks as the most reasonable explanation for the core deficit documented [24]. This perspective accords with the crystallographic nature of Naibor Soit quartzite and with its heterogeneous response to fracture; factors that accelerate reduction [14]. Furthermore, the more effective sharpness of quartzite cutting edges [134] would favor expedient and short-term on-site activities that also might favor local abandonment of quartzite flakes and, thus, explain local overrepresentation of quartzite materials [25].

Mainly due to the variable statistical significance of the categories identified here, not all the studied spatial combinations considered in this work have rendered significant spatial interdependences. However, our study has unraveled a number of valuable observations that might constitute the preserved spatial imprints of the technological behaviors exhibited by the DS hominins. First, the fact that the spatial distribution pattern of unmodified cobbles does not overlap with the rest of nodular categories (hammerstones and cores) constitutes a significant observation. Unmodified cobbles in Olduvai, labelled as manuports by other researchers [24, 49], have been interpreted as intentional accumulations of rocks as raw material remnants in specific locations of the landscape for future use [15, 24]. Alternative interpretations have concluded that most of the unmodified rocks recovered from Bed I archaeological sites are in fact natural objects, randomly distributed in the landscape and spatially overlapping with stone artifacts, but devoid of any intentional accumulation indicative of anticipation and curation behavior [120]. There are convincing reasons to infer that this might have been the case in FLK 22, where low-quality amorphous lavas are representative of the unmodified cobble sample [119]. In other Bed I cases, however, there is consistent evidence that hominins casually exploited some of these local amorphous specimens randomly distributed on the ancient landscape [90].

From the spatial point of view, the picture that emerges from the distribution of unmodified cobbles at DS is inconsistent with a scenario of randomly distributed volcanic clasts, incorporated in lacustrine-alluvial contexts via a number of natural processes of clastic transport and deposition [135–137]. First, unmodified materials constitute a significant amount of rock mass in the DS assemblage. In all, 37.2 kg of unmodified materials are present in the site (98% of which are made of volcanic rocks), less than the 54 kg of volcanic cores counted, but significantly more than the 18 kg of volcanic percussion tools discarded. Typometry also supports this perspective. Among the 128 unmodified specimens in which the original shape has been recognized (including broken pieces), 52% (n = 67) are classified as spherical/subspherical cobbles, while 30% (n = 39) are amorphous items. Furthermore, among these amorphous pieces, 51% (n = 20) are fine-grained basalts and phonolite. Only 15% of unmodified cobbles documented at DS share the same vesicular structure and irregular shape that predominates among the extremely low-quality unmodified pieces recorded in FLK 22 [110].

The size and mass comparison between unmodified cobbles and hammerstones shows that the categories are statistically alike regarding maximum length. This again rules out the concurrence of natural processes (that would imply a much wider range of clast morphology and size) for the input of unmodified cobbles on-site. In sum, given that at DS: a) unmodified cobbles follow a patterned spatial distribution that does not accord with the spatial distribution of the other nodular volcanic categories, b) that hominins were selecting the heaviest specimens (mass positively correlates with traits such as rock tenacity and homogeneity) among a similar-size population of volcanic nodules to undertake percussion activities, c) vesicular and regular basalts show no significant size and mass differences (in all instances an Anova test shows p = >0.05), and d) that hominins were also undertaking an intense exploitation of a large number of volcanic specimens, it seem reasonable to conclude that this significant concurrence of volcanic cobbles at this spot of the Zinj landscape is due to intentional accumulation in the DS node from nearby sources. In close connection with this raw material supply availability, the spot might have been a relevant focal point for raw material selection and use in a number of activities undertaken on-site (including carcass processing). The behavioral pattern described here is somehow in line with the interpretative framework proposed by Potts [24] in his “stone cache” hypothesis.

Another significant observation of the spatial analysis undertaken in this paper shows that, leaving aside the accumulation of cobbles, the two most conspicuous lithic activities documented at DS, knapping and percussion, are spatially differentiated in the excavated window. This segregated spatial print constitutes an indirect support for the relevance and diversity of those percussion tasks complementary to tool production carried out on-site. Growing evidence points to the central role played by pounding activities in Oldowan contexts [5, 7, 138, 139]. Although researchers are fully aware of the variety of processing tasks and diversity of organic materials involved in this type of behavior [140, 141], little information is available on the way these activities are spatially expressed or framed, both at wider and local scales. Regarding percussion activities, for example, in a wider landscape framework, some authors have argued that percussion tool inter-assemblage variability might indicate differentiated pounding tasks undertaken at specific spots of the landscape [140]. Predominance of percussive over knapping activities at FLK North exemplifies that local resources in specific spots of the Bed I landscape might have been powerful economic attractors to undertake pounding tasks linked to the processing of vegetal tissues [90]. At the site scale, the identification of functional spatial variability is far more difficult, since percussive and knapping activities tend to concur and overlap in archaeological sites. The recurrent presence of cores bearing signs of percussion in many Bed I and Bed II Olduvai sites shows that transfer of cobbles from percussion to exploitation sequences was a common practice. This evidence shows that nodes were responsible for a flexible web of multi-functional patterns that are difficult to segregate. At DS, for instance, when cores with signs of percussion are considered, no specific spatial interdependences with percussion or knapping activities emerge. However, although subtle, our general spatial observations might indicate that different activities were also related with some sort of compartmentalization of the space.

Another interesting outcome of our intra-site analysis is the observation that specimens related to bipolar technique and to percussion display a similar spatial distribution. This is a provocative correlation that might be indicative, in some instances, of a behavioral link between the groups of artifacts. In Olduvai, bipolar technique performed on quartzite slabs seems to have been a common knapping behavior during Bed II times [49, 133, 142]. In Bed I, despite being recognized in some cases [24, 90], the anvil technique appears to be a less common solution for core reduction. In the case of DS, technological signs of the use of the bipolar technique are scarce. The assemblage includes a small group of flakes and cores showing direct signs of bipolar load application [13, 14, 74]. Indirectly, an undetermined portion of quartzite waste can be related to bipolar knapping, if we accept the assertion that high numbers of shatter and chunky fragments are common by-products of bipolar reduction [24, 143–145]. However, our analysis shows that at DS, debris is spatially more consistently linked with handheld material (cores and flakes) than with percussion and bipolar categories. While the lack of connection between waste and percussion activities has already been underlined [13, 90], the conclusions that emerge from our spatial analysis cast doubts on previous assumptions preferentially linking debris production and bipolar technique [143, 144, 146–148]. Once again, the nature of Naibor Soit rocks particularly facilitates the generation of large amounts of debris, whatever the knapping method in use.

An explanation for the presence of the bipolar technique in Olduvai suggests that it is an efficient way to exploit Naibor Soit tabular slabs in search of usable flakes [13, 14]. An alternative hypothesis has proposed that the bipolar technique might have been used to split quartzite slabs in easy-to-handle cuboid fragments that would be subsequently transformed in spherical forms via intense percussion [17]. This alternative perspective, thus, would link bipolar cores with MBB (including spheroids and subspheroids), and therefore bipolar cores with percussion tasks, as different stages of the same functional sequence. The spatial interdependence between bipolar and percussion categories observed in DS (linking the bipolar technique mostly with MBB but also with the general universe of percussion activities) must be taken with caution, because the sample of specimens confidently identified as MBB or bipolar cores/flakes is scanty. However, this spatial observation can warn us about our incomplete understanding of the anvil technique, suggesting that a more complex array of behaviors could be related to it. Although a sequential link between the bipolar technique and percussion might be related in specific cases, the wide variety of sizes and shapes documented in both bipolar cores and MBBs [13, 63, 65, 149] rules out a univocal sequential connection between the anvil technique and MBBs in all instances. In fact, consistent archaeological and ethnographic evidence confirms that the bipolar technique constitutes an efficient core exploitation method widely implemented over time and space [3, 84, 142, 147, 150–154]. Further investigation is needed into the behavioral meaning of the anvil technique in ESA contexts.

Overall, the intra-spatial analysis shows that no discrete lithic activities can be related to the three clusters documented at DS. This observation rules out a conspicuous spatial compartmentation or task specialization in the lithic activities carried out on-site. Similarly, spatial variation between different types of faunal remains was not too marked between the three areas, although there were mild spatial and taphonomic variations between the clusters [77]. Carcass processing took place in all three areas, although the clearest evidence of the complete carcass butchering process (including marrow extraction through hammerstone percussion) is documented in Area B, which could be considered the main accumulation. The spatial analysis of faunal remains showed that Area A is characterized by an overrepresentation of appendicular elements [77]. This area also shows the highest number of flakes of Types 5 and 6, which could be indicating that Area A might have been an extension or secondary refuse area of the main assemblage, where mostly appendicular elements or incomplete carcasses were processed.

Both the lithic and bone point patterns have similar spatial distributions and the highest intensity areas overlap in space, although lithic remains showed even more clustering than bone remains. The spatial analyses of both types of archaeological remains therefore show that hominins congregated in reduced focal spots for repeated carcass consumption, knapping and other tool-related activities. This behavioral pattern could be described as centripetal, as opposed to the centrifugal use of space observed in modern hunter-gatherer camps, where remains appear in multiple less-dense clusters related to the individual household use of camp space [55], and could be indicating a very cohesive group social structure and the existence of a cognitive partitioning of space.

## Conclusions

DS is a multi-cluster site, formed by the inhomogeneous accumulation of abundant lithic implements and faunal remains. Through the combination of a techno-economic study and an intra-site spatial analysis, this work has revealed a number of relevant conclusions. The lithic assemblage is clearly determined by raw material type. Lavas and quartzite are sharply related to different technological behaviors. Volcanic cobbles, basalt and phonolite, were introduced in this particular spot of the lacustrine-alluvial plain in considerable numbers. Along with a significant sample of unmodified rounded cobbles, volcanic materials were intensively used in percussion tasks and, more significantly, in a wide variety of knapping strategies. Cores are abundant and diverse. The most significant exploitation patterns are simple (casual and unifacial or bifacial linear), although orthogonal arrangements or multipolar specimens are also well represented. The accumulation of volcanic cobbles and the intense use of lavas at the spot suggest a close source of suitable volcanic rocks, which were transported to this landscape node. Despite the significance of on-site volcanic rock knapping, the imbalance of volcanic flakes suggests outward fluxes of third generation volcanic flakes. On the contrary, quartzite is best characterized by freehand flakes and waste (including shatter and debris), while freehand quartzite cores are underrepresented. Predominance of quartzite cores in advanced stages of reduction plus the identification of the anvil technique might account for this imbalance, suggesting a very intense level of quartzite exploitation.

The intra-spatial analysis of the horizontal associations between lithic categories shows no differences among the three lithic clusters identified. Artifact classes are similarly represented in each one, showing that similar technological activities were equally undertaken in different areas of the anthropogenic node. A geostatistical analysis of the spatial correlations between lithic categories has revealed three conspicuous traits that might represent the spatial print of the technological behaviors undertaken on-site. First, the spatial distribution of unmodified cobbles is not randomly distributed. These specimens show a distribution pattern that does not overlap with the rest of nodular volcanic categories (hammerstones and cores). This spatial pattern, together with the metric and petrographic characteristics of these cobbles, points toward the accumulation of volcanic supplies in this specific spot of the landscape. Second, the study has shown that the intra-site spatial patterns of percussion elements and freehand knapping activities differ. This could suggest that pounding and knapping tasks might have taken place in different areas of the cluster. Our analysis shows a strong spatial correlation between percussion activities and implements associated with the bipolar technique. This observation might point to a connection between pounding activities and the anvil technique in some instances, suggesting a more diverse functional meaning for the latter.

## Supporting information

S1 File Supplementary tables and figures. (DOCX)
