# Supplementary material for: Tracing the spatial imprint of Oldowan technological behaviors: A view from DS (Bed I, Olduvai Gorge, Tanzania)
Source: PLoS One. 2021 Jul 12;16(7):e0254603. doi: 10.1371/journal.pone.0254603 (PMC8274881; doi:10.1371/journal.pone.0254603)
Supplement: S1 File — (DOCX) [file pone.0254603.s001.docx]

**S1 File. Supplementary tables and figures**

|  | **Variables** | **Factors** |
| --- | --- | --- |
| **Model 1** | Lithic category | Detached material |
|  |  | Nodular material |
|  | Modification | Modified |
|  |  | Unmodified |
|  | Weight/Mass | Light material |
|  |  | Heavy material |
| **Model 2** | Raw naterial | Basalt |
|  |  | Phonolite |
|  |  | Quartzite |
|  | Bipolar technique | Bipolar material |
|  |  | Percussion material |
|  | Handheld technique | Handheld material |
|  |  | Percussion material |

**Table A. Variables.** Variables used in the regression model.

|  | Min. | Max. | Mean | Std. Dev. |
| --- | --- | --- | --- | --- |
| Basalt | 1 | 3894 | 293.17 | 391.6 |
| Phonolite | 1 | 630 | 151.15 | 177.17 |
| Quartzite | 1 | 1204 | 24.81 | 80.29 |
| Other | 3 | 260 | 85.45 | 110.35 |

**Table B. Mass by raw material.** Mean general mass values (in g) sorted by raw material.

|  | Min. | Max. | Mean | Std. Dev. |
| --- | --- | --- | --- | --- |
| Unmodified cobbles (n = 119) | | | | |
| Length | 31 | 191 | 73.55 | 25.92 |
| Breadth | 23 | 141 | 59.58 | 18.61 |
| Thickness | 12 | 81 | 43.76 | 17.17 |
| Weight | 21 | 2536 | 295.12 | 319.25 |
| Hammerstones (n = 33) | | | | |
| Length | 45 | 106 | 77.37 | 14.61 |
| Breadth | 35 | 92 | 66.65 | 12.24 |
| Thickness | 32 | 86 | 54.37 | 13.11 |
| Weight | 63 | 1120 | 397 | 204.59 |

**Table C. Typometry in cobbles and hammerstones.** Mean size and mass measurements in unmodified cobbles and hammerstones.

|  | Min. | Max. | Mean | Std. Dev. |
| --- | --- | --- | --- | --- |
| Test cores | | | | |
| Length | 52 | 204 | 82.85 | 26.76 |
| Breadth | 37 | 133 | 68.49 | 17.92 |
| Thickness | 22 | 110 | 52.63 | 16.58 |
| Weight | 77 | 3030 | 437.41 | 499.76 |
| Unifacial lineal | | | | |
| Length | 48 | 116 | 80.57 | 16.73 |
| Breadth | 37 | 99 | 67.92 | 14.63 |
| Thickness | 29 | 84 | 55.38 | 15.61 |
| Weight | 77 | 1276 | 450.23 | 299.03 |
| Unifacial orthogonal | | | | |
| Length | 58 | 83 | 69.75 | 12.14 |
| Breadth | 52 | 65 | 60 | 5.71 |
| Thickness | 30 | 58 | 45.5 | 11.59 |
| Weight | 145 | 358 | 231.5 | 92.93 |
| Bifacial lineal | | | | |
| Length | 56 | 107 | 80.79 | 13.96 |
| Breadth | 38 | 95 | 65,45 | 14.95 |
| Thickness | 26 | 75 | 50.54 | 12.93 |
| Weight | 76 | 830 | 370.12 | 205.43 |
| Bifacial alternate | | | | |
| Length | 60 | 97 | 74.8 | 14.06 |
| Breadth | 52 | 62 | 55.6 | 4.27 |
| Thickness | 41 | 59 | 45.8 | 7.52 |
| Weight | 176 | 418 | 249.6 | 98.86 |
| Bifacial orthogonal | | | | |
| Length | 35 | 103 | 66.86 | 20.9 |
| Breadth | 33 | 97 | 57.33 | 19.99 |
| Thickness | 23 | 79 | 43.6 | 16.3 |
| Weight | 35 | 944 | 268.93 | 265.46 |
| Bifacial centripetal | | | | |
| Length | 54 | 56 | 55 | 1.41 |
| Breadth | 43 | 44 | 43.5 | 0.7 |
| Thickness | 23 | 36 | 29.5 | 9.19 |
| Weight | 66 | 126 | 96 | 42.42 |
| Multifacial/multipolar | | | | |
| Length | 46 | 122 | 67.95 | 18.29 |
| Breadth | 38 | 117 | 60.52 | 19.15 |
| Thickness | 32 | 85 | 52 | 15.37 |
| Weight | 71 | 1790 | 359.23 | 396.07 |
| Bipolar technique | | | | |
| Length | 41 | 114 | 61.71 | 20.59 |
| Breadth | 30 | 112 | 49.42 | 20.28 |
| Thickness | 18 | 70 | 39.85 | 13.26 |
| Weight | 40 | 1204 | 239.42 | 301.5 |

**Table D. Typometry in cores.** Mean dimensions and mass in cores sorted by reduction pattern and technique.

|  | BA | BC | BL | BO | E | MM | T | UL |
| --- | --- | --- | --- | --- | --- | --- | --- | --- |
| BC | 0.261 |  |  |  |  |  |  |  |
| BL | 0.044 | 0.04 |  |  |  |  |  |  |
| BO | 0.238 | 0.517 | 0.008 |  |  |  |  |  |
| E | 0.371 | 0.616 | 0.238 | 0.776 |  |  |  |  |
| MM | 0.439 | 0.24 | 0.074 | 0.431 | 0.497 |  |  |  |
| T | 0.153 | 0.076 | 0.223 | 0.03 | 0.262 | 0.358 |  |  |
| UL | 0.146 | 0.085 | 0.044 | 0.383 | 0.457 | 0.488 | 0.151 |  |
| UO | 0.614 | 0.165 | 0.136 | 0.074 | 0.156 | 0.303 | 0.371 | 0.2 |

**Table E. MANOVA test.** Pairwise MANOVA test of negative scar sizes sorted by reduction strategies (p-values).

|  | Min. | Max. | Mean | Std. Dev. |
| --- | --- | --- | --- | --- |
| Whole volcanic flakes (n =53) | | | | |
| Length | 15 | 71 | 39.22 | 14.93 |
| Breadth | 15 | 74 | 37.09 | 13.15 |
| Thickness | 4 | 29 | 13.58 | 6.36 |
| Mass | 3 | 164 | 31.26 | 34.47 |
| Whole quartzite flakes (n=171) | | | | |
| Length | 13 | 76 | 30.28 | 8.7 |
| Breadth | 14 | 60 | 27.57 | 10.38 |
| Thickness | 5 | 25 | 11.19 | 3.68 |
| Mass | 4 | 94 | 12.33 | 11.84 |
| Volcanic retouched flakes (n =10) | | | | |
| Length | 32 | 57 | 44 | 7.16 |
| Breadth | 24 | 52 | 39.3 | 19.79 |
| Thickness | 9 | 23 | 17.1 | 5.08 |
| Mass | 16 | 77 | 35.2 | 19.27 |
| Quartzite retouched flakes (n=30) | | | | |
| Length | 18 | 50 | 33.5 | 8.27 |
| Breadth | 18 | 62 | 30.43 | 8.64 |
| Thickness | 7 | 22 | 14.23 | 3.72 |
| Mass | 5 | 61 | 17.96 | 11.93 |

**Table F. Typometry in detached material.** Metric and mass values of plain and retouched flakes by raw material type.

|  |  | V | Q | %V | %Q | t | *p* |
| --- | --- | --- | --- | --- | --- | --- | --- |
| Striking platform | BF | 2 | 1 | 3.4 | 0.4 | 1.5302 | 0.2161 |
|  | C | 13 | 16 | 22.0 | 6.5 | 9.2805 | 0.002316 |
|  | L | 1 | 17 | 1.7 | 6.9 | 1.8398 | 0.175 |
|  | MF | 2 | 0 | 3.4 | 0.0 | 3.5297 | 0.06028 |
|  | P | 1 | 9 | 1.7 | 3.6 | 0.22486 | 0.6354 |
|  | B | 0 | 4 | 0.0 | 1.6 | 0.17701 | 0.674 |
|  | UF | 40 | 201 | 67.8 | 81.0 | 8.1885 | 0.004216 |
| Dorsal pattern | 1 | 14 | 49 | 33.3 | 27.1 | 0.00761 | 0.9305 |
|  | 2 | 11 | 75 | 26.2 | 41.4 | 4.014 | 0.04518 |
|  | 3 | 3 | 7 | 7.1 | 3.9 | 0.098061 | 0.7542 |
|  | 4 | 3 | 7 | 7.1 | 3.9 | 0.098061 | 0.7542 |
|  | 5 | 11 | 34 | 26.2 | 18.8 | 0.16591 | 0.6838 |
|  | 6 | 0 | 9 | 0 | 5.0 | 1.3308 | 0.2487 |
| Toth type | 1 | 11 | 4 | 14.9 | 1.4 | 22.636 | 1.96E-06 |
|  | 2 | 3 | 5 | 4.1 | 1.8 | 0.51571 | 0.4727 |
|  | 3 | 4 | 9 | 5.4 | 3.2 | 0.28304 | 0.5947 |
|  | 4 | 2 | 8 | 2.7 | 2.9 | 5.45E-30 | 1 |
|  | 5 | 21 | 27 | 28.4 | 9.7 | 15.742 | 7.26E-05 |
|  | 6 | 33 | 225 | 44.6 | 80.9 | 37.598 | 8.69E-10 |

**Table G. Two-sample test for equality of proportions.** t= test statistic Pearson’s Chi-squared.

|  | Min. | Max. | Mean | Std. Dev. |
| --- | --- | --- | --- | --- |
| L. Flakes | 13 | 76 | 32.36 | 11.22 |
| L. Negatives | 15 | 76 | 32.19 | 9.23 |
| B. Flakes | 14 | 74 | 29.83 | 11.8 |
| B. Negatives | 17 | 65 | 35.08 | 9.42 |

**Table H. Flakes and negative scars.** Mean dimensions in complete flakes and negative scars recorded in cores.

| **Model 1** | | | | | |
| --- | --- | --- | --- | --- | --- |
|  |  |  |  |  |  |
| **Res. Dev.** | 2529.86 |  |  |  |  |
| **AIC** | 2545.86 |  |  |  |  |
|  |  |  |  |  |  |
| **Coeff.** |  |  |  |  |  |
|  | **Intercept** | **detached** | **mod_unmod.** | **weight_light** |  |
| Area B | - 0.014 | - 0.075 | 0.072 | 0.102 |  |
| Area C | 0.067 | - 0.089 | 0.240 | - 0.104 |  |
|  |  |  |  |  |  |
| **Std. errors** |  |  |  |  |  |
|  | **Intercept** | **detached** | **mod_unmod.** | **weight_light** |  |
| Area B | 0.188 | 0.207 | 0.286 | 0.313 |  |
| Area C | 0.184 | 0.203 | 0.277 | 0.315 |  |
|  |  |  |  |  |  |
| **P-values** |  |  |  |  |  |
|  | **Intercept** | **detached** | **mod_unmod.** | **weight_light** |  |
| Area B | 0.941 | 0.715 | 0.800 | 0.744 |  |
| Area C | 0.716 | 0.660 | 0.385 | 0.741 |  |
|  |  |  |  |  |  |
| **Model 2** | | | | | |
|  |  |  |  |  |  |
| **Res. Dev.** | 87.44 |  |  |  |  |
| **AIC** | 99.44 |  |  |  |  |
|  |  |  |  |  |  |
| **Coeff.** |  |  |  |  |  |
|  | **Intercept** | **P** | **Q** | **Bip_perc.** | **Hand_perc.** |
| Area B | 0.039 | 0.981 | 0.575 | 0.039 | 0.039 |
| Area C | 0.162 | - 0.485 | 0.901 | 0.162 | 0.162 |
|  |  |  |  |  |  |
| **Standard errors** |  |  |  |  |  |
|  | **Intercept** | **P** | **Q** | **Bip_perc.** | **Hand_perc.** |
| Area B | 0.162 | 1.253 | 1.317 | 0.162 | 0.162 |
| Area C | 0.150 | 1.483 | 1.205 | 0.150 | 0.150 |
|  |  |  |  |  |  |
| **P-values** |  |  |  |  |  |
|  | **Intercept** | **P** | **Q** | **Bip_perc.** | **Hand_perc.** |
| Area B | 0.808 | 0.434 | 0.662 | 0.808 | 0.808 |
| Area C | 0.280 | 0.744 | 0.455 | 0.280 | 0.280 |

**Table I. Multinomial regression models.** Coefficients, standard errors and p-values for the coefficients of the levels of the factor variables included in each multinomial regression model.


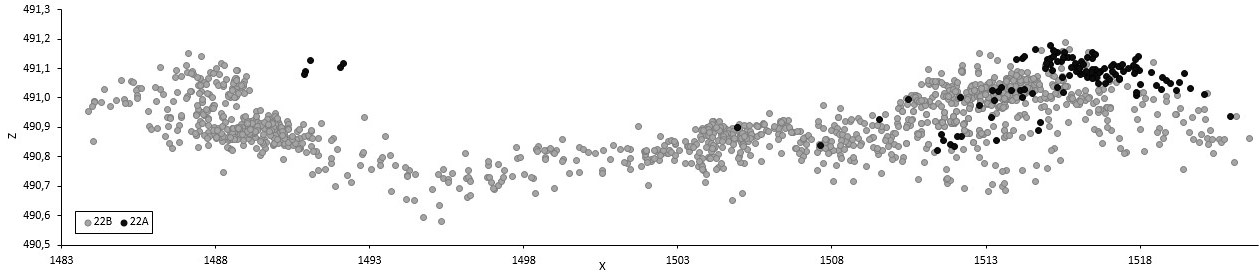


**Fig A.** **Vertical projection (XZ axis) of the lithic material plotted at DS**. Field identification of Levels 22A and 22B is shown in the vertical distribution.


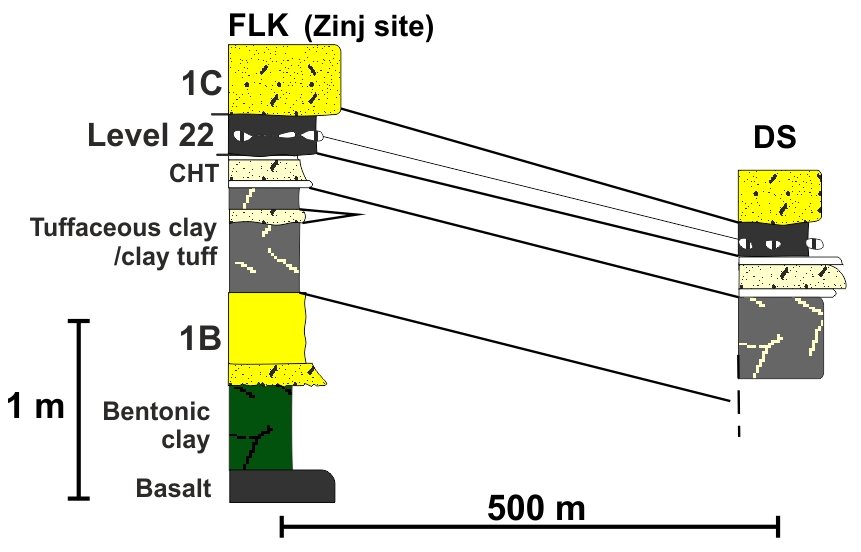


**Fig B. Stratigraphic correlation of the Zinj Palaeolandscape in Bed I at FLK and DS** (1B= Tuff 1B, 1C= Tuff 1C, CHT= Chapati Tuff).

**
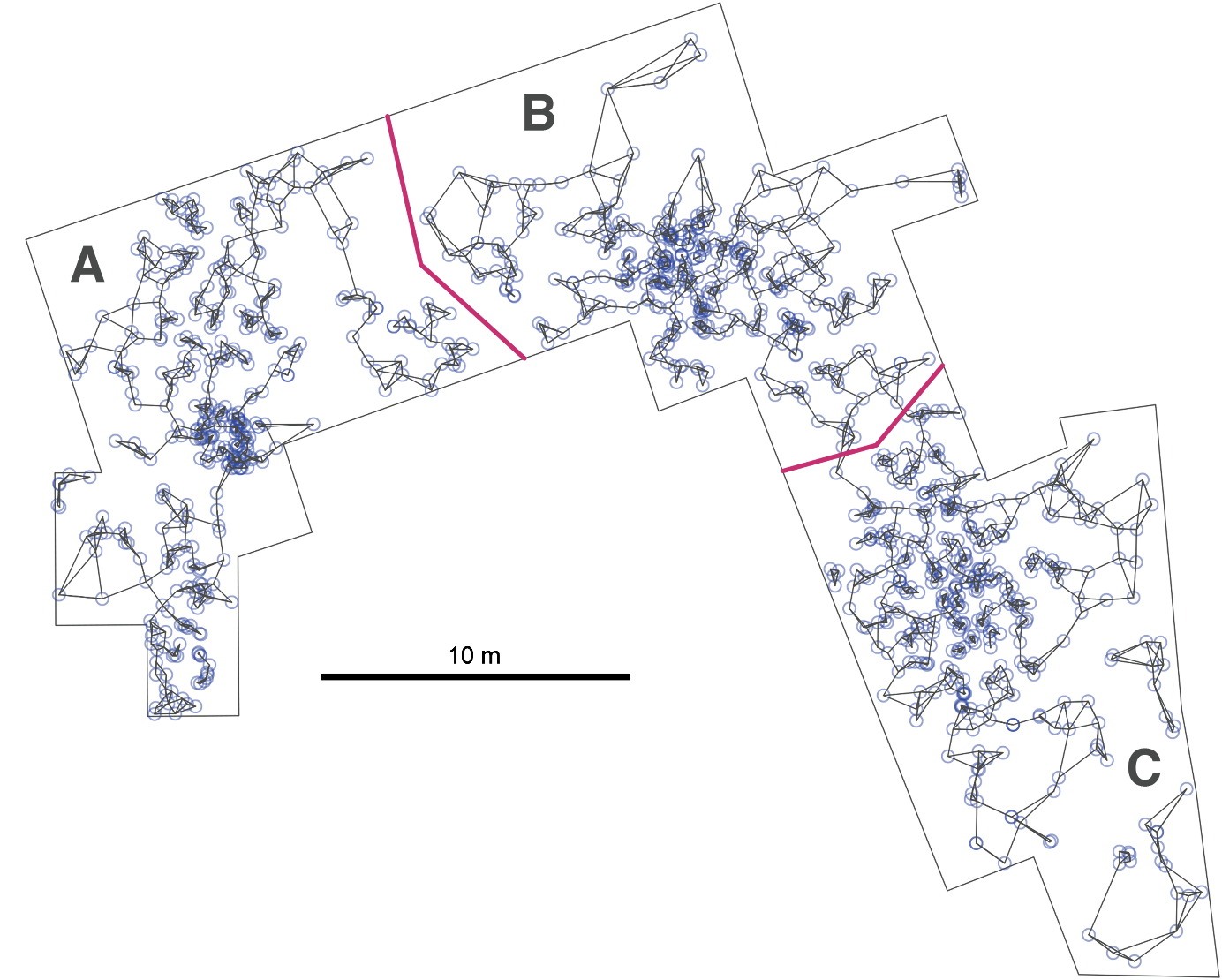
**

**Fig C**. **K-nearest neighbor graph of the DS lithics point pattern generated with a K value of 3**. Pink lines through the emptiest areas show the subdivision of the point process into point patterns A, B and C.

**Fig D. Percentage contribution of mass (in g) to lithic categories and raw material type.** Categories (U=unmodified, P=percussion, C=cores; D=detached, R=retouched; CH=chopper-cores, W=waste). Raw material type (B=basalt, P=phonolite, Q=quartzite, O=other).


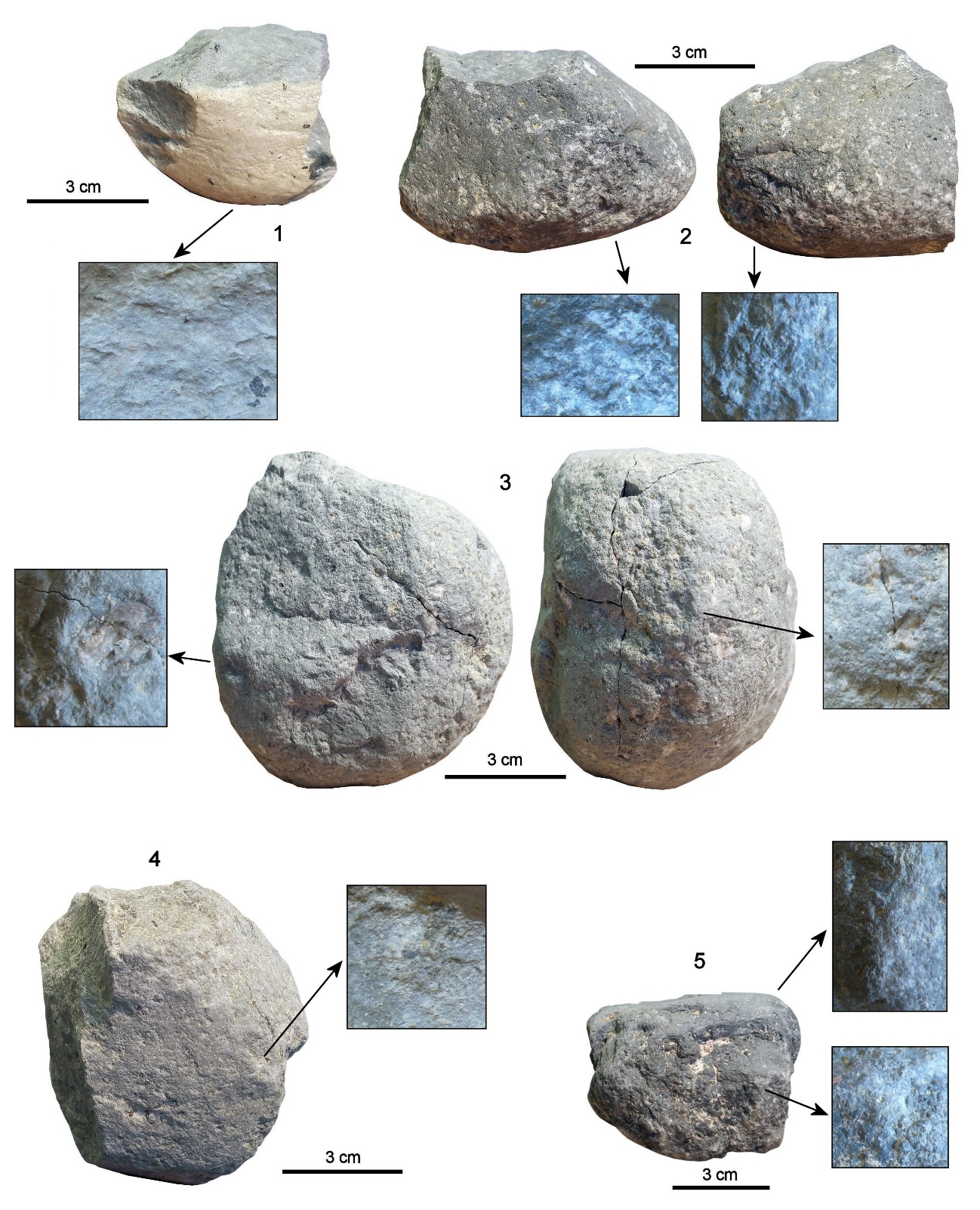


**Fig. E.** **Close-ups of the percussion battering identified on various basalt specimens from DS.** 1, 2, 4 and 5: battering and pitting observed on cortical areas of cores; 3: Pitting and battering on a hammerstone.

**Fig F**. **Length and width scatterplot of cores sorted by raw material type** (V= volcanic; Q= Quartzite).

**Fig G. Percentage contribution of raw materials to simplified handheld reduction models.** V= Volcanic; Q= Quartzite, T= test/unorganized; U= Unifacial; BS= Bifacial simple (BL and BA models); BP= Bifacial progressive (BO and BC models); MM= Multifacial multipolar; E= Exhausted cores.


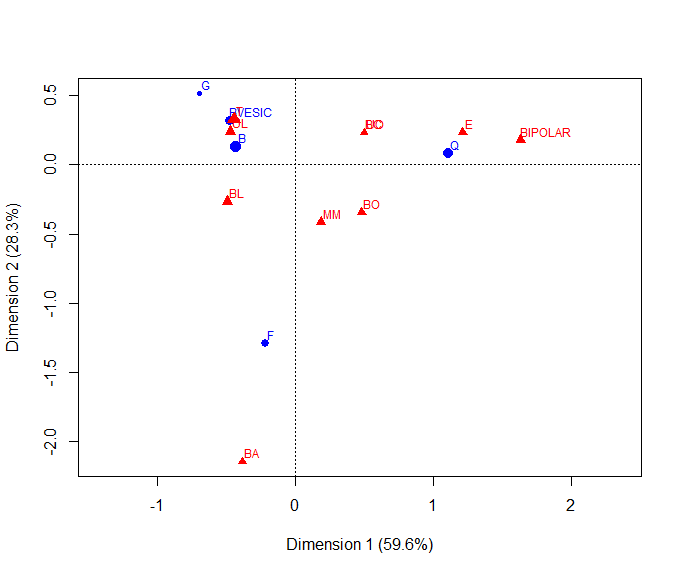


**Fig H**. **Multiple Correspondence Analysis (MCA) between variables and core categories**. Variables (circles): B=basalt; BVESIC=Vesicular basalt; F=phonolite; Q=quartzite; G=gneiss). Core categories (triangles): UL=unifacial linear; UO=unifacial orthogonal; BL=bifacial linear; BA= bifacial alternate; BO= bifacial orthogonal; BC= bifacial centripetal; MM= multifacial multipolar; E=exhausted; B=bipolar.


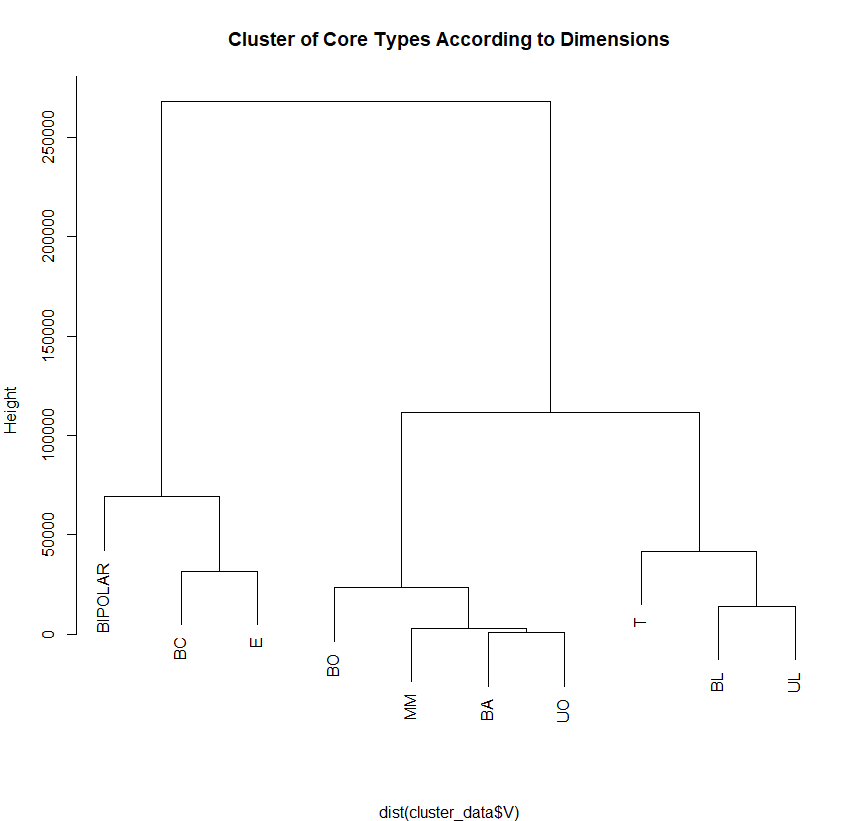


**Fig I**. **Cluster analysis of core types according to volume (length x breadth x thickness)**. UL=unifacial linear; UO=unifacial orthogonal; BL=bifacial linear; BA= bifacial alternate; BO= bifacial orthogonal; BC= bifacial centripetal; MM= multifacial multipolar; E=exhausted; B=bipolar.


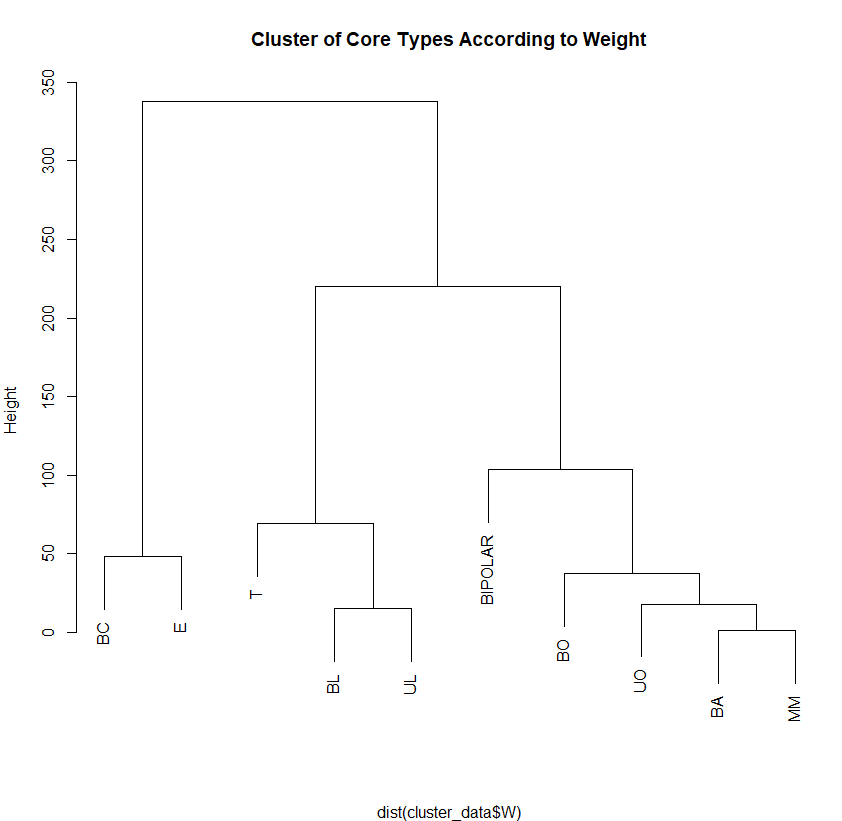


**Fig J**. **Cluster analysis of core types according to mass (in g)**. UL=unifacial linear; UO=unifacial orthogonal; BL=bifacial linear; BA=bifacial alternate; BO= bifacial orthogonal; BC=bifacial centripetal; MM=multifacial multipolar; E=exhausted; B=bipolar).

**Fig K**. **Scatterplot of negative scars detached from cores, sorted by raw material type**. B=basalt; P=phonolite; Q=quartzite.

**Fig L**. **Percentage contribution of cores with percussion damage sorted by simplified reduction model.** T=test/unorganized; US= Unifacial simple; BS= bifacial simple; BC= Bifacial complex; MM= multifacial.

**Fig M**. **Complete plain flake frequency (n) sorted by maximum length groups (mm) and raw material type**. V=Volcanic; Q=Quartzite.

**Fig N**. **Percentage contribution of Toth types by raw material**. V= Volcanic; Q=Quartzite.

**Fig O**. **Percentage contribution of dorsal patterns on plain flakes by raw material**. Pattern: 1= one direction or linear; 2= two unorganized directions; 3= opposed longitudinal; 4= opposed lateral; 5= orthogonal; 6= centripetal). Raw material: (V=volcanic; Q=quartzite).

**Fig P**. **Percentage distribution of striking platforms by raw material.** Platforms: C=cortical; UF=unifaceted; BF=bifaceted; MF=multifaceted; L=line; P=point; B=broken. Raw material: V=volcanic; Q=quartzite.

**Fig Q**. **Flakes and negative scars.** Scatterplot of length and breadth in complete flakes (F) and negative scars (N).


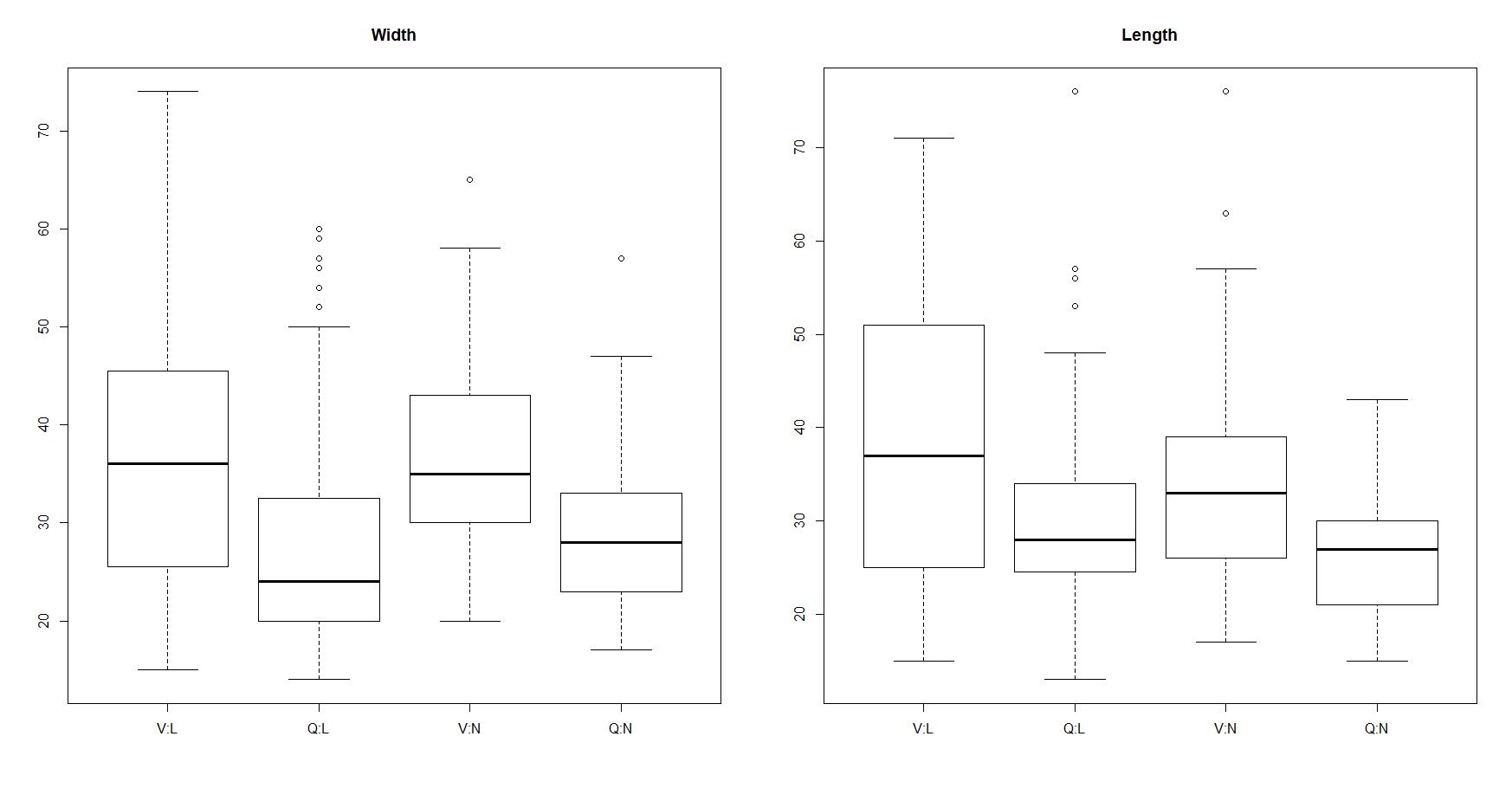


**Fig R.** **Length and breadth boxplots of complete flakes and negative scars sorted by raw material type**. VL=volcanic flake; QL=quartzite flake; VN=volcanic negative; QN= quartzite negative.

**Fig S**. **Retouched types**. Percentage contribution (and counts in brackets) of morpho-types of retouched flakes.


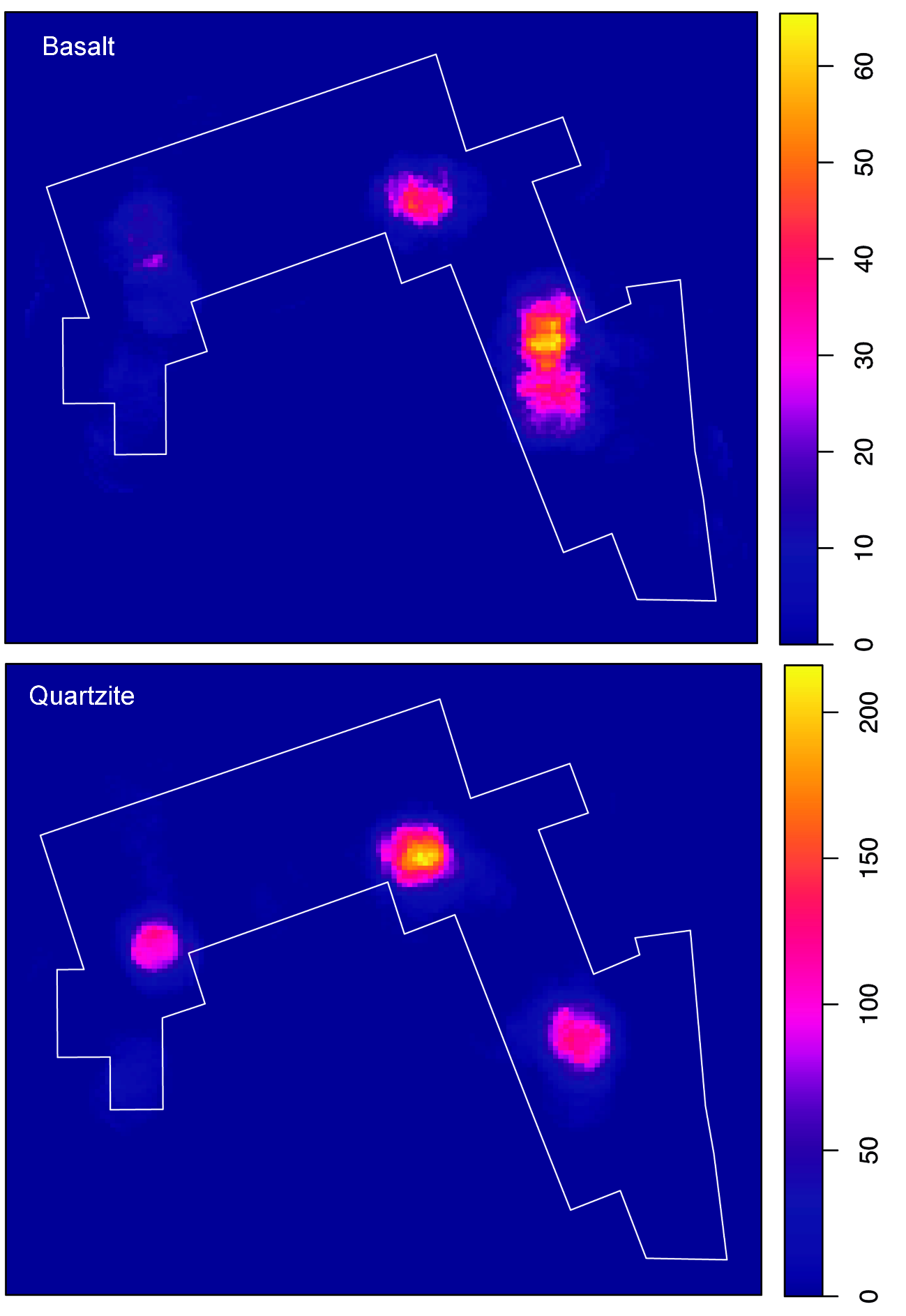


**Fig T**. **Hot spot maps of raw material.** Basalt and quartzite lithic implements.


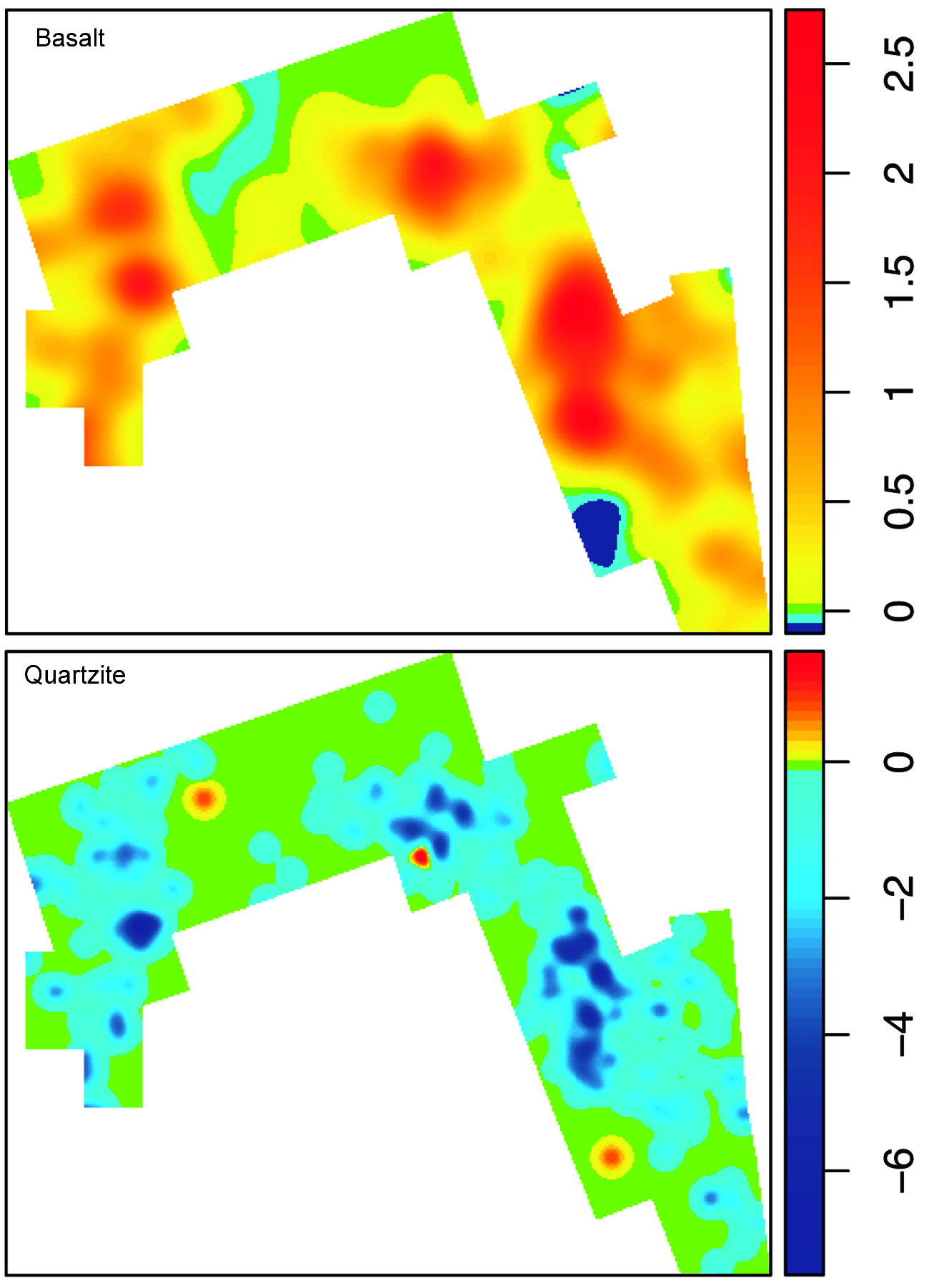


**Fig U.** **Density maps by mass**. Density maps of light (<400 g) basalt materials and heavy (>400 g) quartzite materials.


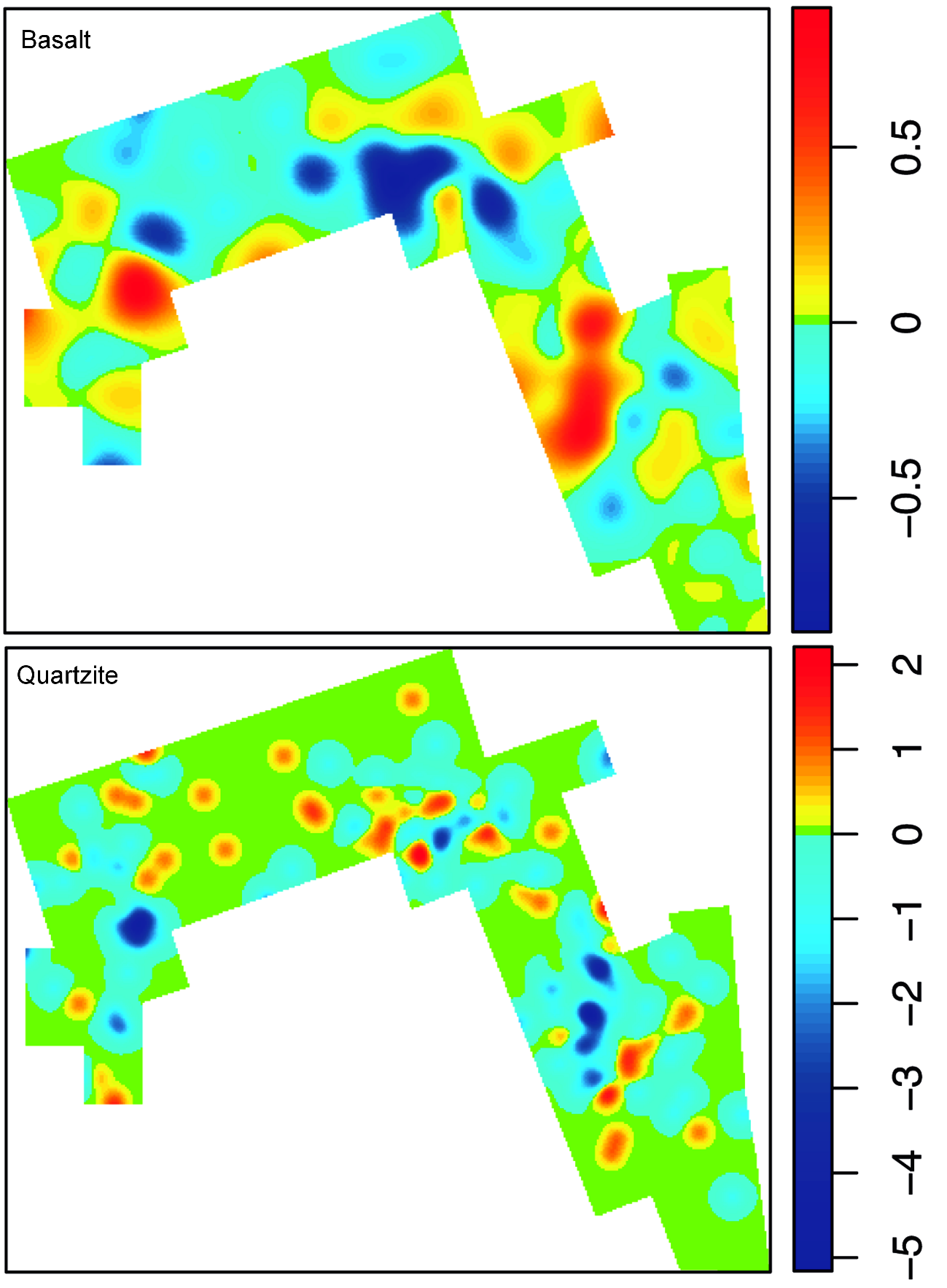


**Fig V**. **Density maps by mass.** Density maps of heavy (>400 g) basalt materials and light (<400 g) quartzite materials.


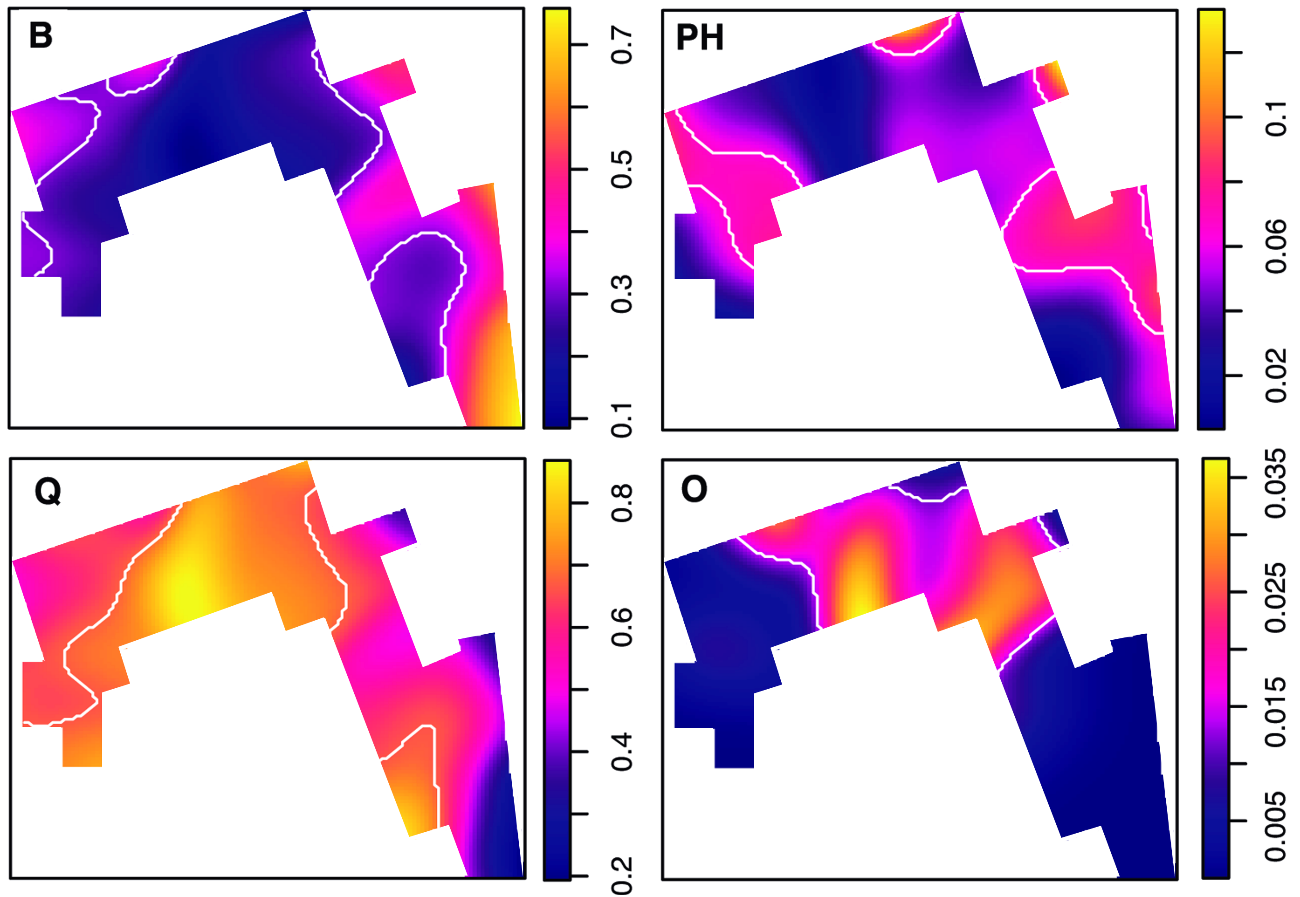


**Fig W. Spatial probability (relative-risk) maps of the different raw material types**. Tolerance contours (white) show areas of significant deviation from the average proportion. (B=basalt; Ph=phonolite; Q=quartzite; O=Other: gneiss, chert, rock crystal).


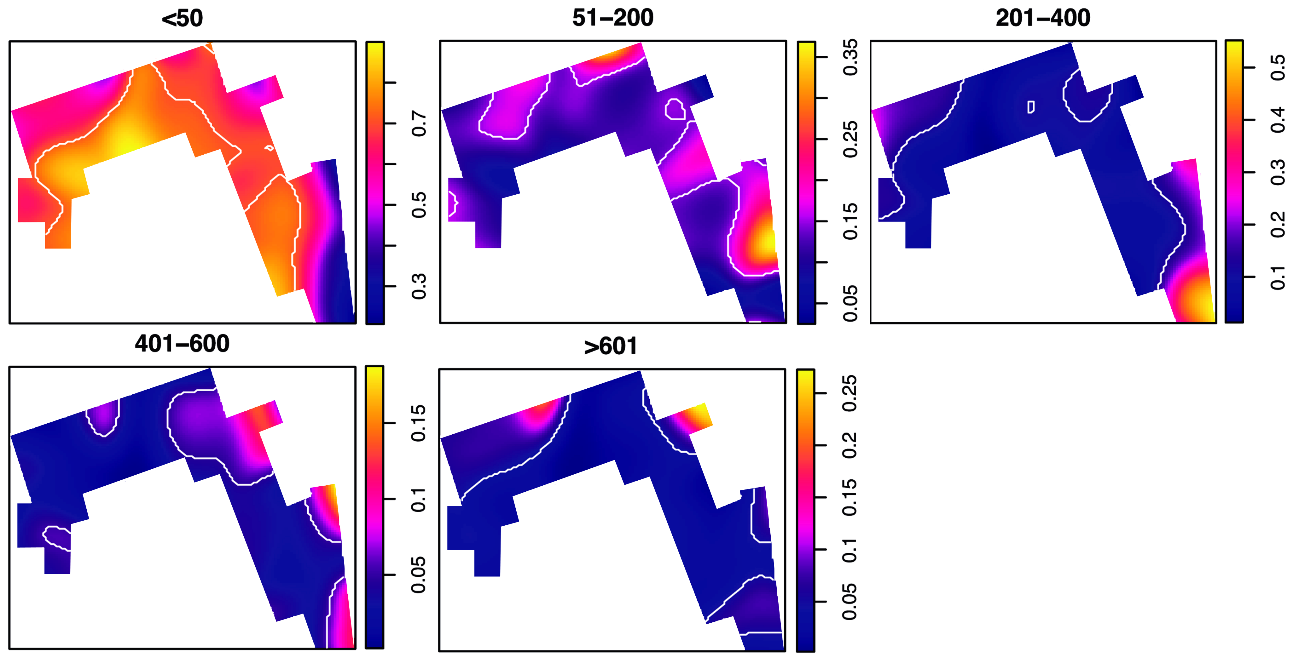


**Fig X**. **Spatial probability (relative-risk) maps of mass (in g) classes**. Tolerance contours (white) show areas of significant deviation from the average proportion.

**
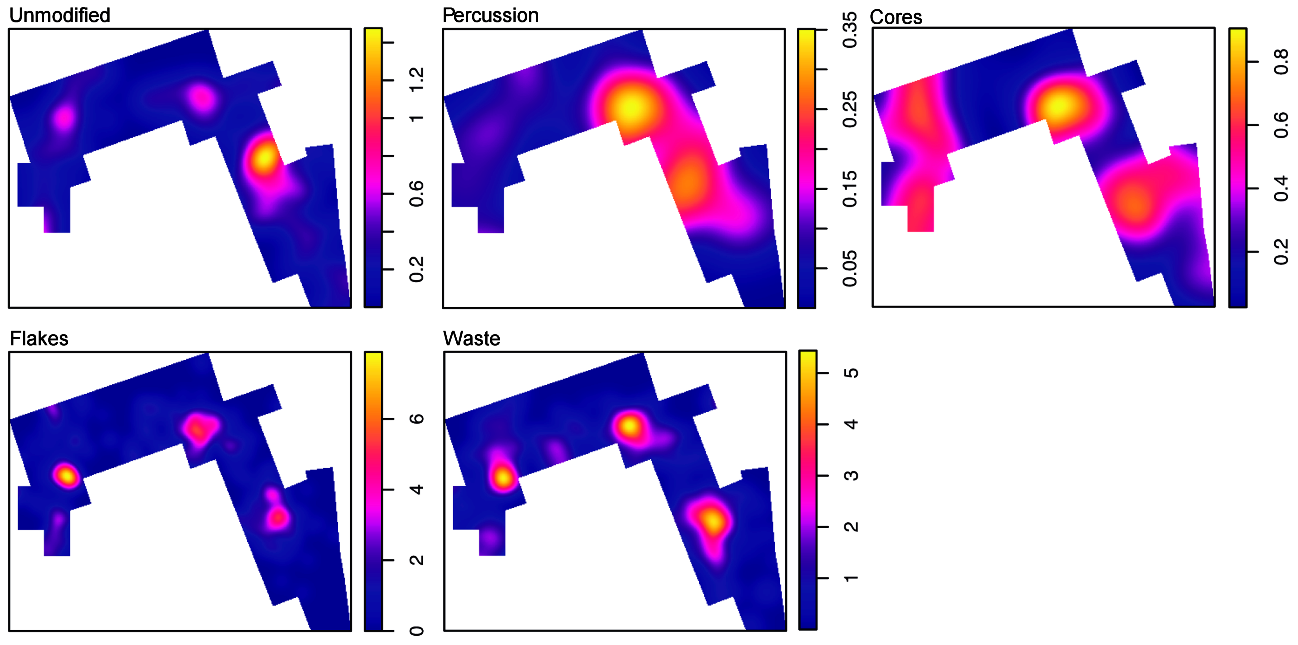
**

**Fig Y.** **Density map of lithic categories.** Unmodified material, percussive material, cores, detached material (flakes and retouched flakes), waste (debris, shatter, fragments).


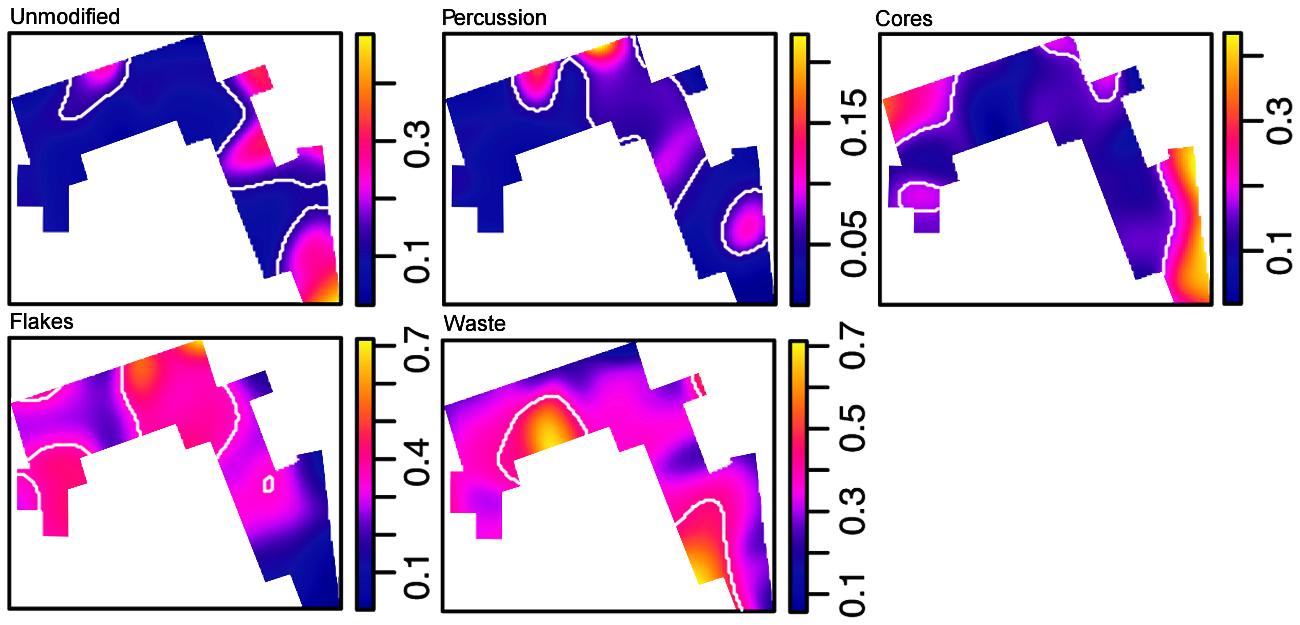


**Fig Z**. **Spatial probability (relative-risk) maps of lithic categories**. Tolerance contours (white) show areas of significant deviation from the average proportion.

**
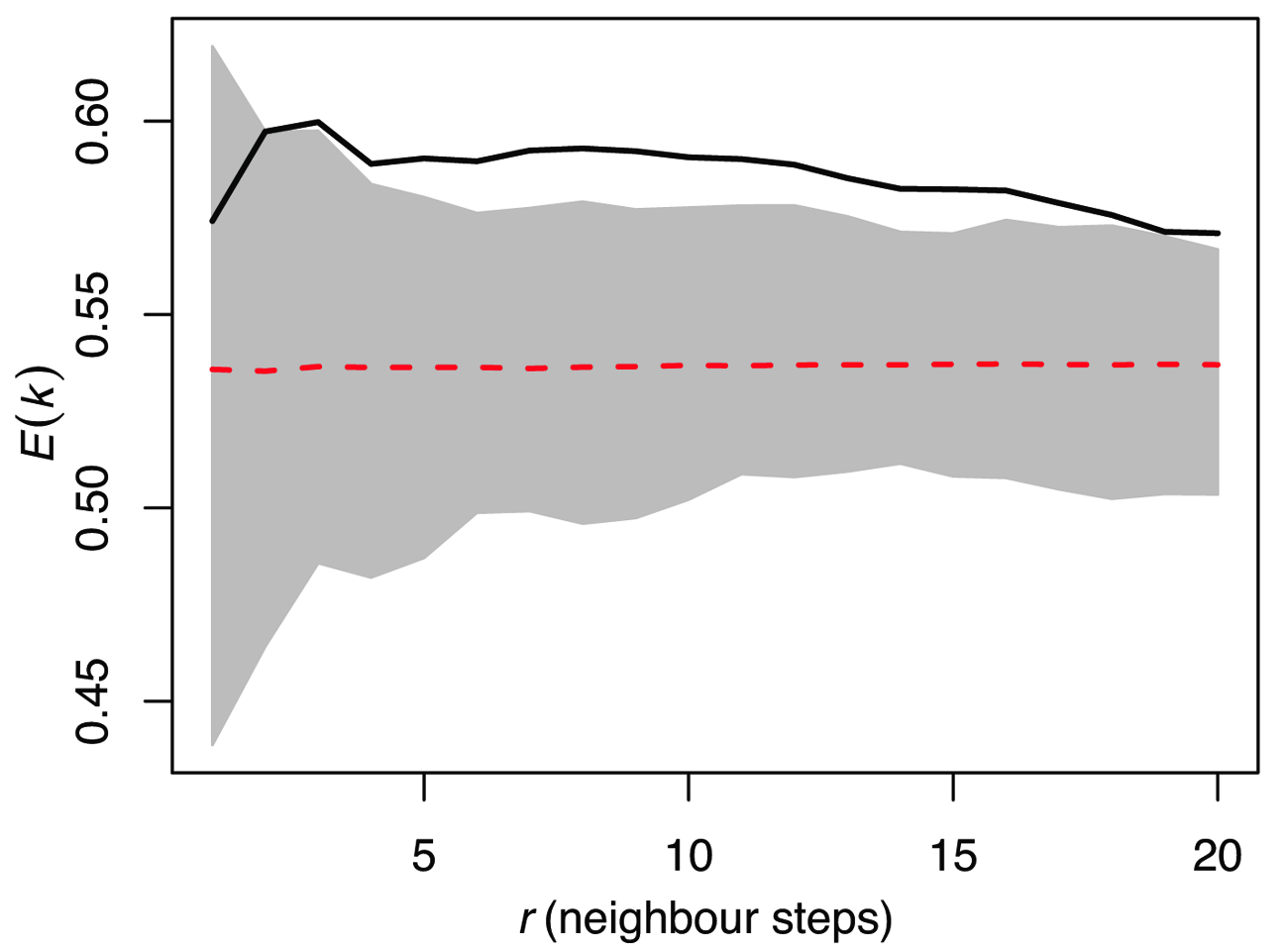
**

**Fig AA**. **Nearest neighbor equality function plot for the point processes of modified and unmodified materials showing the cumulative proportion of neighbors of the same type**. Black line shows that the cumulative proportion of neighbors of the same type is higher than expected if the two spatial distributions were randomly mixed, because it falls outside the confidence interval (gray band) of randomly mixed processes (red dotted line).


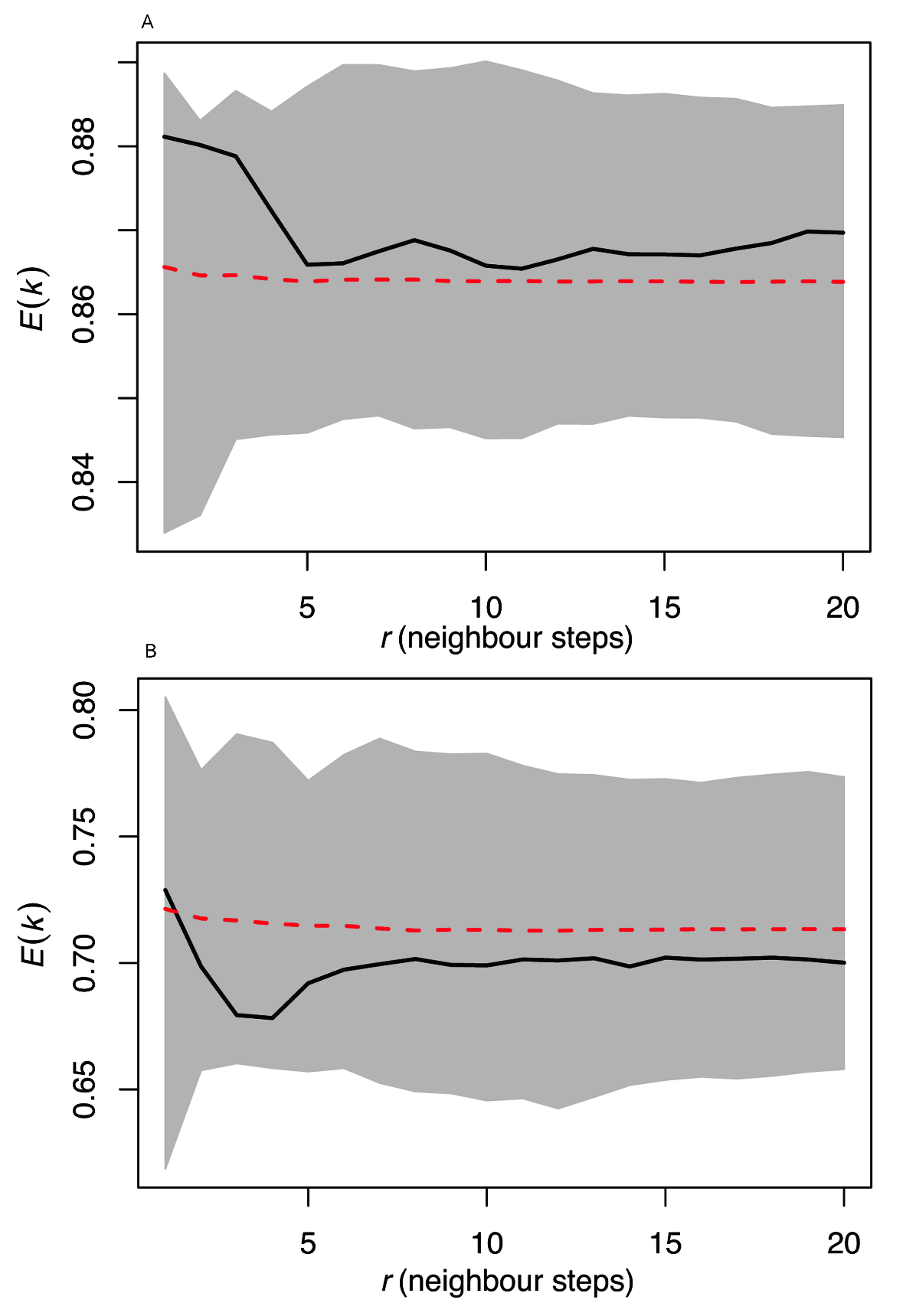


**Fig AB**. **Nearest neighbor equality function plot for the point processes**. A. Percussion and handheld knapping materials showing the cumulative proportion of neighbors of the same type. B. Handheld cores with percussion damage and the rest of handheld cores. In both graphs the relationship between the two-point processes is statistically non-significant, as the black line falls inside the gray confidence intervals.

**
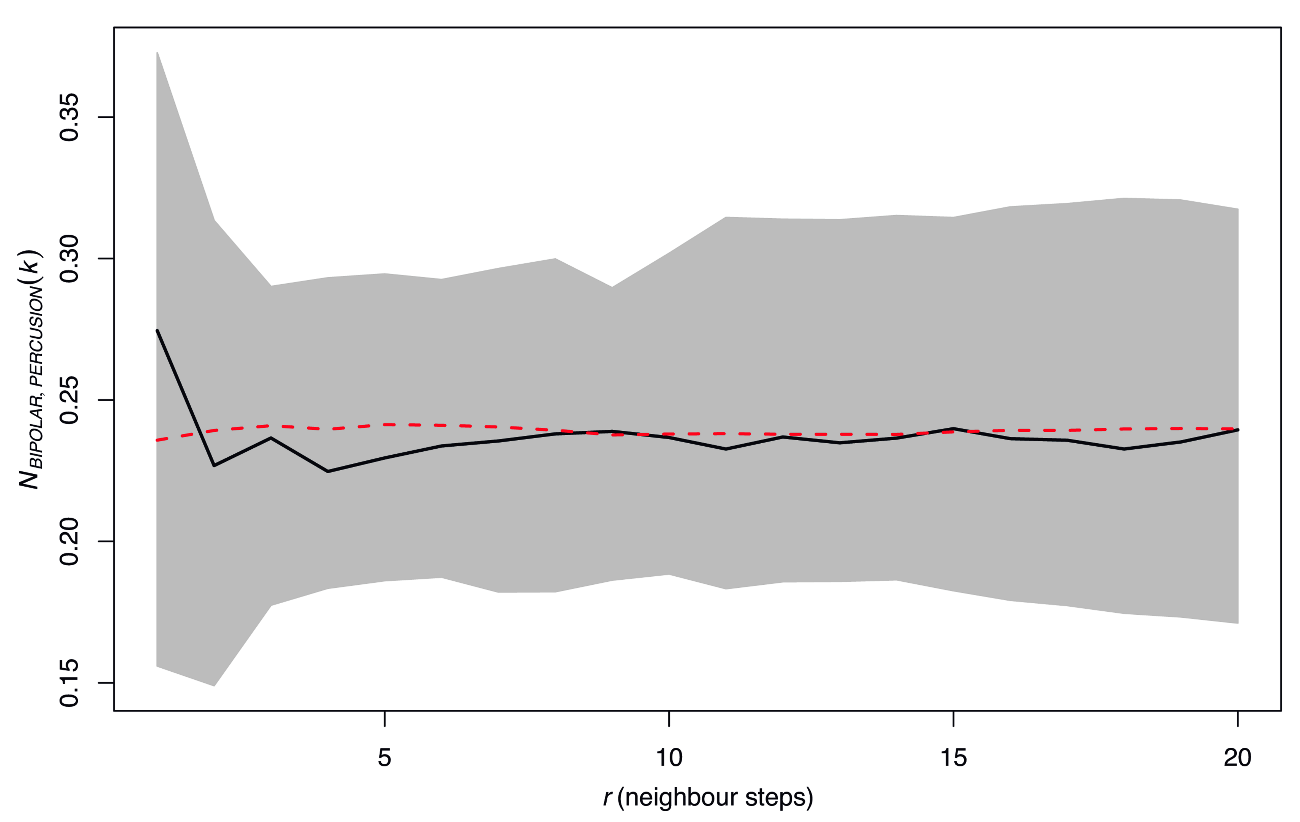
**

**Fig AC**. **Nearest neighbor equality function plot for the point processes** **of percussion and bipolar materials showing the cumulative proportion of neighbors of the same type**. Both spatial point processes are statistically similar.

**
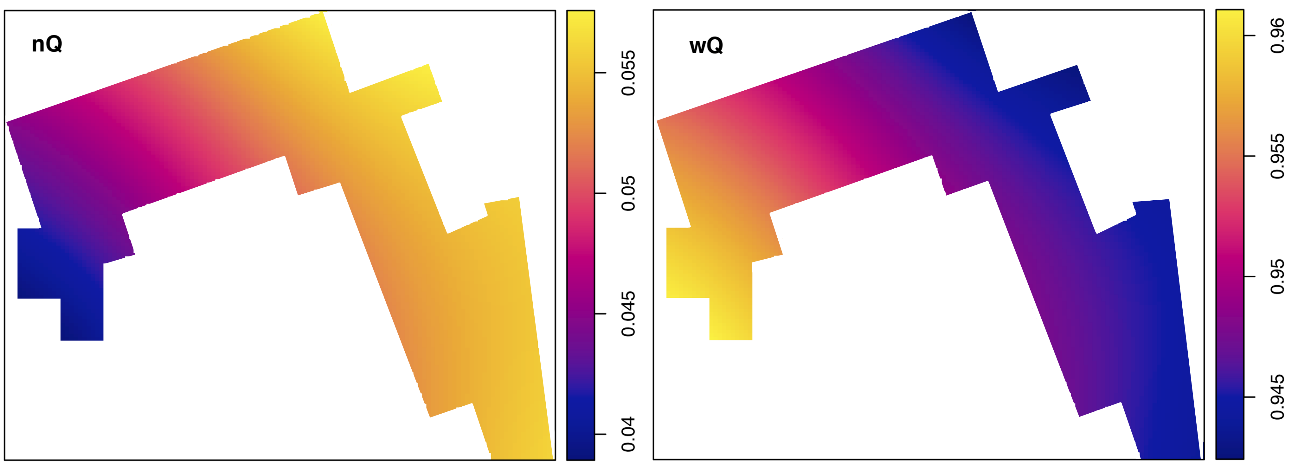
**

**Fig AD**. **Spatial probability (relative-risk) maps of quartzite cores and quartzite waste**. The absence of tolerance contours shows that there are no areas in which one type of probability is significantly higher than the average proportion.

**
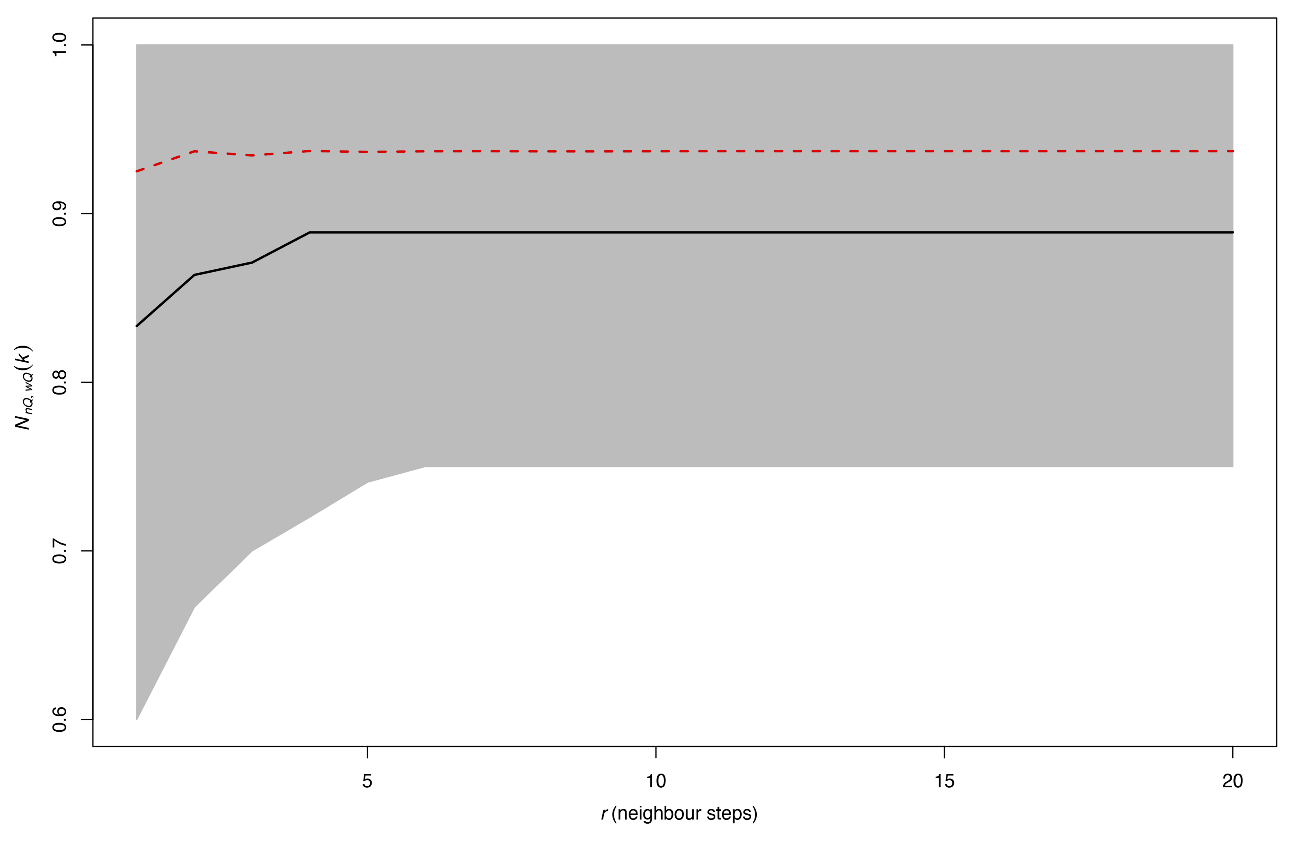
**

**Fig AE**. **Nearest neighbor equality function plot for the point processes of waste and freehand cores showing the cumulative proportion of neighbors of the same type**. Both spatial point processes are statistically similar to randomly mixed point processes.


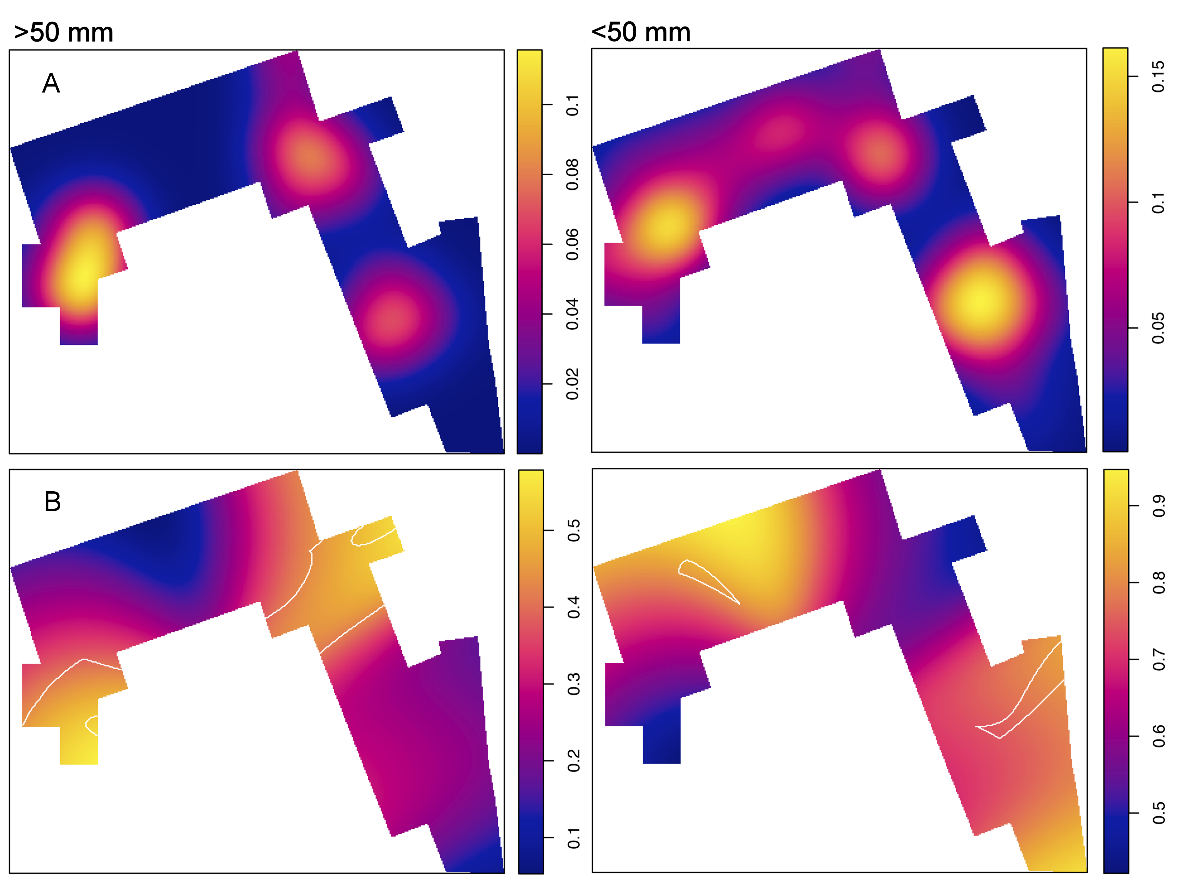


**Fig AF.** **Cutting edges**. A. Density maps of the spatial distribution of flakes according to the available cutting edge (>50 mm and ≤50 mm). B. Spatial probability (relative-risk) maps of flakes by cutting edge length class.


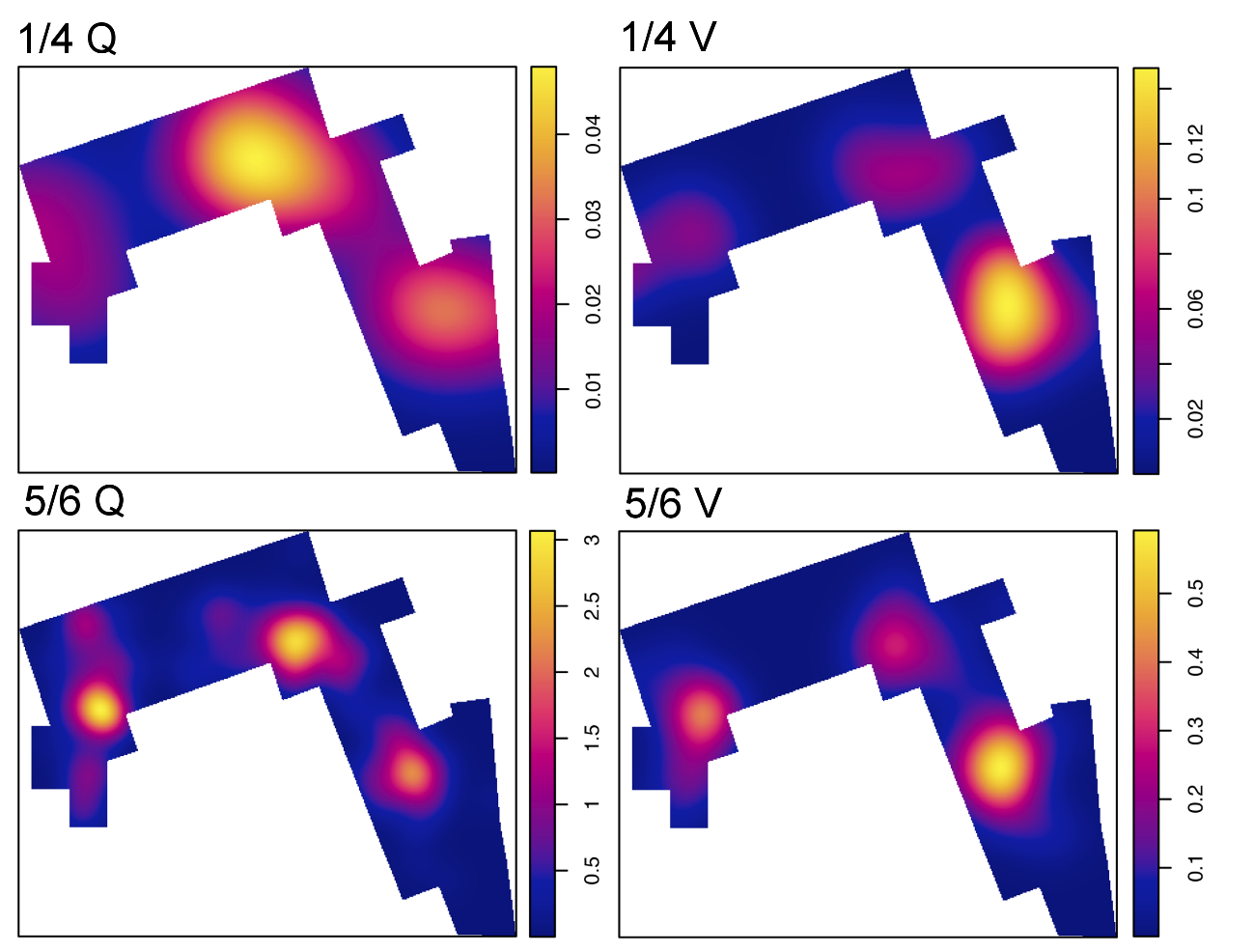


**Fig AG**. **Toth’s types.** Density maps of the spatial distributions of different Toth’s flake types (1/4 and 5/6) according to raw material type (q=quartzite, v=volcanic).
